# Development of small cyclic peptides targeting the CK2α/β interface†

**DOI:** 10.1039/d2cc00707j

**Published:** 2022-03-21

**Authors:** Eleanor L. Atkinson, Jessica Iegre, Claudio D’Amore, Paul Brear, Mauro Salvi, Marko Hyvönen, David R. Spring

**Affiliations:** Yusuf Hamied Department of Chemistry, University of Cambridge Lensfield Road CB2 1EW Cambridge UK spring@ch.cam.ac.uk; Department of Biomedical Sciences, University of Padova Padova Italy mauro.salvi@unipd.it; Department of Biochemistry, University of Cambridge Tennis Court Road CB2 1GA Cambridge UK mh256@cam.ac.uk

## Abstract

In this work, an iterative cycle of enzymatic assays, X-ray crystallography, molecular modelling and cellular assays were used to develop a functionalisable chemical probe for the CK2α/β PPI. The lead peptide, P8C9, successfully binds to CK2α at the PPI site, is easily synthesisable and functionalisable, highly stable in serum and small enough to accommodate further optimisation.

## Abstract 2

Development of a small CK2α/β protein–protein interaction-inhibiting peptide, which is easily synthesisable and functionalisable, with good enzymatic and cellular activity.

## Graphical abstract

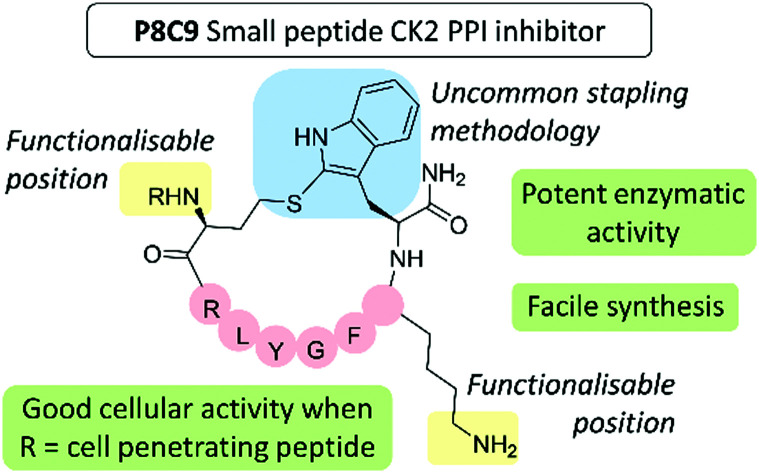

CK2 is a ubiquitous and constitutively active protein kinase.^1^ It has a heterotetrameric quaternary structure, consisting of two catalytic subunits (α or α′) and two regulatory subunits (β).^2^ The α/α′ subunits carry out the phosphorylation of target proteins and peptide substrates, whereas the β subunits confer stability to the enzyme, control substrate specificity, and govern cellular localisation of the holoenzyme complex.^3^ In particular, CK2β enhances the catalytic activity of CK2α, as well as increasing its thermostability.^4^ CK2β is also necessary for the protein shuttling of CK2 between intracellular compartments and, consequently, it controls the cellular location of the enzyme. Removal of CK2β from the catalytic subunit prevents the formation of the holoenzyme, resulting in adverse effects upon substrate recognition, the stability of CK2α, and its intracellular protein shuttling, all altering CK2's effect on the cell cycle.^5^

CK2 acts as an anti-apoptotic protein and is seen to be overexpressed in a wide variety of cancerous tumours.^3,6–8^ The absence of alternative pathways to compensate for CK2 downregulation makes cancer cells particularly sensitive to CK2 inhibition.^9–11^ Promisingly, a first-in-class small molecule inhibitor, CX4945, has been designated as an orphan drug by FDA for the treatment of cholangiocarcinoma, and several clinical studies are ongoing for the treatment of different types of tumours with this molecule, both alone and in combination therapies.^12^

There are multiple ways in which CK2 can be inhibited and the most widely researched of these methods is to target the ATP-binding site, which can be done with the aforementioned inhibitor CX4945.^3^ More recently, however, there has been a move towards targeting alternative sites on the protein.^13–15^ One such alternative method is *via* the inhibition of the protein–protein interaction (PPI) between the α and β subunits of CK2; the PPI interface is not as well conserved among other kinases as the ATP active site hence its inhibition is considered a more selective approach.^2^ Preventing the formation of the CK2 holoenzyme would induce apoptosis by adjusting the substrate specificity and cellular location of the subunits, thus altering the intracellular environment.^16^

Peptides are good PPI modulators as their flexibility and size allows them to adapt to the large and shallow surfaces involved.^17^ This strategy has been applied to target CK2, and a handful of cyclic peptides are known to inhibit the CK2 PPI, most notably Pc, TAT-Pc and CAM7117.^16,18,19^ The most potent inhibitor, CAM7117, shows an improved *K*_d_ relative to Pc (200 nM *vs.* 1 μM respectively), greater stability in human serum and antiproliferative activity in cancer cells.^16^ However, the synthesis of CAM7117 requires the use of an expensive unnatural amino acid, a non-commercially available staple, and three purification steps; thus the synthesis is expensive, slow and low yielding.^16^ Despite currently being the most potent peptide PPI inhibitor of CK2, its reduced activity in cellular assays and complex synthesis indicates that further work is required to develop a potent and selective peptide which inhibits CK2 in cells and is facilely obtained.

We are building here on the CAM7117 work, developing novel conformationally-constrained peptides which are easily synthesised and with improved physicochemical properties to yield an enhanced chemical probe for the CK2 PPI.

Following iterative cycles of design, synthesis and testing, the sequence of CAM7117 was shortened. A lead sequence, able to bind to CK2α and simultaneously present the side chains of two amino acids in a suitable position for constraining, was identified before being cyclised using a variety of constraints to obtain a peptide locked in its binding conformation (Fig. 1). The binding mode of the lead cyclic peptide was investigated using X-ray crystallography of the ligand in complex with CK2α. This peptide was then tested in cellular assays to evaluate its ability to reduce cell viability.

**Fig. 1:**
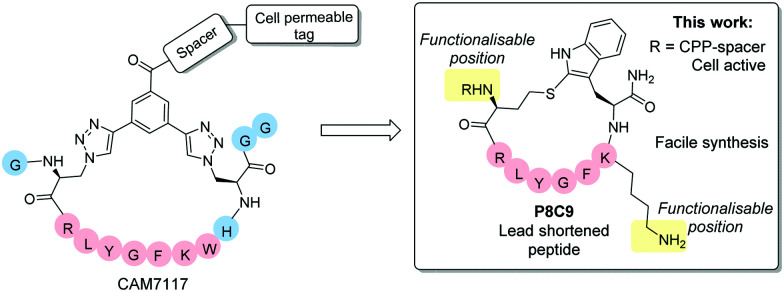
Overview of the lead peptides developed in this work compared to the CK2 PPI inhibitor CAM7117.^16^ The shortened peptide P8C9 is easily synthesisable, active in enzymatic assays and functionalisation with a cell penetrating peptide (CPP)-spacer yields good cellular activity. Blue spheres = residues removed from CAM7117 in P8C9.

Retention of good binding affinity, combined with a reduction in the molecular weight, resulted in an increase in the ligand efficiency (LE) of the peptide, whilst addition of a cell penetrating tag provided good cellular activity, with all peptides being facile to synthesise in two steps without needing synthetic staples and complex unnatural amino acids.

Analysis of the X-ray crystal structure of unfunctionalised CAM7117 (sequence GX_C1_RLYGFKWHX_C1_GG) bound to CK2α (PDB: 6Q4Q) strongly suggests that the key binding interactions between the peptide and CK2α are focused within the cyclic portions of the peptide.^16^ Therefore, shortened peptides using the central binding sequence of CAM7117 (RLYGFKWH) were chosen to investigate which residues could be used for cyclising the peptide into its binding conformation.

Molecular modelling suggested that only two positions (other than those used in CAM7117) could be used for constraining the central peptide sequence, RLYGFKWH: Trp and Aza-alanine (Aza) residues (Fig. S1, ESI†). Cyclisation of the Trp and Aza residues would allow for even greater shortening of the peptide sequence than simple removal of the terminal Gly residues. Additionally, the distance between the α-carbons of the Trp and Aza residues (7.5 Å) is shorter than between the two Aza residues in CAM7117 (11.6 Å) meaning that the size of the constraint used could also be reduced (Fig. S1, ESI†).

Before proceeding to the synthesis of constrained peptides, a series of linear peptides was synthesised based upon the central sequence of CAM7117 (RLYGFKWH) to determine which amino acids could be removed without compromising activity.

The peptide sequences initially synthesised are outlined in Table 1 alongside that of unfunctionalised CAM7117 (P_+_, CAM7117 without the tri-Arg tag and spacer). Additionally, a reverse-sequence peptide, P_−_ (HWKFGYLR), was synthesised for use as a negative control in subsequent assays.

**Table 1:** Linear peptide sequences synthesised and the number of residues they contain. All peptides feature an amide at the C-terminus and are acetyl-capped at the N-terminus. X = Aza-alanine. X_C1_ = unfunctionalised constraint in CAM7117

Peptide	Sequence	No. of residues	IC_50_ (μM)
P1	RLYGFK	6	>500
P2	LYGFKW	6	136.9 ± 25.8
P3	RLYGFKW	7	22.5 ± 2.4
P4	RLYGFKWH	8	16.7 ± 2.3
P_+_	GX_C1_RLYGFKWHX_C1_GG	13	8.7 ± 4.2

To assess the ability of the synthesised linear peptides to displace a CK2β-like fluorescent probe from CK2α, a fluorescence polarisation (FP) assay was used (Table 1, ESI 1.3.2 and Fig. S2, ESI†). Comparison of the linear peptides indicates a large drop off in binding with the removal of the Arg (P2*versus*P3) and Trp residues (P1*versus*P3), suggesting that they both contribute positively to the activity of the peptide, with the removal of Trp resulting in a more profound loss of activity. The Ile to Trp mutation in CAM7117 resulted in a boost in potency (*K*_d_ = 150 nM, *K*_d_ = 460 nM for P_+_ and the corresponding peptide bearing Ile instead of Trp).^16^ Therefore, it is unsurprising that removal of the Trp residue resulted in a large loss in activity. Conversely, the His does not appear to be crucial to the binding ability of the peptide and, therefore, it was removed in the design of cyclic peptides.

Combining the results of the molecular modelling, crystal structures and the FP of the linear peptides, the design of cyclic peptides was based upon the shortened peptide sequence RLYGFK, with two flanking residues for cyclisation at either end. Although the removal of Trp led to a loss in activity of the linear peptide, it was envisaged that its binding contribution could be compensated for by utilising an aromatic or hydrophobic staple, mimicking the properties of the Trp residue. For this purpose, the linear sequences CRLYGFKC (P5) and JRLYGFKX (P6) were used (J = propargylglycine, X = Aza-alanine) with a variety of constraints. Additionally, we considered keeping the Trp residue and stapling with Cys and homoCys (hC) residues using the linear sequences CRLYGKW (P7) and (hC)RLYGKW (P8) respectively with mild oxidative cyclisation conditions.^20^ Furthermore, due to the small distance between the cyclising residues (7.9 Å), large constraints such as long alkyl chains and extended aromatic ring structures were ruled out. Instead, relatively small constraints were trialled (Table S1, ESI†).

The eight cyclic peptides synthesised were tested in an FP assay (Table 2 and ESI† Fig. S3). Both the triazole stapled peptides, P6C2 and P6C3, showed negligible inhibition. P5C4, P5C5, P5C7 and P7C8 all showed reasonable activity, with IC_50_ values between 15.6 and 54.6 μM. The methylene bridge-stapled peptide P5C6 also showed distinct activity, although it was not quantifiable due to poor solubility.‡‡Pleasingly, P8C9 showed good activity (1.7 ± 0.3 μM) and was used as a starting point for further optimisation.

Compared to P_+_, P8C9 showed a greater than 5 times increase in activity in our preliminary assay (Fig. S3–S5, ESI†), indicating that P8C9 can efficiently engage CK2α despite its reduced size. A comparison of the LEs (calculated as outlined in eqn (S1), ESI†) of the novel peptides with that of P_+_ indicates that P8C9 uses its structural features to target CK2α the most effectively with a LE of 0.101 *vs.* 0.057 for P_+_ (Table 2).

**Table 2:** Structure of the active cyclic peptides tested, alongside their IC_50_'s and ligand efficiencies (LEs). IC_50_'s are reported ±SEM. Peptide sequences: P5CX = C_CX_RLYGFKC_CX_, P7C8 = C_C8_RLYGFKW_C8_, P8C9 = (hC)_C9_RLYGFKW_C9_, P_+_ = GX_C1_RLYGFKWHX_C1_GG

Peptide	Constraint	IC_50_ (μM)	LE
P5C4	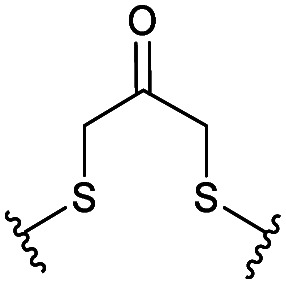	54.6 ± 11.2	0.080
P5C5	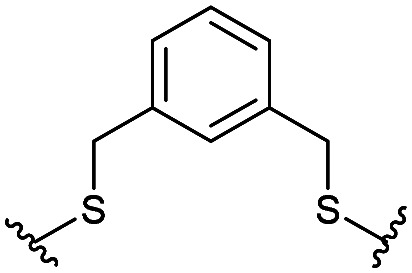	28.9 ± 1.9	0.080
P5C7	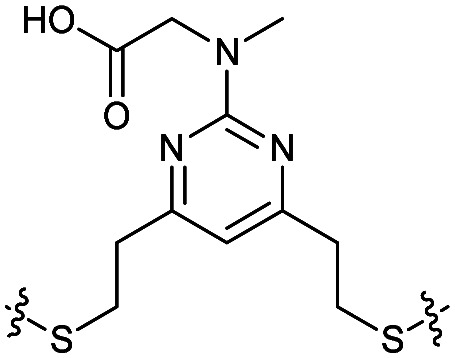	40.0 ± 4.7	0.071
P7C8	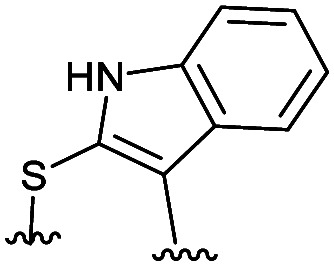	15.6 ± 5.1	0.085
P8C9	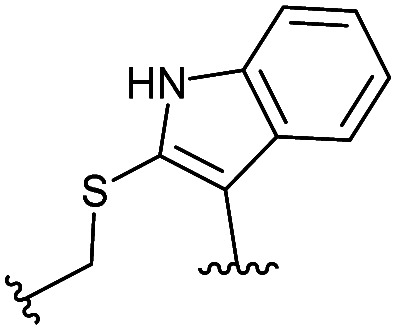	1.7 ± 0.3	0.101
P_+_	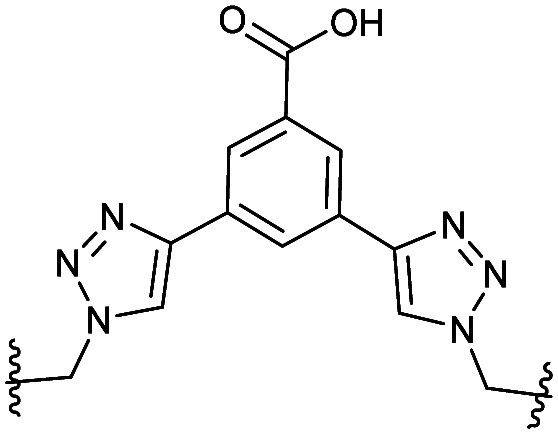	8.7 ± 4.2	0.057

Combination of the X-ray structures and IC_50_ values suggest that the homocysteine-cyclised peptide, P8C9, retains the binding conformation of the peptide more effectively than the cysteine-cyclised peptide, likely due to the flexibility provided by the extra carbon atom on the amino acid side chain (Fig. S6, ESI†). P8C9 shows both an improved potency and an improved LE compared to P_+_ and, as such, represents a promising starting point for the development of potent and cell permeable CK2 PPI inhibitors.

The binding of P8C9 to CK2α was further assessed using ITC (Fig. S7, ESI†) and its *K*_d_ was found to be 750 nM.

The serum stability of P8C9 was also analysed; it was found that P8C9 was fully stable in serum over 24 h compared to the linear peptide P8 which fully had degraded after 1 h (Fig. S8, ESI†). This was seen as a large improvement upon CAM7117 which is only 50% intact in serum after 24 h.^16^

X-ray crystallography was used to confirm the binding mode of P8C9. Comparison of the co-crystal structures of P8C9 and P_+_ in complex with CK2α indicates that the central binding sequence of the two peptides share the same binding conformation (Fig. 2c).

**Fig. 2:**
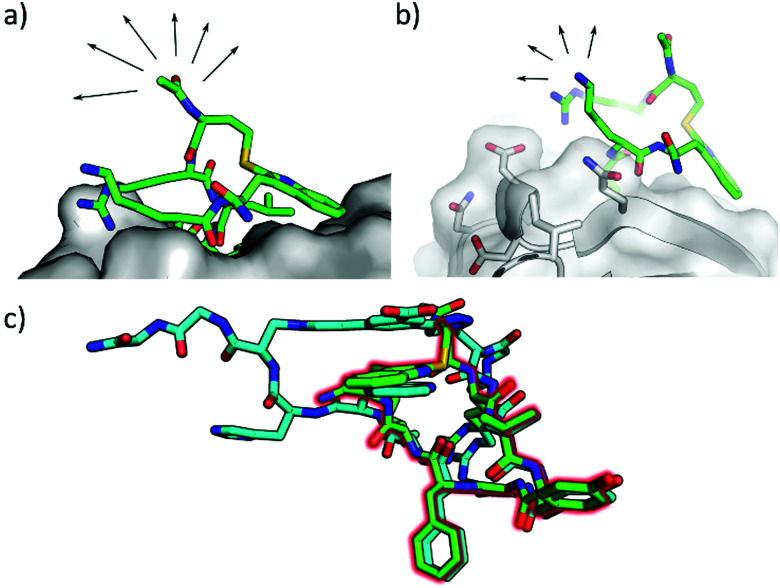
X-ray co-crystal structure of P8C9 in complex with CK2α (PDB: 6YZH). (a and b) The N-terminus and Lys residue of P8C9 are both solvent exposed making them good candidates as positions of functionalisation of the peptide. (c) Overlay of the crystal structures of P8C9 and P_+_ in complex with CK2α (PDB: 6Q4Q) highlighting that the central binding motif of the two peptides retain the same binding conformation.

Functionalisation of P8C9 would be desirable for the incorporation of cell penetrating tags, fluorescent dyes *etc*.; however, functionalisation through the staple is not facile due to the one-component stapling used. The crystal structure of P8C9 suggests that functionalisation of the peptide may be possible through the N-terminus or the Lys residue as both are solvent exposed (Fig. 2a and b). Indeed, functionalisation of the N-terminus of the peptide with a fatty acid and PEG chain only resulted in a minor decrease in binding affinity (*K*_d_ = 750 nM and 1.3 μM for P8C9 and FA-P8C9 respectively Fig. S7, ESI†), as did functionalisation of the Lys residue with a PEG chain§§Precipitation of P8C9[FA] occurred during ITC testing and thus, no *K*_d_ value could be obtained for the Lys-functionalised fatty acid derivative of P8C9. (*K*_d_ = 1.6 μM for P8C9[PEG], Fig. S7, ESI†) indicating that the addition of large groups to either the N-terminus or the Lys residue of P8C9 is likely to have little effect on its binding ability.

Finally, the effect of P8C9 was evaluated in HeLa cells to determine if the peptide was able to reduce cell viability. Unfortunately, P8C9 had no effect on cell viability (Fig. S10, ESI†). FA-P8C9 was evaluated in cells and found to reduce the cell viability. However, FA-PEG alone was observed to cause some toxicity, and thus FA-P8C9 was deemed to be causing partial non-specific toxicity (Fig. S11, ESI†).

Nevertheless, functionalisation of the N-terminus of P8C9 with the known cell penetrating peptide sequence TAT (GRKKRRQRRRPPQ)^21^ joined to the peptide through a di-Ahx spacer, TAT-P8C9, resulted in a large reduction in cell viability (Fig. 3), as did analogous functionalisation with a tri-Arg tag (R3-P8C9, Fig. 3). No effect on cell viability was observed for the cell penetrating tag-spacer conjugates alone (TAT-(Ahx)_2_ and R3-(Ahx)_2_, Fig. S13, ESI†). Thus, both TAT-P8C9 and R3-P8C9 represent promising CK2 modulating peptides for further investigation.

**Fig. 3:**
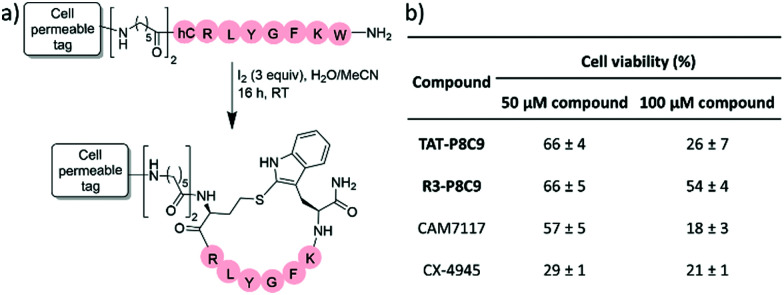
(a) Structure of cell penetrating P8C9 derivatives; CPP = TAT, TAT-P8C9; CPP = R3, R3-P8C9; hC = homocysteine. (b) Results of cell proliferation assay showing the percentage of viable cells remaining after treatment of HeLa cells with the specified compounds for 48 h. Average values are shown ±standard deviation.

In conclusion, the structure of CAM7117 has been optimised to reduce its molecular weight whilst retaining good activity and its synthesis simplified. Various shortened cyclic peptides were synthesised and biologically evaluated. P8C9 was found to exhibit similarly high potency to CAM7117 in enzymatic assays, as well as retaining the same binding conformation and displaying an increased stability compared to CAM7117. Functionalisation of P8C9 is possible through two positions on the peptide and incorporation of the cell penetrating peptides TAT and tri-Arg onto the N-terminus of the peptide provided peptides with good cellular toxicity. P8C9 is the smallest peptide CK2 binder to date displaying high serum stability. Its facile synthesis makes it a much more attractive chemical probe than CAM7117 and its small structure leaves room for optimisation of both efficacy and pharmacokinetic properties.

## Conflicts of interest

There are no conflicts to declare.

## Supplementary Material

CC-058-D2CC00707J-s001
