# Supplementary material for: Development of small cyclic peptides targeting the CK2α/β interface
Source: Chem Commun (Camb). 2022 Mar 21;58(30):4791–4. doi: 10.1039/d2cc00707j (PMC9004346; doi:10.1039/d2cc00707j)
Supplement: CC-058-D2CC00707J-s001 [file CC-058-D2CC00707J-s001.pdf]

Electronic Supplementary Material (ESI) for ChemComm.
This journal is © The Royal Society of Chemistry 2022

Supporting information

Development of small cyclic peptides targeting the CK2α/β interface

Eleanor L. Atkinson, Jessica Iegre, Claudio D'Amore, Paul D. Brear, Mauro Salvi, Marko Hyvönen and David R. Spring.

Table of contents

1.1 Figures, Tables and Schemes 3

1.2 Chemistry experimental 11

1.2.1 Experimental synthetic details 12

1.2.1.1 Small molecules 12

1.2.1.2 Peptides 24

1.3 Molecular modelling of cyclic peptides 48

1.4 Ligand efficiency 49

1.5 Biophysical experiments 49

1.5.1 Protein expression and purification 49

1.5.2 Fluorescence polarisation 49

1.5.3 Isothermal titration calorimetry 49

1.5.4 X-ray crystallography 50

1.6 Cellular biology 52

1.6.1 Cell culture 52

1.6.2 Cell viability assay 52

1.6.3 Serum stability test 52

1.7 References 53

1.1 Figures, Tables and Schemes

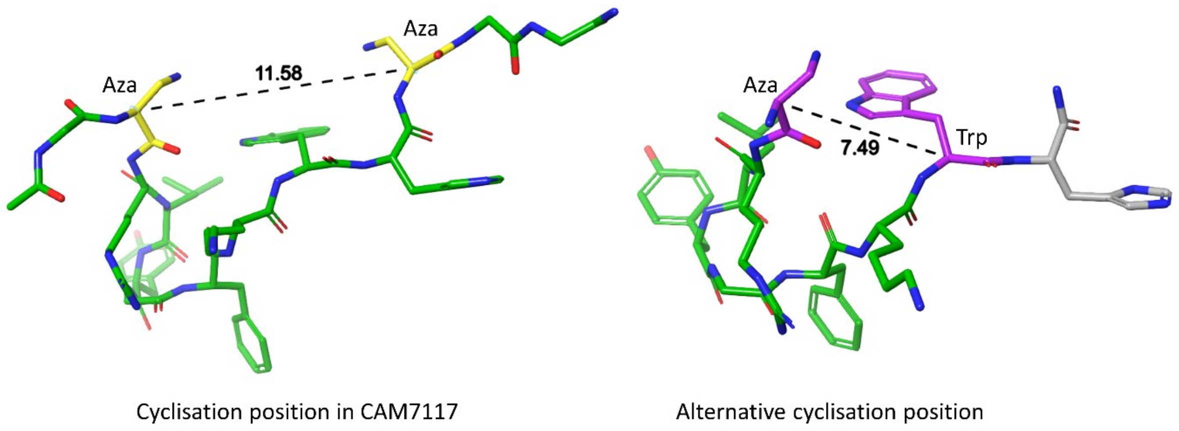

Fig. S1 Possible cyclisation positions of the central binding sequence of CAM7117. Left: Structure of CAM7117 with constraint removed. Cyclised residues are highlighted in yellow. Right: Alternative cyclisation position depicted on a shortened sequence of CAM7117 (constraint, terminal Gly and Aza-alanine residues removed). Residues able to be cyclised highlighted in purple. Distances shown are between the α-carbons of the cyclising residues and are in Å. Structures are derived from the crystal structure of CAM7117 bound to CK2α (PDB: 6Q38).

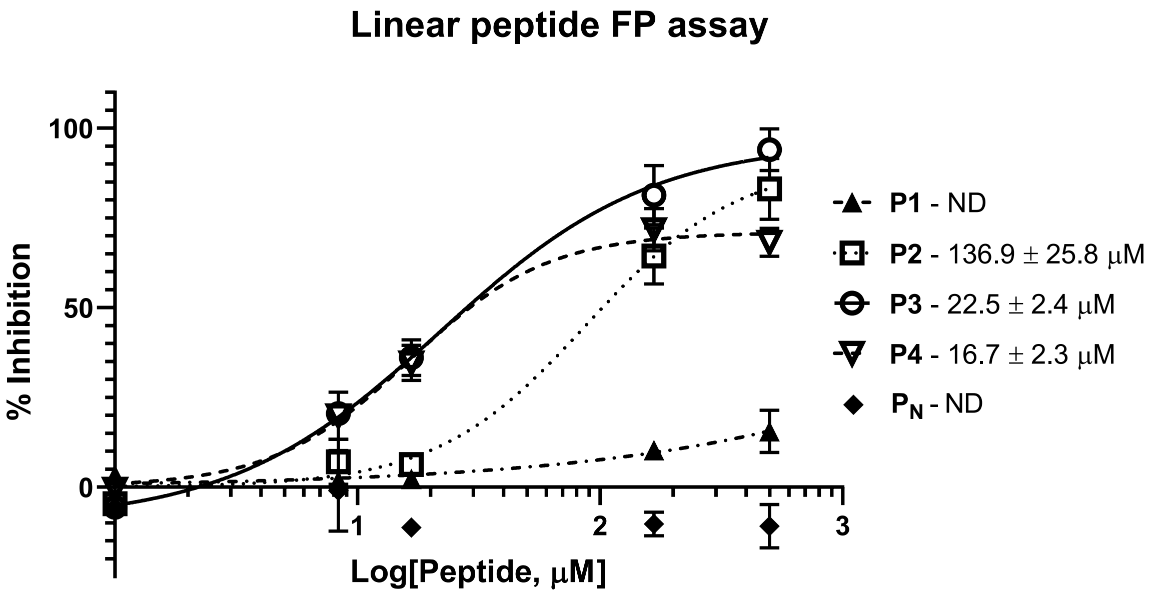

Fig. S2 Linear peptide FP assay results. The approximate IC_{50} values are shown next to the sequence of the peptide ± the standard error of the mean (SEM). ND = Non-determinable. IC_{50} curves are fitted to log(inhibitor) vs response standard curves using either 3 or 4 fitting parameters of GraphPad Prism software. All peptides have an amide at the C-terminus and are acetyl-capped at the N-terminus. All results were recorded in duplicate, on at least two separate occasions. P1 – RLYGFK, P2 – LYGFKW, P3 – RLYGFKW, P4 – RLYGFKWH and PN – HWKFGYLR.

Table S1 Chemical structures of CK2 peptides alongside molecular modelling predictions of the structures of the constraints after minimisation, keeping the peptide sequence in its binding conformation based on the crystal structure of CAM7117 in complex with CK2α (PDB: 6Q4Q).

Peptide structure 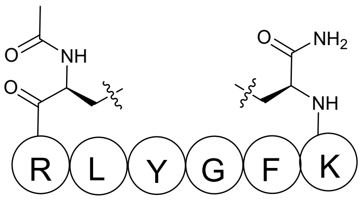

**Constraint**	**Structure of constraint**	**Modelling**
**P6C2**	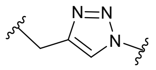	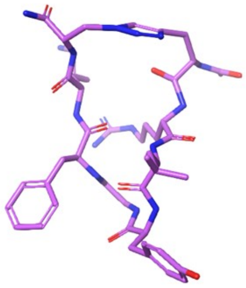
**P6C3**	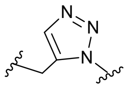	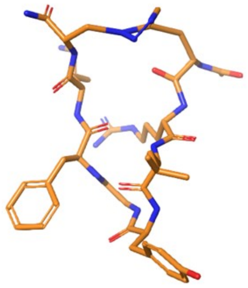
**P5C4**	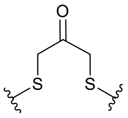	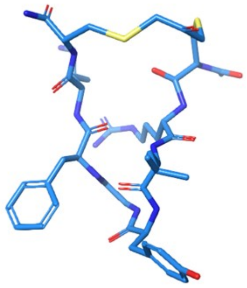
**P5C5**	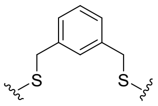	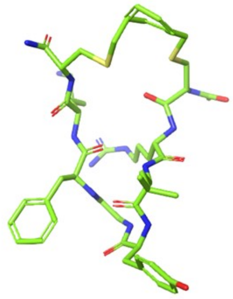

**P5C6**	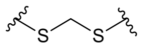	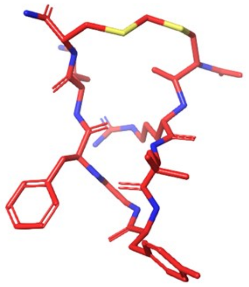
**P5C7**	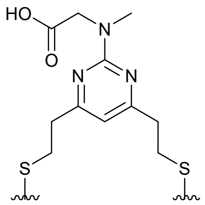	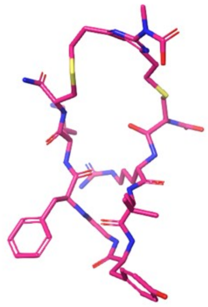
**P7C8**	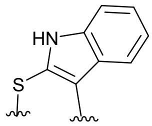	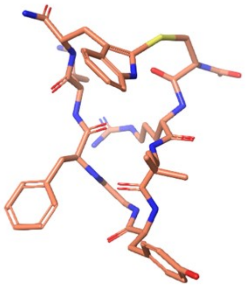
**P8C9**	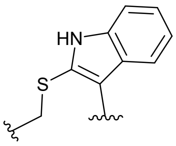	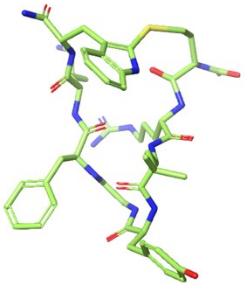

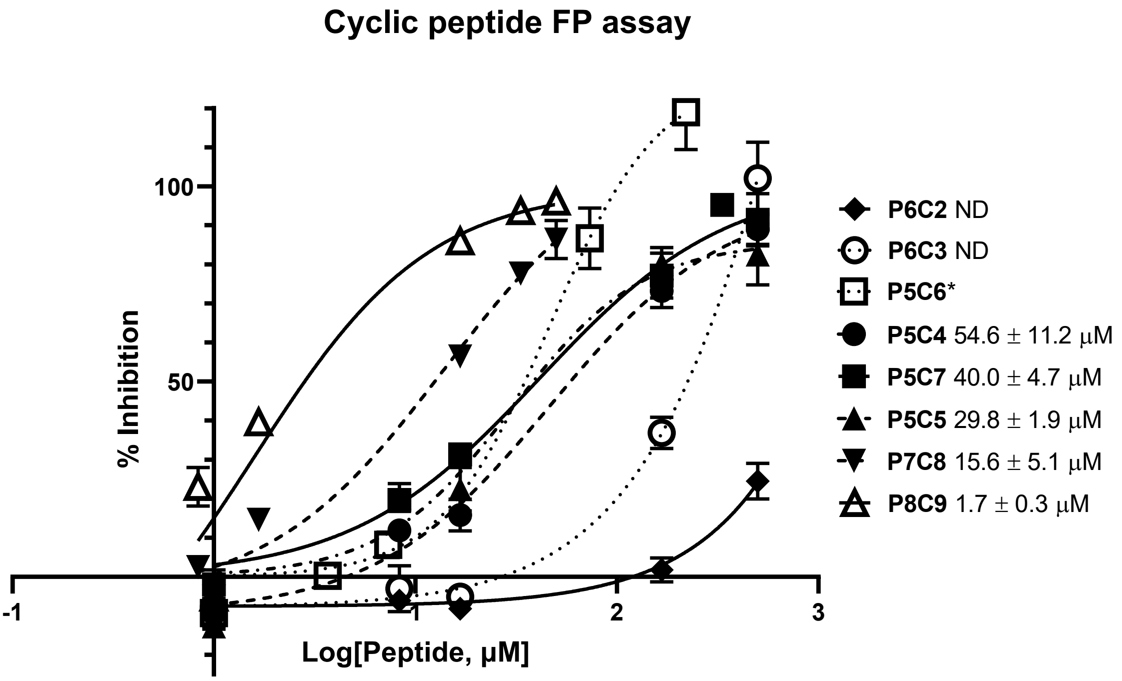

Fig. S3 Cyclic peptide FP results. IC_{50} values are shown in the legend in μM ± SEM. IC_{50} curves are fitted to log(inhibitor) vs response standard curves using either 3 or 4 fitting parameters of GraphPad Prism software. All results were recorded in duplicate, on at least two separate occasions. *IC_{50} of **P5C6** not determined due to large error and few data points. ND – not determinable.

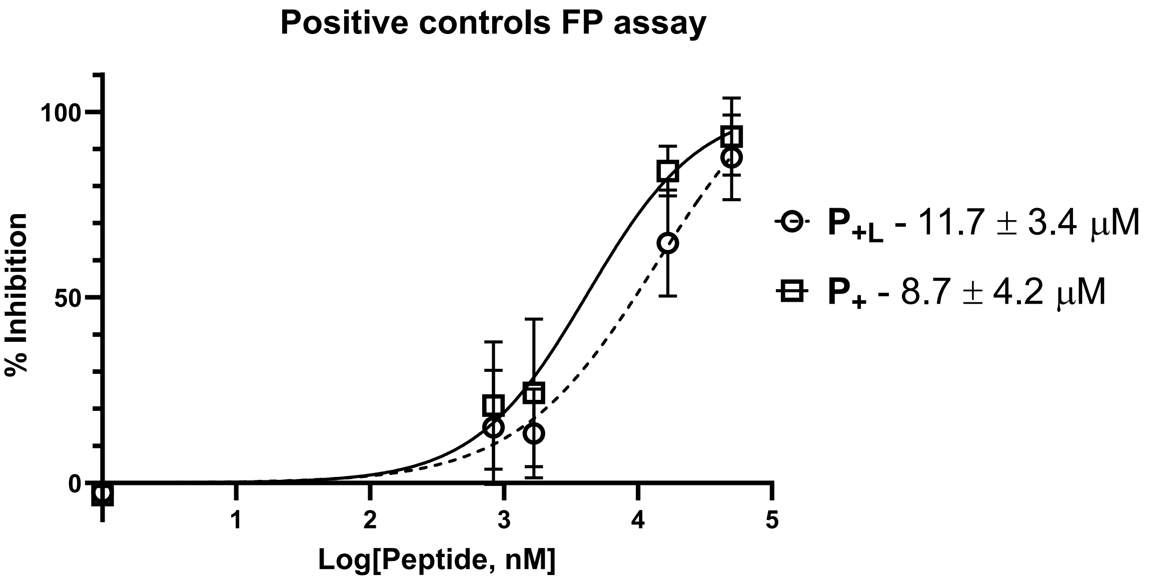

Fig. S4 Positive controls FP assay results. IC_{50} values are shown in the legend ± SEM. IC_{50} curves are fitted to log(inhibitor) vs response standard curves using either 3 or 4 fitting parameters of GraphPad Prism software. All results were recorded in duplicate, on at least two separate occasions.

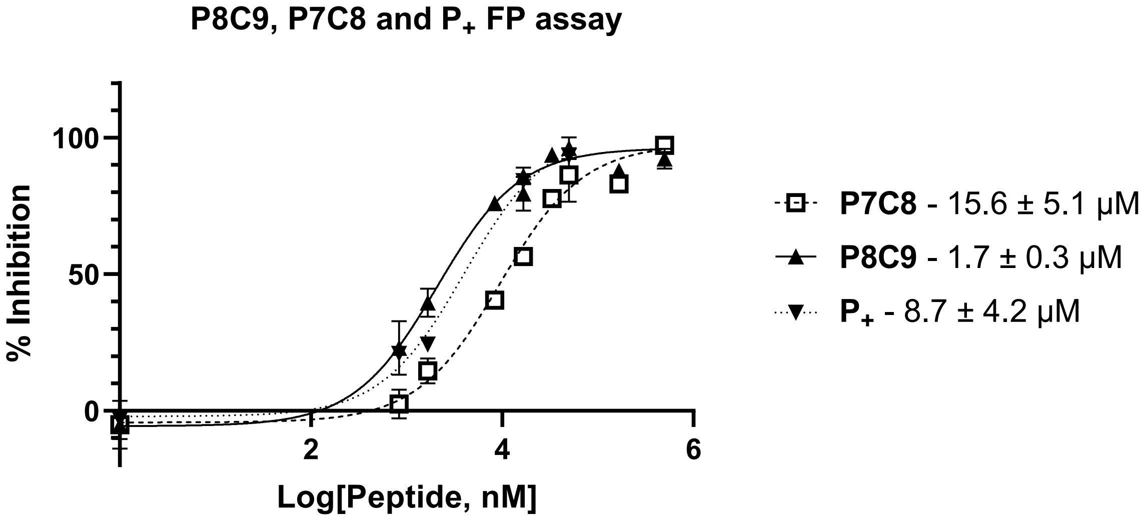

Fig. S5 P8C9, **P7C8** and **P+** FP assay over a larger concentration range. IC_{50} values are shown in the legend ± SEM. IC_{50} curves are fitted to log(inhibitor) *vs* response standard curves using either 3 or 4 fitting parameters of GraphPad Prism software. All results were recorded in duplicate, on at least two separate occasions.

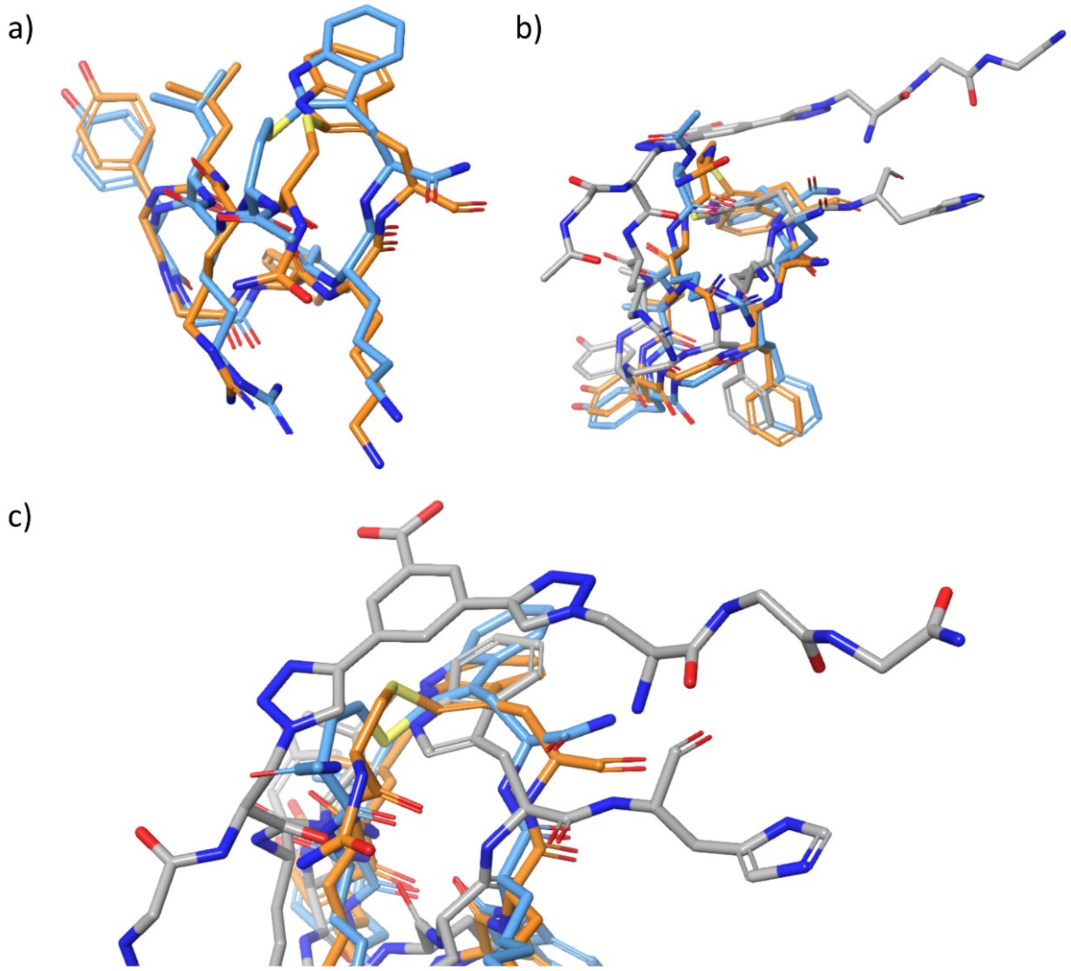

Fig. S6 Superimposed co-crystal structures of **P7C8** (orange, PDB: 7QUX), **P8C9** (faded azure, PDB: 6YZH) and **P+** (grey, PDB: 6Q4Q) in complex with CK2α. a) Overlay of the structures of **P7C8** and **P8C9** indicates that they bind in the same conformation, differing only in the positioning of the Trp ring of the staple. b) Overlay of the structures of **P7C8**, **P8C9** and **P+** confirms that the binding conformation of the central binding sequence of the peptides are the same; additionally, the reduced sizes of **P7C8** and **P8C9** are evident. c) A close up of the stapling positions of the peptides indicating that Trp residues of all three peptides occupy a very similar position.

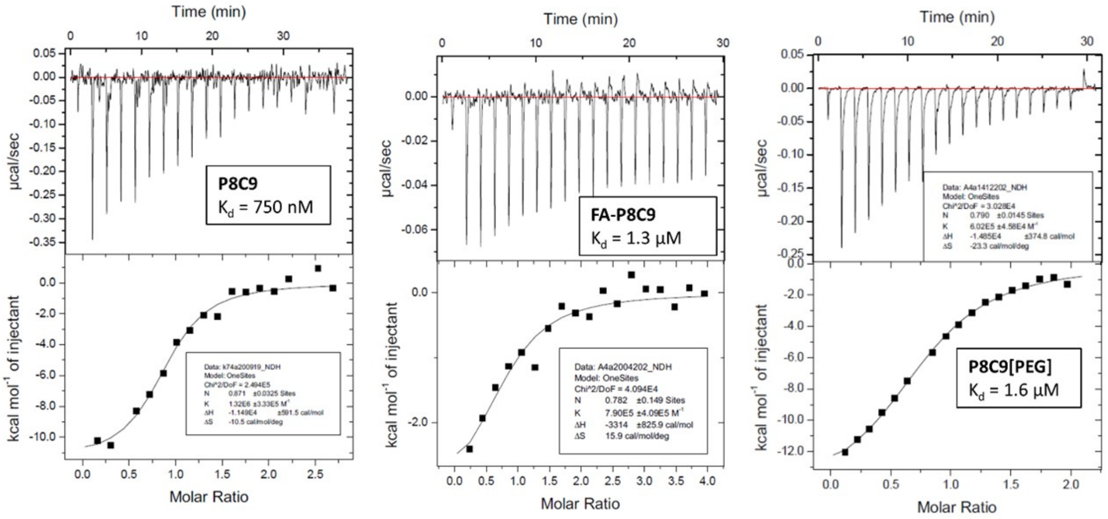

Fig. S7 ITC binding curves of **P8C9** binding to CK2α.

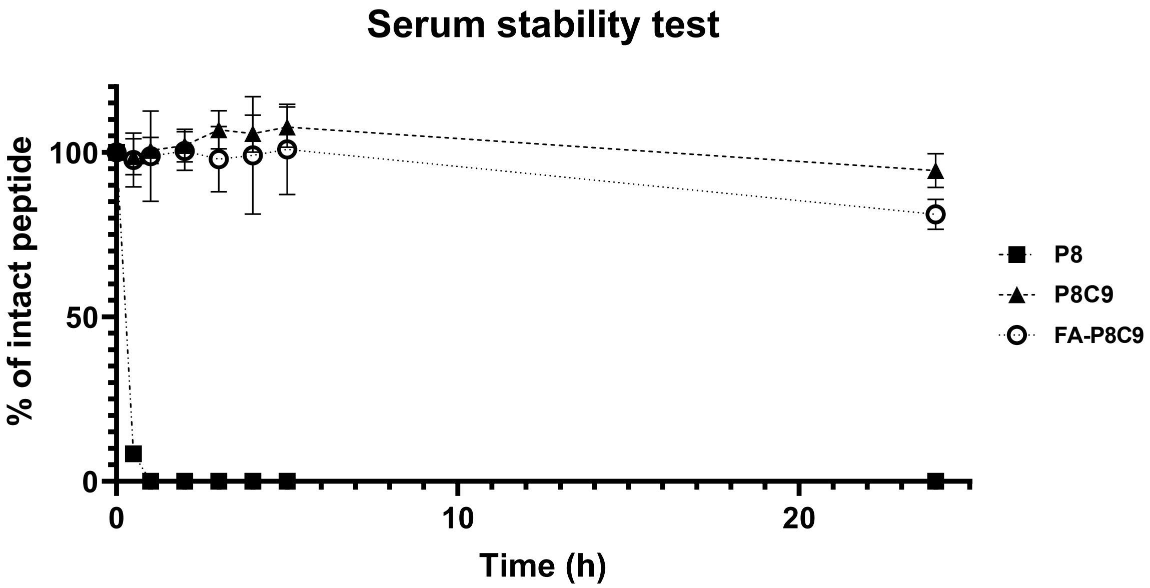

Fig. S8 Serum stability test of **P8**, **P8C9** and **FA-P8C9**.

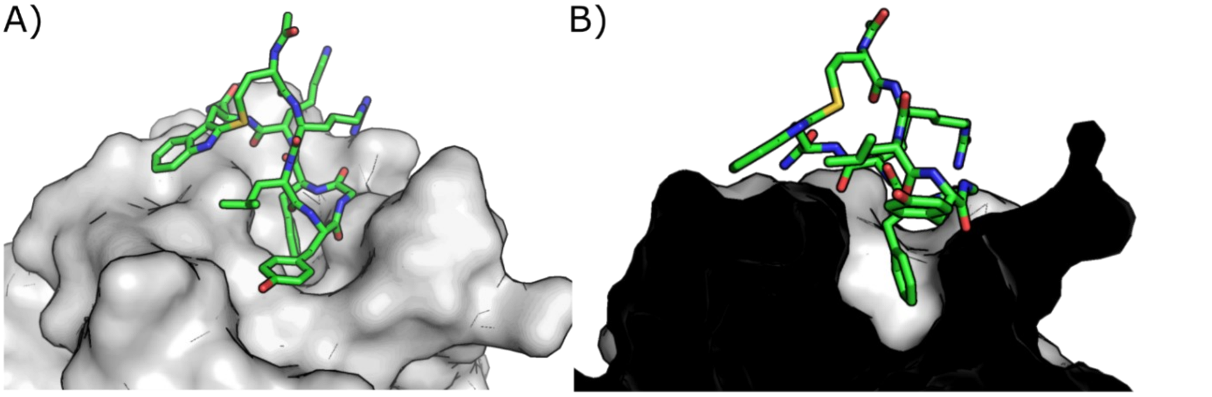

Fig. S9 X-ray co-crystal structure of **P8C9** in complex with CK2α (PDB: 6YZH). A) Binding conformation of **P8C9**. B) Phe residue of **P8C9** binds in the Phe-pocket of CK2α.

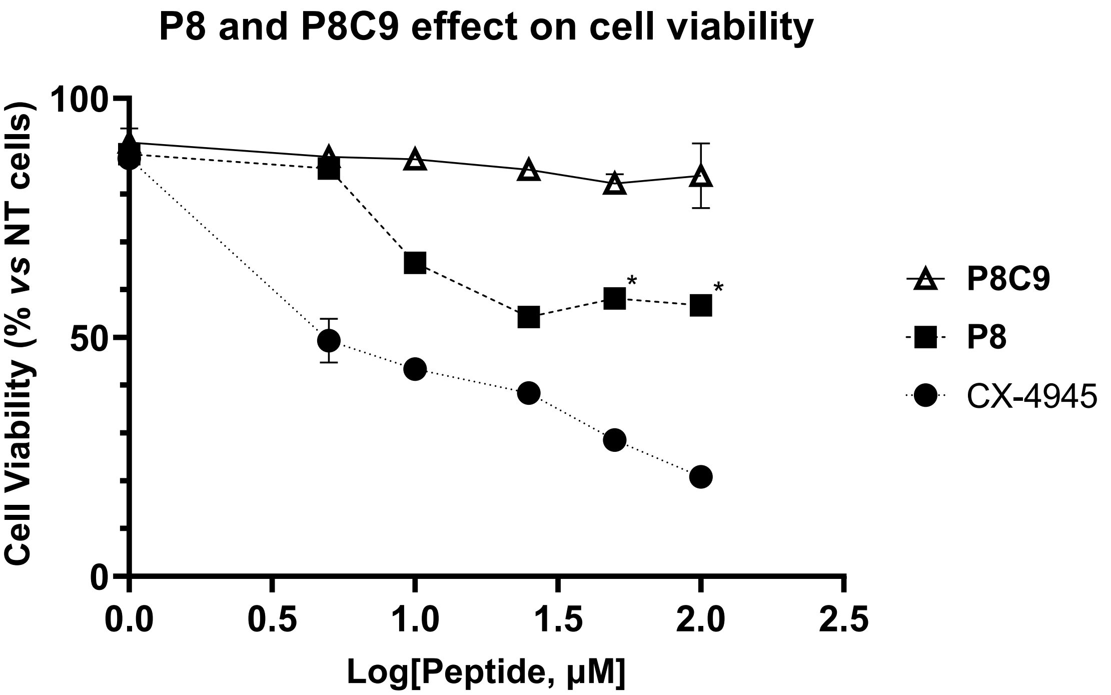

Fig. S10 P8C9 and **P8** MTT assay results. Cell viability is quantified as the percentage of viable cells remaining compared to the number of viable cells remaining for non-treated cells. CX-4945 is used as a positive control. The MTT assay was conducted in HeLa cells and the cells were incubated with the peptides for 48 h before analysis. *Peptide was seen to precipitate at these concentrations and thus, these results are unreliable.

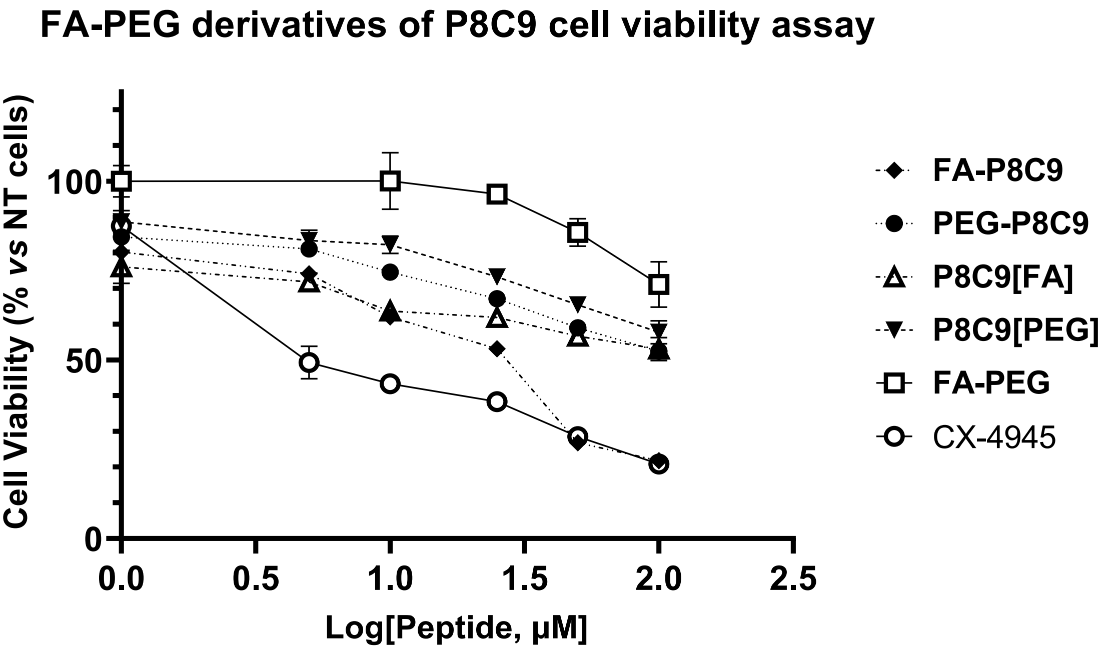

Fig. S11 FA-P8C9, **PEG-P8C9**, **P8C9[FA]**, **P8C9[PEG]** and **FA-PEG** MTT assay results. Cell viability is quantified as the percentage of viable cells remaining compared to the number of viable cells remaining for non-treated cells. CX-4945 is used as a positive control. The MTT assay was conducted in HeLa cells and the cells were incubated with the peptides for 48 h before analysis. Results were collected in quadruplicate on one or more occasions.

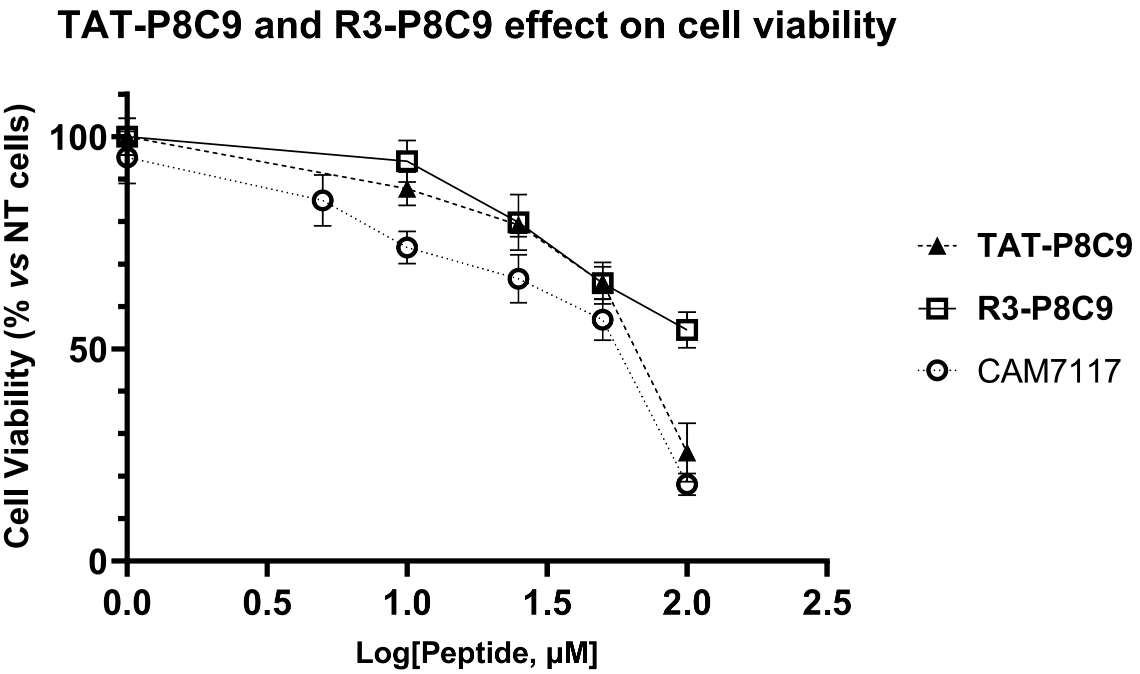

Fig. S12 TAT-P8C9 and **R3-P8C9** MTT assay results. Cell viability is quantified as the percentage of viable cells remaining compared to the number of viable cells remaining for non-treated cells. CAM7117 is used as a positive control. The MTT assay was conducted in HeLa cells and the cells were incubated with the peptides for 48 h before analysis. Results were collected in quadruplicate on two separate occassions.

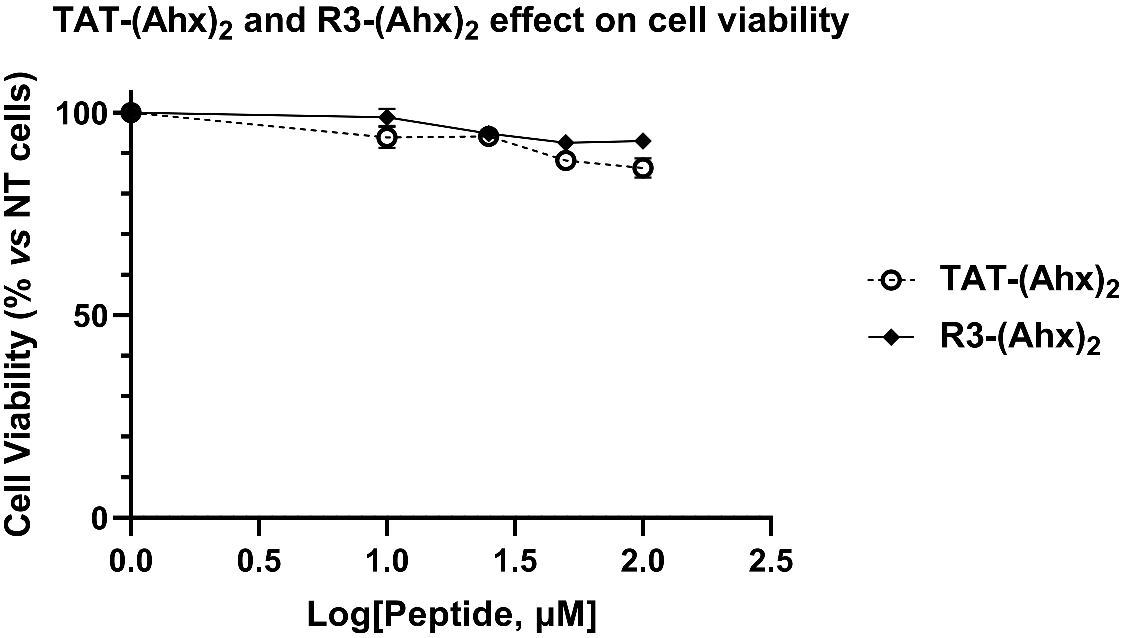

Fig. S13 TAT-(Ahx)$_2$ and **R3-(Ahx)$_2$** MTT assay results. Cell viability is quantified as the percentage of viable cells remaining compared to the number of viable cells remaining for non-treated cells. The MTT assay was conducted in HeLa cells and the cells were incubated with the peptides for 48 h before analysis. Results were collected in quadruplicate on two separate occasions.

1.2 Chemistry experimental

All experiments were carried out in oven-dried glassware under an atmosphere of N_2 using distilled solvents unless otherwise stated.

Reagents: Chemicals were purchased from commercial sources and used without further purification.

Yield: refers to chromatographically and spectroscopically pure compounds unless otherwise stated and are reported as follows: mass, moles, percentage

Temperature: Reaction temperatures of 0 °C were maintained using an ice-water bath; room temperature (rt) refers to 20-25 °C.

Flash chromatography: Analytical thin layer chromatography was carried out on SiO_2 Merck Kieselgel 60 F254 plates with visualisation either by ultraviolet light or by staining with potassium permanganate or ninhydrin dips made using standard procedures. Retention factors (R_f) are quoted to 0.01. Flash column chromatography was performed using silica gel 60 (230-400 mesh) under a positive pressure of N_2. Eluent systems are expressed in % v/v.

Nuclear Magnetic Resonance (NMR): ^{1}H and ^{13}C NMR spectra were recorded using an internal deuterium lock at ambient probe temperatures on the following instruments: Bruker Avance III 400 MHz HD Smart Probe Spectrometer, Bruker Avance III 400 MHz HD Spectrometer, Bruker 400 MHz QNP Cryoprobe Spectrometer, Bruker 500 MHz DCH Cryoprobe Spectrometer, Bruker Avance III 500 MHz HD Smart Probe Spectrometer. The following deuterated solvents were used: chloroform ($CDCl_3$) and dimethylsulfoxide (DMSO-d_6). ^{1}H-NMR chemical shifts (δ) are quoted in ppm to the nearest 0.01 ppm, relative to the residual non-deuterated solvent peak and coupling constants (*J*) are quoted to the nearest 0.1 Hertz (Hz). ^{13}C-NMR chemical shifts are quoted to the nearest 0.1 ppm, relative to the solvent peak and coupling constants are quoted to the nearest 0.1 Hz. Spectral data is reported as follows: chemical shift, integration, multiplicity (s, singlet; d, doublet; t, triplet; m, multiplet; br, broad; or as a combination of these e.g. br s), coupling constant(s) and assignment. The numbering system used in the assignments does not necessarily follow the IUPAC convention. Assignment of all spectra is supported by DEPT, COSY, HSQC and HMBC or by analogy to fully assigned spectra of closely related compounds.

Infrared spectroscopy (IR): Infrared spectra were recorded neat on a Perkin Elmer Spectrum One FT-IR spectrometer fitted with an Attenuated Total Reflectance (ATR) sampling accessory. Selected absorption maxima (ν_{max}) are quoted in wavenumbers (cm^{-1}) with the following abbreviations: w, weak; m, medium; s, strong; vs, very strong.

Liquid chromatography-mass spectrometry (LCMS): LCMS was carried out using a Waters ACQUITY H-Class UPLC with an ESCi Multi-Mode Ionisation Waters SQ Detector 2 spectrometer using MassLynx 4.1 software; EI refers to the electrospray ionisation technique; LC system: solvent A: 2 mM NH_4OAc in H_2O/MeCN (95:5); solvent B: MeCN; solvent C: 2% formic acid; column: ACQUITY UPLC® CSH C18 (2.1 mm × 50 mm, 1.7 µm, 130 Å) at 40 °C; gradient: 5 – 95% B with constant 5% C over 1 min at flow rate of 0.6 mL/min; Injection volume: 5 µL. Chromatographs were monitored by absorbance using diode array detection at a wavelength range of 190-600 nm, interval 1.2 nm.

Analytical HPLC: Chromatographs were obtained on an Agilent 1260 Infinity using a Supelcosil ABZ+PLUS column (150 mm x 4.6 mm, 3 µm) eluting with a linear gradient system (solvent A: 0.05% (v/v) TFA in water, solvent B: 0.05% (v/v) TFA in MeCN) over 15 min, unless otherwise stated, at a flow rate of 1 mL/min. HPLC was monitored by UV absorbance at 220 and 254 nm.

Preparative HPLC: Preparative HPLC was carried out on an Agilent 1260 Infinity using a Supelcosil ABZ+PLUS column (250 mm x 21.2 mm, 5 µm) eluting with a linear gradient system (solvent A: 0.1% (v/v) TFA in water, solvent B: 0.05% (v/v) TFA in MeCN) over 20 min at a flow rate of 20 mL/min. HPLC was monitored by UV absorbance at 220 and 254 nm.

Melting points: Melting points were measured using a Büchi melting point B545 apparatus and are uncorrected.

1.2.1 Experimental synthetic details

1.2.1.1 Small molecules

(S)-2-(9-Fluorenylmethyloxycarbonylamino)-3-azidopropanoic acid (Fmoc-Aza-OH) (1)

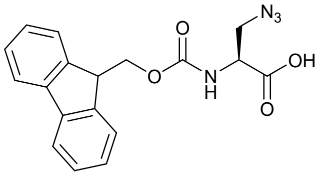

Fmoc-Asn-OH (4.0 g, 11.3 mmol, 1 equiv) was added to a solution of [bis(trifluoroacetoxy)iodo]benzene (5.4 g, 17.0 mmol, 1.5 equiv) in DMF/H_2O (2:1, 53.2 mL: 26.4 mL). After 15 min, pyridine (2.1 mL, 17.1 mmol, 1.5 equiv) was added and the mixture was stirred at rt for 16 h. The solvent was removed *in vacuo* and the oily residue was dissolved in H_2O (60 mL). Concentrated HCl (2 mL) was added and the solution washed with diethyl ether. The aqueous phase was adjusted to pH 6 with 2 M aqueous NaOH solution and the resulting precipitate was filtered, washed with H_2O, ice-cold EtOH and diethyl ether, and dried *in vacuo* to give Fmoc-Dap-OH as a beige powder (2.86 g, 8.8 mmol, 78%). The intermediate was used without further purification. Fmoc-Dap-

OH (2.86 g, 8.8 mmol, 1 equiv) was added to a mixture of H_2O (60 mL), MeOH (180 mL), and CH_2Cl_2 (120 mL). $CuSO_4{\cdot}5H_2O$ (14 mg, 0.06 mmol, 0.007 equiv) and 1-(azidosulfonyl)-1H-imidazol-3-ium hydrogen sulfate (**2**) (5.39 g, 21.1 mmol, 2.4 equiv) were added, the mixture adjusted to pH 9 with saturated K_2CO_3 and stirred for 18 h. The mixture was then diluted with CH_2Cl_2 (120 mL), and the aqueous phase isolated. The organic phase was extracted with saturated aqueous $NaHCO_3$ (2×200 mL) and the organic layers discarded. The aqueous extract was then washed with Et_2O (2×200 mL), the aqueous extract acidified to pH 2 with conc. HCl and extracted again with Et_2O (3×240 mL). The organic extracts were dried over anhydrous $MgSO_4$ and the solvent removed *in vacuo* to yield the title compound as a beige, amorphous solid (1.33 g, 3.77 mmol, 37%).

R$_f$ = 0.09 (10% MeOH/DCM); **mp** 121-123 °C (lit. 123-125 °C)[1]; **$[\alpha]_D^{25}$** = -7.0 ° (DMF, concentration = 1 g/100 mL) (lit. **$[\alpha]_D^{25}$** = -10.3 ° (DMF, concentration = 1 g/100 mL));[2] **ν_{max}** (cm^{-1}) 2106 (m, N=N=N), 1705 (s, C=O), 1687 (s, C=O), 1536 (s, C=C); **δ_H** (400 MHz, $CDCl_3$) 3.78-3.86 (2H, m), 4.24 (1H, t, *J*=6.9 Hz), 4.40-4.48 (2H, m), 4.58-4.60 (1H, m), 5.64 (1H, d, *J*=7.4 Hz), 7.32 (2H, t, *J*=7.4 Hz), 7.41 (2H, t, *J*=7.4 Hz), 7.59 (2H, d, *J*=6.9 Hz), 7.77 (2H, d, *J*=7.4 Hz); **δ_C** (100 MHz, $CDCl_3$) 47.2, 52.4, 53.7, 67.6, 120.2, 125.2, 127.3, 128.0, 141.46, 143.6, 156.0, 173.3; **LCMS** (ESI^-): *m/z* found $[M-H]^-$ 352.4.

Data in accordance with literature.[1, 2]

1-(azidosulfonyl)-1H-imidazol-3-ium hydrogen sulfate (2)

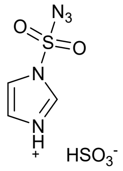

Sulfuryl chloride (16.1 mL, 200 mmol, 1 equiv) was added dropwise to an ice-cold suspension of NaN_3 (13.0 g, 200 mmol, 1 equiv) in CH_3CN (200 mL) and the mixture stirred for 16 h. Imidazole (25.9 g, 380 mmol, 1.9 equiv) was added and the pink mixture stirred at 0 °C for 5 h. The mixture was then diluted with ethyl acetate (400 mL) and H_2O (400 mL). The organic fraction was isolated and washed with H_2O (400 mL) and saturated aqueous $NaHCO_3$ (2 x 200 mL), dried over anhydrous $MgSO_4$ and the solvent reduced to 200 mL *in vacuo*. A solution of conc. H_2SO_4 (11 mL, 200 mmol, 1 equiv) in ethyl acetate (100 mL) was added to the ice-cold reaction mixture over 30 min. The reaction mixture was warmed to rt and stirred for 16 h. The precipitate was filtered off, washed with ethyl acetate (3 x 60 mL) and dried *in vacuo* to yield the title compound as a white powder (25.3 g, 93 mmol, 47%).

R$_f$ = 0.15 (20% EtOAc/petroleum ether 40:60); **mp** 103-107 °C (lit. 102-105 °C);[3] **ν_{max}** (cm^{-1}) 2178 (m, N=N=N), 1586 (m, C=C), 1430 (s, S=O); **δ_H** (400 MHz, DMSO-d_6) 7.52 (1H, s), 8.04 (1H, s), 9.11 (1H, s), 11.94-12.08 (3H, br s, expected 1H), 14.24 (1H, br s); **δ_C** (100 MHz, DMSO-d_6) 120.1, 127.6, 138.7. Extra

peaks at 7.57 and 8.95 ppm in the ^{1}H NMR spectrum, and at 111.9 and 134.9 in the ^{13}C NMR sprectrum arise from the compound partially decomposing in DMSO-d_6 to form 1,3H-imidazol-1-ium salts; **LCMS** (ESI$^+$) *m/z* found $[M+H]^+$ 174.3.

Data in accordance with literature.[3]

Methyl 3,5-bis((trimethylsilyl)ethynyl)benzoate (3)

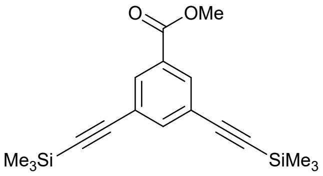

Trimethylsilylacetylene (6 mL, 42.2 mmol, 13.6 equiv) was added to a stirring mixture of methyl 3,5-dibromobenzoate (900 mg, 3.10 mmol, 1 equiv), $Pd_2(dba)_3$ (54 mg, 0.06 mmol, 2 mol%) and triphenylphosphine (77 mg, 0.29 mmol, 9 mol%) in dry triethylamine (15 mL). The reaction mixture was refluxed for 16 h under N_2. The solvent was removed *in vacuo*, the residue diluted with EtOAc and washed with H_2O. The organic phase was dried over anhydrous $MgSO_4$ and the crude residue purified by flash column chromatography on silica gel eluting with 0-5% EtOAc in petroleum ether 40-60 to afford the title compound as a yellow solid (954 mg, 2.90 mmol, 94%).

R_f = 0.71 (20% EtOAc/petroleum ether 40:60); **mp** 76-78 °C (lit. 73-75 °C);[4] **ν_{max}** (cm^{-1}) 2955 (w, C≡CH), 1730 (s, C=O), 1588 (w, C=C), 1247 (s, Si-C); **δ_H** (400 MHz, DMSO-d_6) 0.27 (18H, s), 3.94 (3H, s), 7.75 (1H, t, *J*=1.5 Hz), 8.06 (2H, d, *J*=1.5 Hz); **δ_C** (100 MHz, DMSO-d_6) 0.0, 52.6, 96.3, 103.1, 124.1, 130.7, 132.8, 139.2, 165.9; **LCMS** (ESI$^+$) *m/z* found $[M-OMe]^+$ 297.4.

Data in accordance with literature.[4]

3,5-Diethynylbenzoic acid (4)

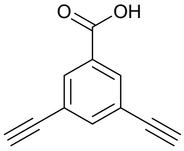

Aqueous 6M KOH (4.31 mL, 25 mmol, 10 equiv) was added to a stirred solution of methyl 3,5-bis((trimethylsilyl)ethynyl)benzoate (850 mg, 2.59 mmol, 1 equiv) in MeOH (5 mL). The mixture was stirred at rt for 18 h. MeOH was removed under a stream of N_2, the residue partitioned between EtOAc and H_2O and the aqueous phase acidified to pH 4 with 6M aqueous HCl. The aqueous phase was extracted with EtOAc (3x5 mL) and the solvent removed *in vacuo* to afford the title compound as an orange solid (403 mg, 2.37 mmol, 92%).

R$_f$ = 0.32 (10% MeOH/DCM); **mp** 168 °C decomposed (lit. 173 °C decomposed);[5] **ν$_{max}$** (cm^{-1}) 3286 (m, C≡CH), 1683 (vs, C=O), 1588 (s, C=C); **δ$_H$** (400 MHz, DMSO-d$_6$) 4.37 (2H, s), 7.79 (1H, t, *J*=1.6 Hz), 7.94 (2H, d, *J*=1.6 Hz); **δ$_C$** (100 MHz, DMSO-d$_6$) 81.9, 83.1, 123.3, 132.5, 132.9, 138.7, 166.1; **LCMS** (ESI$^-$) *m/z* found [M-H]$^-$ 169.3.

Data in accordance with literature.[5]

***N*-(2-(2-(2-amino-2-oxoethoxy)ethoxy)ethyl)dodecanamide (5)**

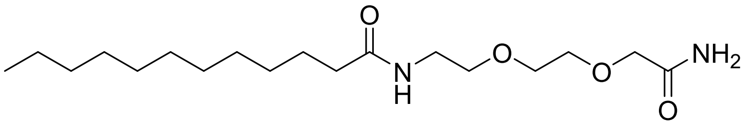

N-(2-(2-(2-amino-2-oxoethoxy)ethoxy)ethyl)dodecanamide was synthesised using automated SPPS as outlined in section 1.2.1.2, using rink amide resin (0.1 mmol scale), {2-[2-(Fmoc-amino)ethoxy]ethoxy}acetic acid and dodecanoic acid. The compound was cleaved from the resin using TFA/TIPS/CH_2Cl_2/H_2O and the crude product purified by flash column chromatography (10% MeOH/CH_2Cl_2) to yield the product as a white amorphous solid (30.8 mg, 0.089 mmol, 89.4% yield).

R$_f$ = 0.44 (10% MeOH/CH_2Cl_2); **mp** 92 – 94 °C; **ν$_{max}$** (cm^{-1}) 3305 (m, N-H) 1662 (s, C=O) 1636 (vs, C=O) 1553 (m, N-H) 1109 (s, C-O); **δ$_H$** (400 MHz, $CDCl_3$) 0.87 (3H, t, *J* = 7.0 Hz) 1.25 – 1.29 (20 H, bm) 1.60 – 1.63 (2H, bm) 2.18 (2H, t, *J* = 7.6 Hz) 3.46 – 3.57 (4H, bms) 3.63 – 3.72 (4H, bms) 4.01 (2H, s) 5.67 (1H, bs) 5.93 (1H, bs) 6.84 (1H, bs); **δ$_C$** (100 MHz, $CDCl_3$) 14.1, 22.7, 25.7, 29.3 – 29.6, 31.9, 36.7, 39.1, 70.1 – 71.0, 172.9, 173.5; **LCMS** (ESI$^+$) *m/z* found [M+H]$^+$ 345.2; **HRMS** (ESI$^+$) *m/z* [M+Na]$^+$ calculated for $C_{18}H_{36}N_2O_4$: 367.2567; found: 367.2570 (Δ = 0.7 ppm).

Ethyl *N*-(4,6-dichloropyrimidin-2-yl)-*N*-methylglycinate (6)

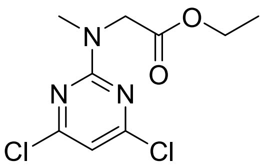

To a solution of 2,4,6-trichloropyrimidine (1.00 g, 5.45 mmol, 1 equiv) in acetone (6 mL) at 0 °C was added sarcosine ethyl hydrochloride (1.01 g, 6.54 mmol, 1.2 equiv) followed by dropwise addition of triethylamine (1.90 mL, 13.6 mmol, 2.5 equiv) and the reaction mixture was stirred at 0 °C for 2 h and then at rt for 12 h. Upon completion, the solvent was removed *in vacuo* then redissolved in H_2O (20 mL) and extracted with CH_2Cl_2 (4 × 20 mL). The combined organic fractions were dried ($MgSO_4$), concentrated *in vacuo* and the crude residue purified by FCC (2-20% EtOAc/PE) to yield the product (200 mg, 0.80 mmol, 14%) as a clear oil.

R_f = 0.30 (10% EtOAc/PE 40-60); **δ_H** (400 MHz, $CDCl_3$) 6.61 (s, 1H), 4.35 (s, 2H), 4.23 (q, 2H, *J* = 7.2 Hz), 3.58 (s, 3H), 1.30 (t, 3H, *J* = 7.2 Hz) ppm; **HRMS** (ESI^+) m/z found $[M+H]^+$ 264.0299, $C_9H_{12}Cl_2N_3O_2^+$ required 264.0307.

Data in accordance with literature procedure.[6]

Ethyl *N*-(4,6-divinylpyrimidin-2-yl)-*N*-methylglycinate (7)

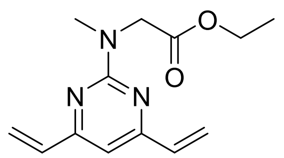

6 (180 mg, 0.638 mmol, 1 equiv), potassium vinyltrifluoroborate (581 mg, 4.34 mmol, 6.8 equiv), $Pd(dppf)Cl_2$▪CH_2Cl_2 (73 mg, 0.10 mmol, 0.15 equiv) and K_2CO_3 (660 mg, 4.08 mmol, 6.4 equiv) in THF/H_2O (10:1, 4.8 mL) were refluxed at 70 °C for 16 h. Upon completion, the reaction mixture was filtered through Celite®, washed with EtOAc and the solvent removed *in vacuo*. The resulting residue was purified by FCC (0-4% EtOAc/PE 40-60) to yield **7** (150 mg, 0.60 mmol, 95%) as an off-white solid.[a]

R_f = 0.30 (10% EtOAc/PE 40-60); **δ_H** (400 MHz, $CDCl_3$) 6.64-6.57 (m, 2H), 6.51 (s, 1H), 6.40 (d, 2H, J = 17.2 Hz), 5.55 (dd, 2H, *J* = 10.5, 1.0 Hz), 4.38 (s, 2H), 4.19 (q, 2H, *J* = 7.1 Hz), 3.34 (s, 3H), 1.30-1.24 (m, 3H) ppm; **HRMS** (ESI^+) m/z found $[M+H]^+$ 248.1397, $C_{13}H_{18}N_3O_2^+$ required 248.1399.

Data in accordance with literature procedure.[6]

***N*-(4,6-Divinylpyrimidin-2-yl)-*N*-methylglycine (8)**

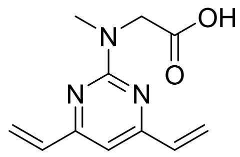

To a solution of **7** (160 mg, 0.65 mmol, 1 equiv) in THF/H_2O (1:1, 6 mL) was added $LiOH$▪H_2O (90 mg, 2.14 mmol, 3.3 equiv) and the reaction mixture stirred at rt for 18 h. Upon completion, the mixture was diluted with H_2O (10 mL) and washed with Et_2O (10 mL). The aqueous phase was neutralized with 1M HCl and extracted with CH_2Cl_2 (4 × 20 mL). The combined organic fractions were dried ($MgSO_4$) and concentrated *in vacuo*. The crude residue was triturated with PE 40-60 to yield **8** (100 mg, 0.45 mmol, 70%) as a pale yellow solid.

[a] The pure product was dissolved straightaway in THF/H_2O (2 mL) for the next step.

δ_H (400 MHz, CDCl3) 6.67-6.60 (m, 3H), 6.42 (dd, 2H, *J* = 17.3, 0.8 Hz), 5.66 (dd, 2H, *J* = 10.6, 1.2 Hz), 4.32 (s, 2H), 3.40 (s, 3H) ppm; **LCMS** (ESI^+) 220.1 $[M+H]^+$.

Data in accordance with literature procedure.[6]

1.2.1.1.1 ^{1}H and ^{13}C NMR

1

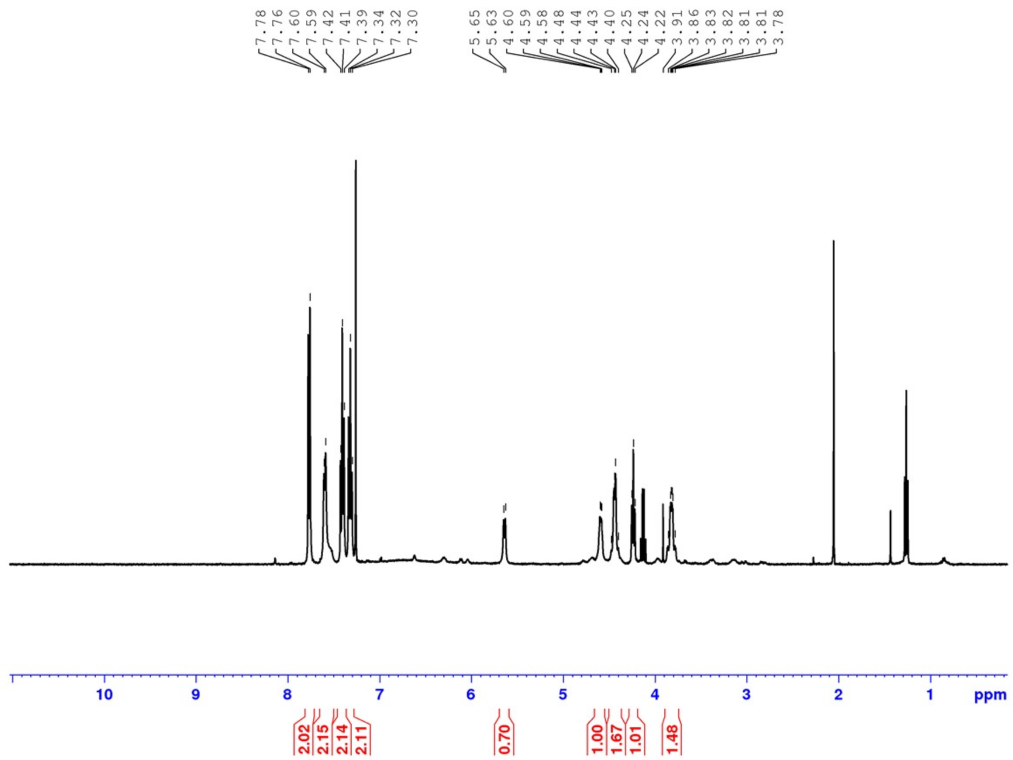

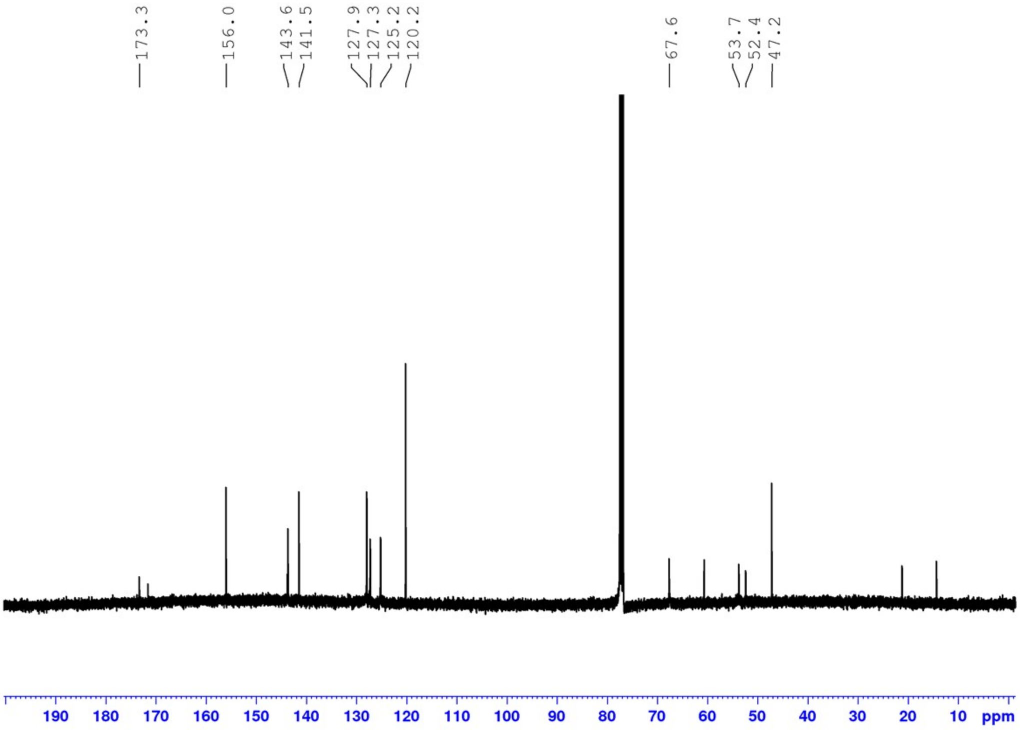

2

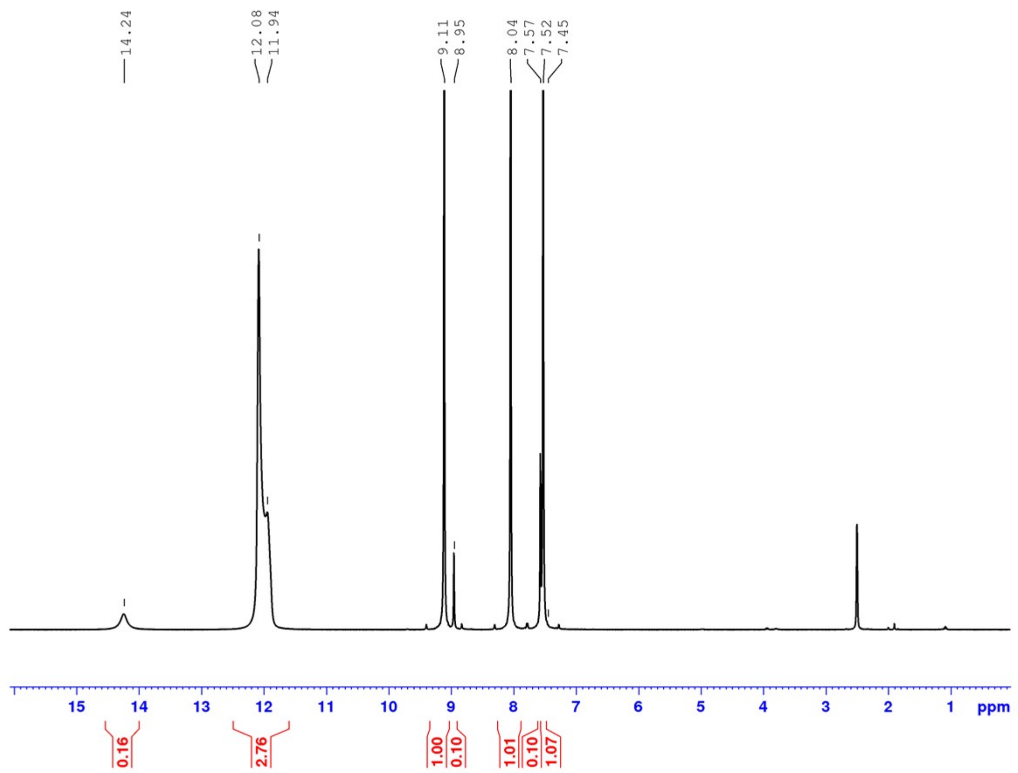
14.24
12.08
11.94
9.11
8.95
8.04
7.57
7.52
7.45
15
14
13
12
11
10
9
8
7
6
5
4
3
2
1
ppm
0.16
2.76
1.00
0.10
1.01
0.10
1.07

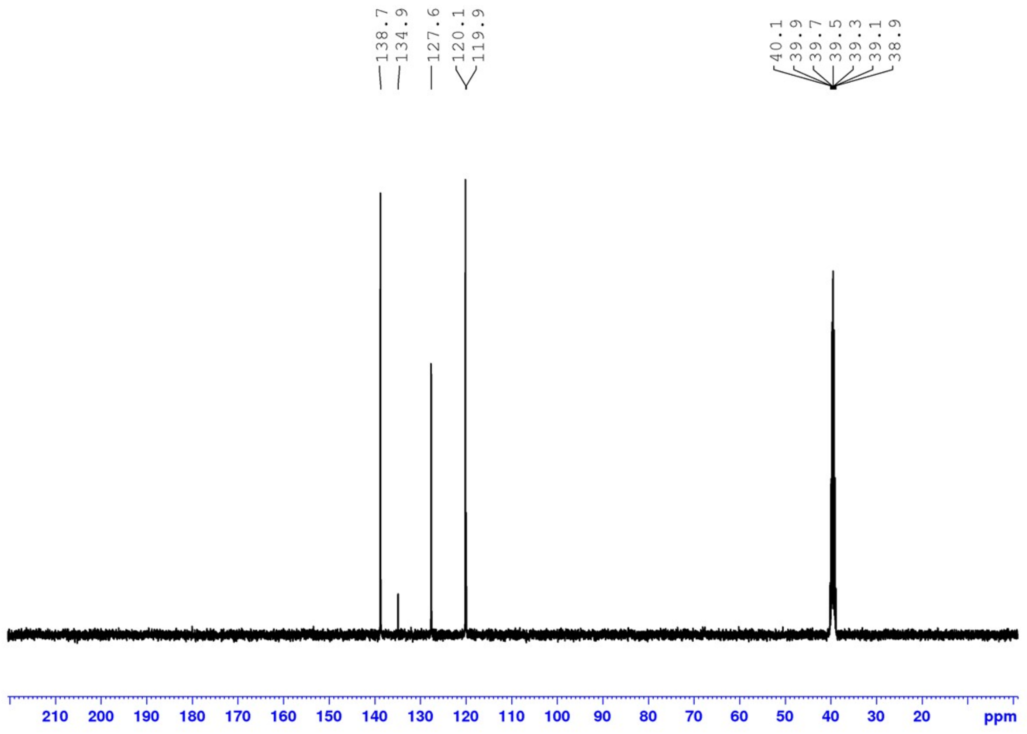
138.7
134.9
127.6
120.1
119.9
40.1
39.9
39.7
39.5
39.3
39.1
38.9
210
200
190
180
170
160
150
140
130
120
110
100
90
80
70
60
50
40
30
20
ppm

3

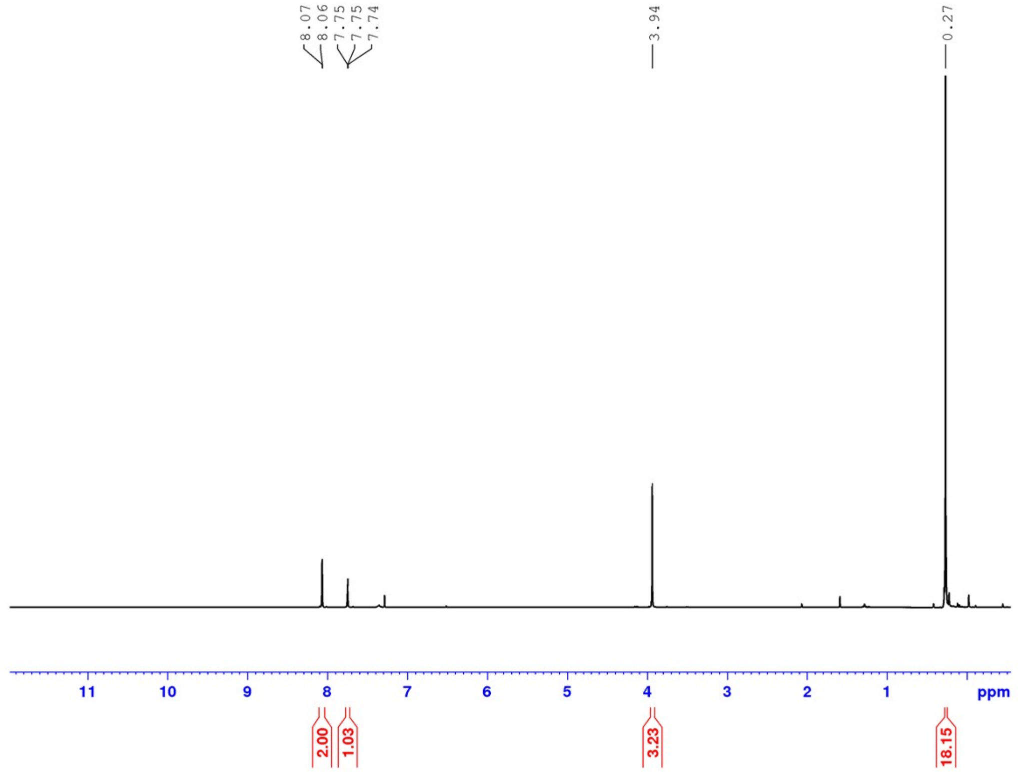
8.07
8.06
7.75
7.75
7.74
3.94
0.27
11 10 9 8 7 6 5 4 3 2 1 ppm
2.00
1.03
3.23
18.15

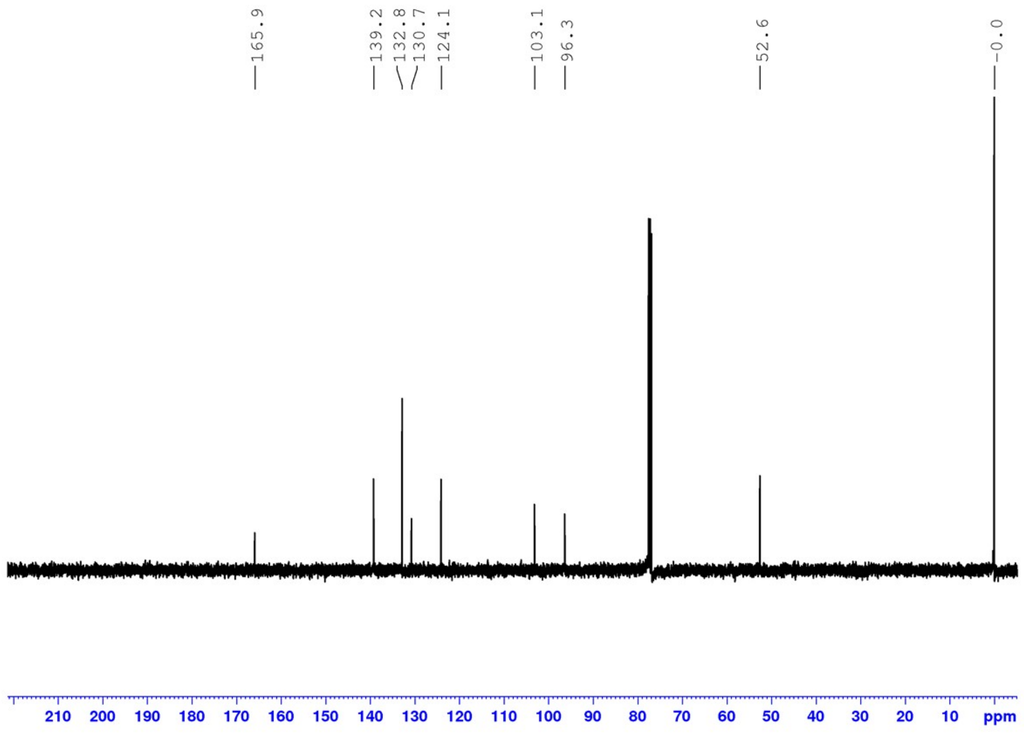
165.9
139.2
132.8
130.7
124.1
103.1
96.3
52.6
-0.0
210 200 190 180 170 160 150 140 130 120 110 100 90 80 70 60 50 40 30 20 10 ppm

4

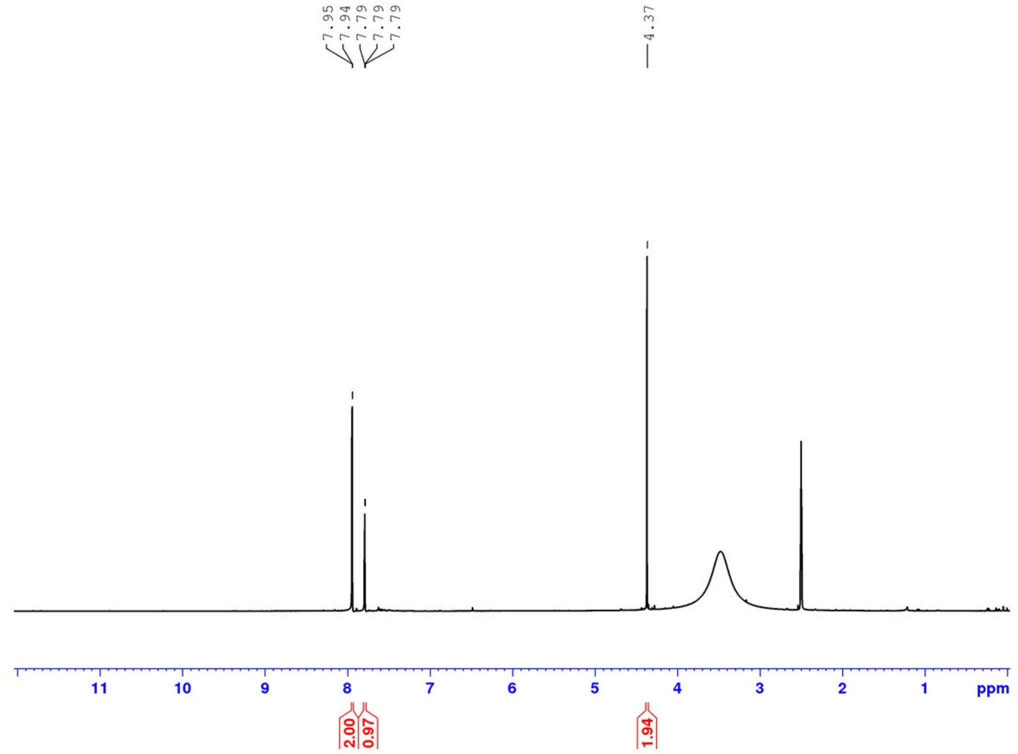
7.95
7.94
7.79
7.79
7.79
4.37
11 10 9 8 7 6 5 4 3 2 1 ppm
2.00
0.97
1.94

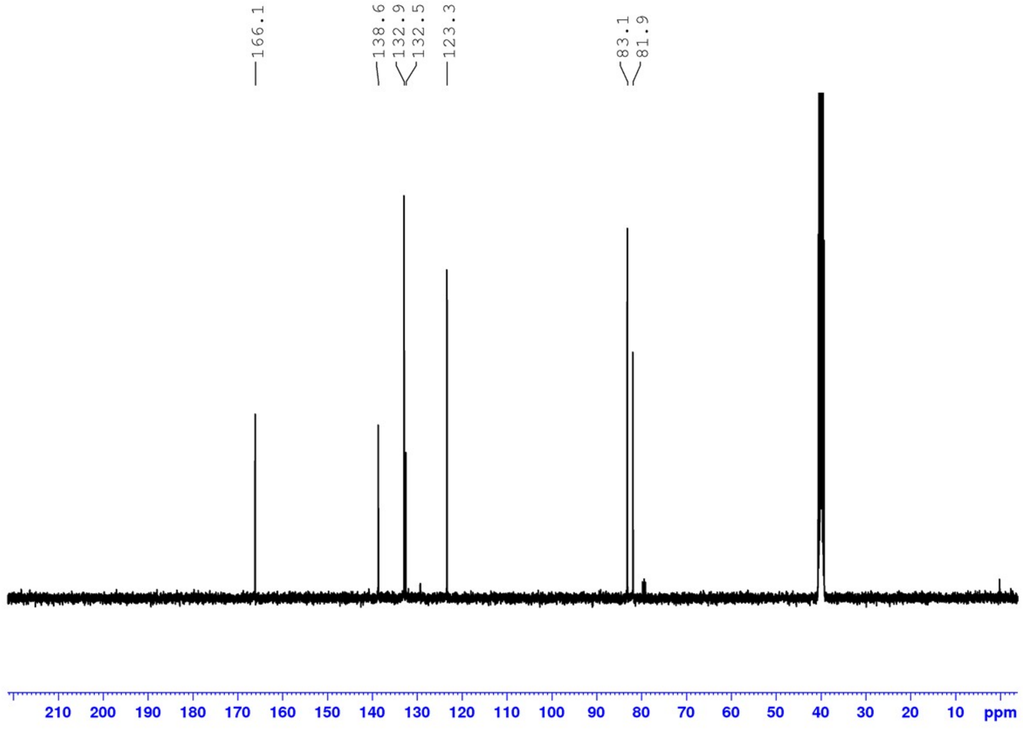
166.1
138.6
132.9
132.5
123.3
83.1
81.9
210 200 190 180 170 160 150 140 130 120 110 100 90 80 70 60 50 40 30 20 10 ppm

5

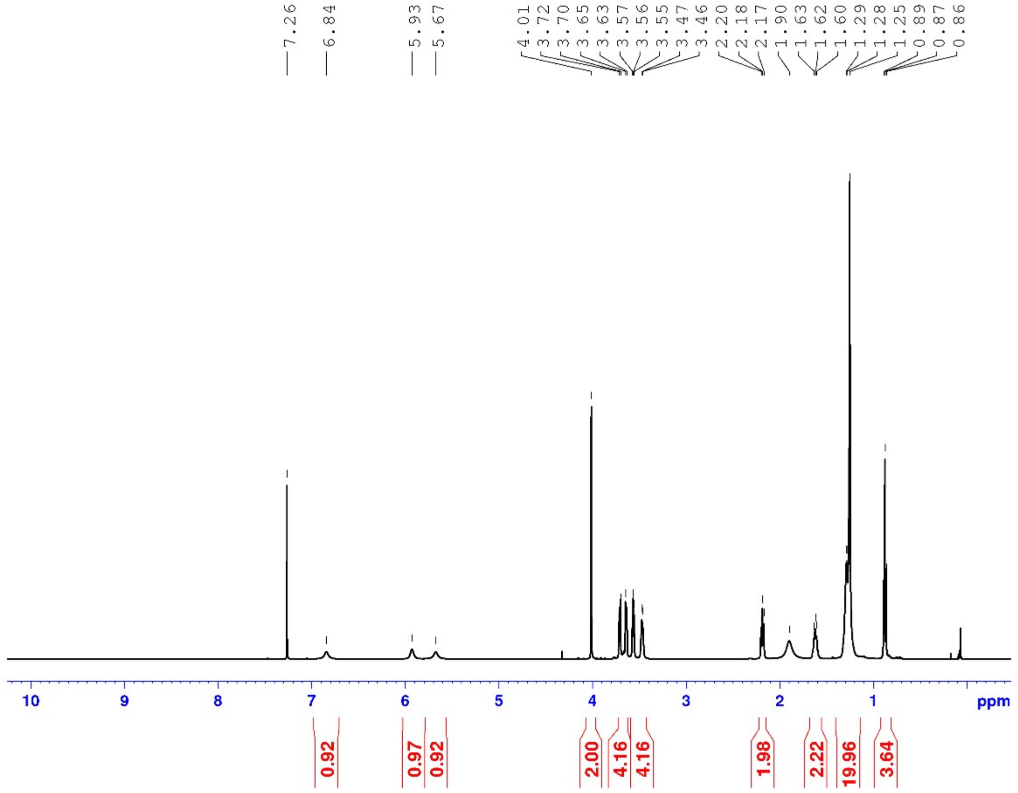

7.26
6.84
5.93
5.67
4.01
3.72
3.70
3.65
3.63
3.57
3.56
3.55
3.47
3.46
2.20
2.18
2.17
1.90
1.63
1.62
1.60
1.29
1.28
1.25
0.89
0.87
0.86
10 9 8 7 6 5 4 3 2 1 ppm
0.92
0.97
0.92
2.00
4.16
4.16
1.98
2.22
19.96
3.64

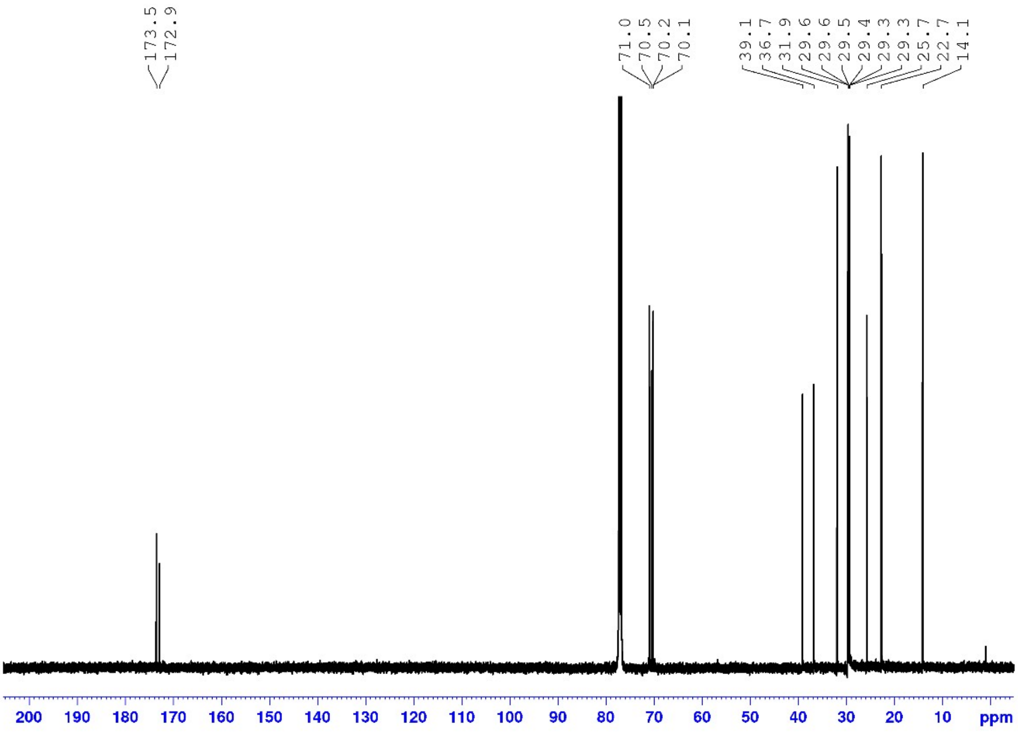

173.5
172.9
71.0
70.5
70.2
70.1
39.1
36.7
31.9
29.6
29.6
29.5
29.4
29.3
29.3
25.7
22.7
14.1
200 190 180 170 160 150 140 130 120 110 100 90 80 70 60 50 40 30 20 10 ppm

6

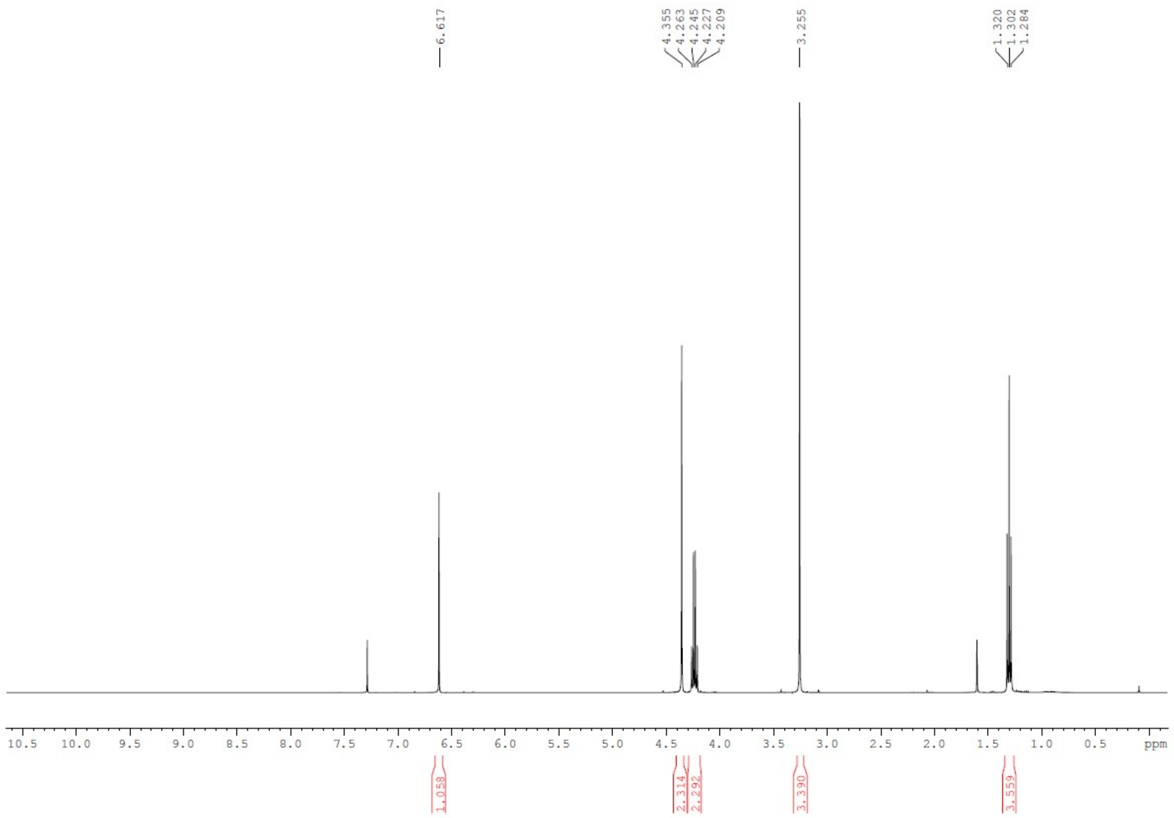
6.617
4.355
4.263
4.245
4.227
4.209
3.255
1.320
1.302
1.284
10.5 10.0 9.5 9.0 8.5 8.0 7.5 7.0 6.5 6.0 5.5 5.0 4.5 4.0 3.5 3.0 2.5 2.0 1.5 1.0 0.5 ppm
1.058
2.314
2.292
3.390
3.559

7

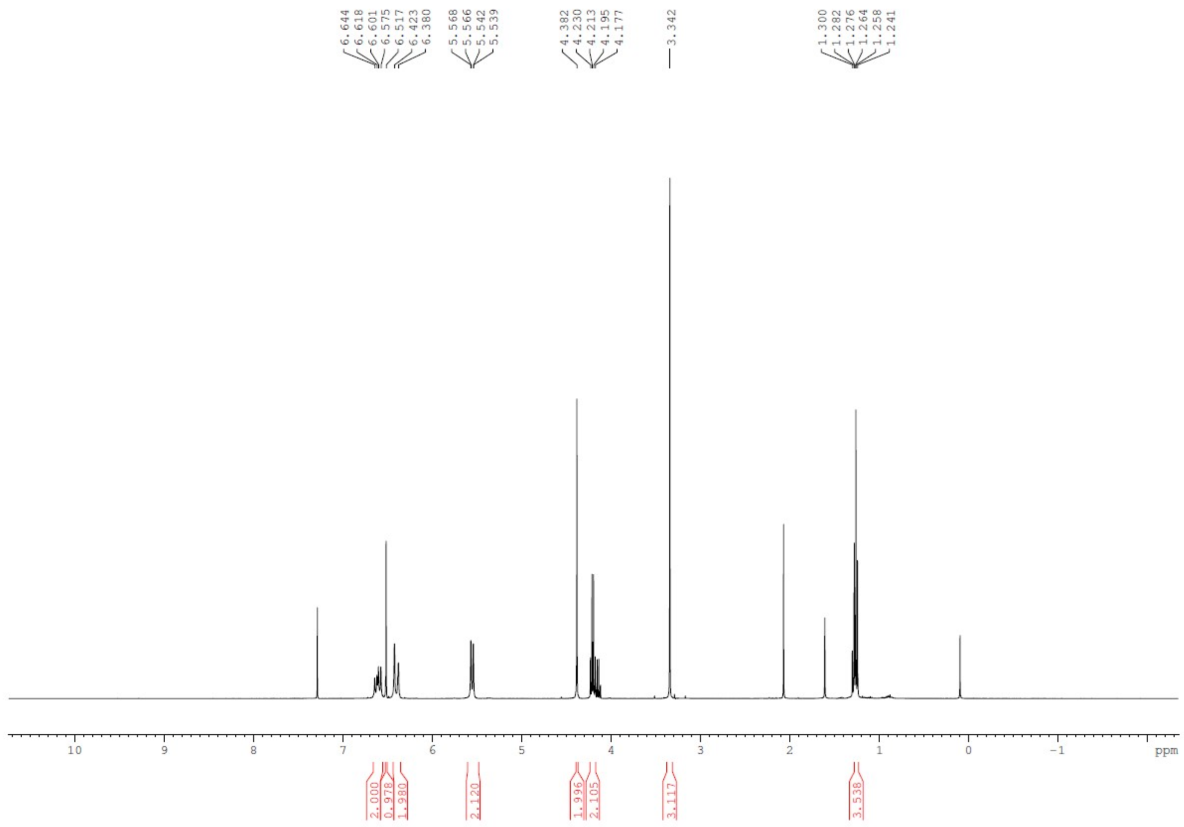
6.644
6.618
6.601
6.575
6.517
6.423
6.380
5.568
5.566
5.542
5.539
4.382
4.230
4.213
4.195
4.177
3.342
1.300
1.282
1.276
1.264
1.258
1.241
10 9 8 7 6 5 4 3 2 1 0 −1 ppm
2.000
0.978
1.980
2.120
1.996
2.105
3.117
3.538

8

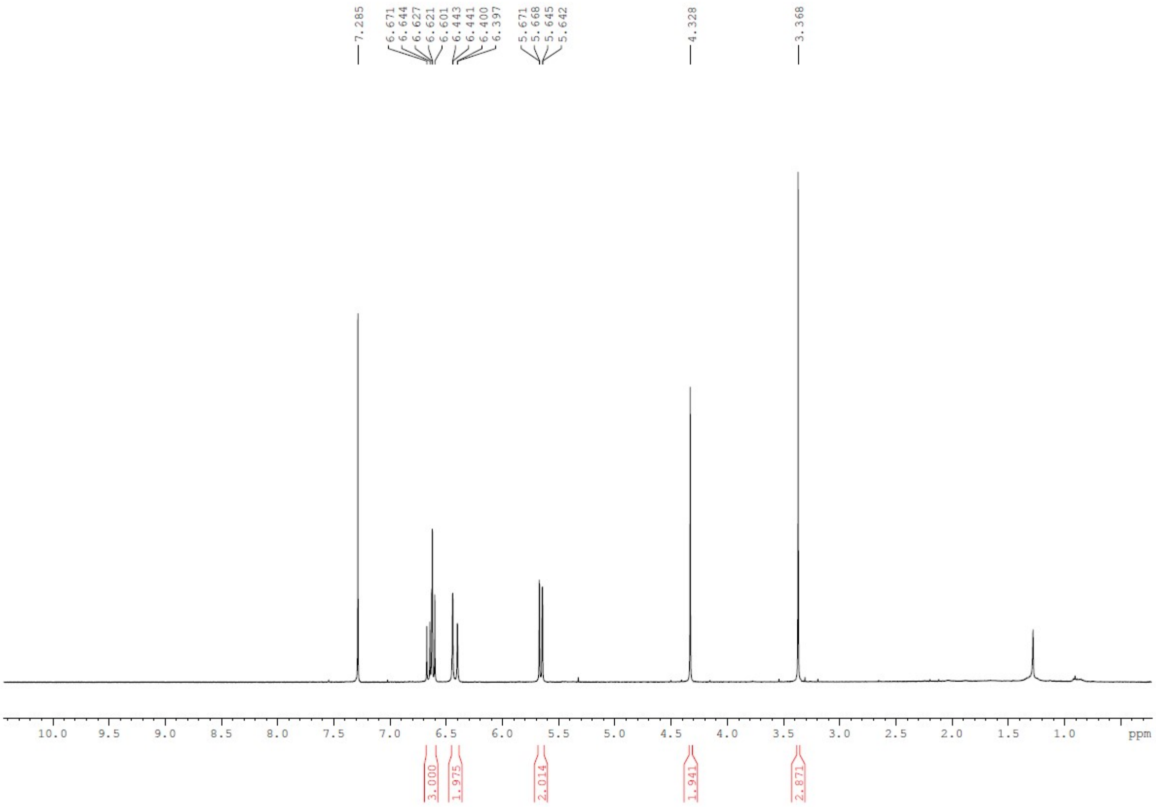

1.2.1.2 Peptides

General method 1: Manual solid-phase peptide synthesis (SPPS)

Manual peptide synthesis was performed on Merck LL MBHA Rink amide resin (0.33 mmol/g). Couplings were carried out by adding HATU (4 equiv) to a solution of the Fmoc-protected amino acid (4 equiv) in DMF (~0.4 M). After 10 seconds, DIPEA (8 equiv) was added to the mixture. This pre-activated mixture was then added to the resin in DMF and shaken for 3 min. The coupling time was extended in the case of coupling of Arg and unnatural amino acids (30 min). The side chain protecting groups used were: ^{t}Bu for Tyr; Boc for Lys, Trp; Pbf for Arg; Trt for Cys, homocysteine and His. Fmoc-Gly(Dmb)-OH was used for the synthesis of $\mathbf{P_{PL}}$. Fmoc-Lys(IvDde)-OH was used for the synthesis of Lys functionalised peptides **P8C9[PEG]** and **P8C9[FA]**.

Fmoc deprotection was carried out with 20% piperidine in DMF (3 x 3 min).

N-terminal acetyl capping was achieved with Ac_2O (2 equiv) using DIPEA (4 equiv) in CH_2Cl_2 for 1 hour at rt whilst shaking.

Completion of amide couplings and Fmoc deprotection was determined by a chloranil test, in which acetaldehyde (200 µL) and a saturated solution of chloranil in toluene (50 µL) were added to a small amount of resin swelled in CH_2Cl_2. After 10 seconds shaking at rt, no change in colour indicated complete coupling, whilst green colouration of the resin indicated presence of a free amine. Any incomplete couplings were submitted to a second round of coupling.

Side chain deprotection and cleavage from the resin was achieved with TFA containing 2.5% TIPS and 2.5% H_2O for 3 hours at rt or 1 hour at 42 °C. In case of cysteine-containing peptides, cleavage was achieved with TFA containing 5% EDT, 5% H_2O and 2.5% TIPS. After cleavage, the mixture was filtered through a sintered funnel, the beads washed with MeOH and the filtrate was concentrated under a stream of N_2. The crude residue was triturated with cold Et_2O before purification by preparative HPLC.

General method 2: Automated Fmoc solid-phase peptide synthesis (SPPS)

Automated peptide synthesis was carried out on a CEM Liberty Automated Microwave Peptide Synthesiser using Merck LL MBHA Rink Amide resin (0.46 mmol/g). All peptide couplings were performed with Fmoc-protected amino acids (5 equiv), Oxyma pure (10 equiv) and DIC (5 equiv) in DMF. Arg was coupled using double couplings for 15 min each without microwave irradiation. All other amino acids were coupled with 25 W power at 75 °C for 15 min.

Fmoc deprotection was achieved with a solution of 20% piperidine in DMF, using 45 W power at 75 °C for 3 min. *N*-terminal capping, cleavage and HPLC purification of peptides were carried out as previously described for manual SPPS (general method 1).

General method 3: Cyslisation *via* formation of a Trp-hCys cross bridge[7]

To a solution of a homocysteine and tryptophan-containing linear peptide (1 equiv) in H_2O-MeCN (0.5 mM) was added dropwise a solution of I_2 (3 equiv) dissolved in the minimum amount of MeCN and the resulting solution stirred at room temperature for 4 – 24 h. The reaction was then concentrated under a stream of N_2 and the crude reaction mixture dissolved in DMSO before being purified by preparative HPLC to yield the pure peptide.

P_{+}: Cyclisation *via* a copper-catalysed azido-alkyne click reaction[8]

A solution of diazido peptide **P_{+L}** (10.0 mg, 6.03 µmol, 1 equiv) and dialkynyl linker 3,5-diethynylbenzoic acid (1.0 mg, 6.03 µmol, 1 equiv) in 1:1 ^{t}BuOH/H_2O (12.5 mL, 0.8 mL/mg peptide) was degassed with N_2 for 15 min, followed by the addition of $CuSO_4 \cdot 5\,H_2O$ (1.5 mg, 6.03 µmol, 1 equiv), THPTA (3.2 mg, 6.03 µmol, 1 equiv) and sodium ascorbate (3.6 mg, 18.09 µmol, 3 equiv). The reaction

was stirred under N_2 and monitored by LCMS. When no starting material was detected, the reaction mixture was diluted with H_2O and lyophilized prior to purification by preparative HPLC (5-60 % B, 20 min). The absence of azido peak in IR (~ 2100 cm^{-1}) was checked for the purified, dried peptide (0.3 mg, 0.14 µmol, 2%).

P5C4: Cyclisation using DCA[9]

To a solution of **P5** (4.68 mg, 4 µmol, 1 equiv) in sodium phosphate buffer (1 mL, pH = 8.0, 50mM) was added TCEP·HCl (1.72 mg, 6 µmol, 1.5 equiv) and the solution stirred for 1 h. Subsequently, a solution of dichloroacetone (1.5 mg mg, 12 µmol, 3 equiv) in DMF (2 mL) was added and the solution shaken for 4 h. The solvent was removed under a stream of N_2 and the crude reaction mixture dissolved in DMSO before being purified by preparative HPLC (5-55 % B, 20 min) to yield the pure peptide (0.41 mg, 0.31 µmol, 8%).

P5C6: Cyclisation *via* the formation of a methylene bridge[10]

To a solution of **P5** (10.8 mg, 8.56 µmol, 1 equiv) in H_2O (0.72 mL) was added a solution of TCEP·HCl (3.68 mg, 12.85 µmol, 1.5 equiv) and K_2CO_3 (3.57 mg, 25.70 µmol, 3 equiv) in H_2O (1.33 mL). The reaction was monitored by LCMS. Upon completion of this step, a solution of NEt_3 (6.00 µL, 42.8 µmol, 5 equiv) in THF (0.12 mL) and diiodomethane (9.21 mg, 34.24 µmol, 4 equiv) in THF (0.12 mL) were added sequentially. The reaction mixture was stirred at rt for 9 h. Solvent was removed under N_2 and the crude reaction mixture dissolved in DMSO prior to purification by preparative HPLC (5-60 % B, 20 min) to obtain the pure peptide (2.04 mg, 1.61 µmol, 19%).

P5C7: Cyclisation using a divynylpyrimidine[11,12]

To a solution of **P5** (16.4 mg, 13 µmol, 1 equiv) in sodium phosphate buffer (2 mL, pH = 8.0, 50mM) and DMF (6.2 mL) was added **8** (*N*-(4,6-divinylpyrimidin-2-yl)-*N*-methylglycine, 3.1 mg, 14 µmol, 1.1 equiv) and the reaction stirred at room temperature overnight. Solvent was removed under N_2 and the crude reaction mixture dissolved in DMSO prior to purification by preparative HPLC (10-70 % B, 20 min) to obtain the pure peptide (3.30 mg, 2.23 µmol, 17%).

P6C2: Cyclisation *via* a copper-catalysed azido-alkyne click reaction[8,13]

A solution of azido-alkynyl peptide **P6** (31.0 mg, 41 µmol, 1 equiv) in H_2O (31 mL, 1 mL/mg peptide) was degassed with N_2 for 15 min, followed by the addition of $CuSO_4 \cdot 5\,H_2O$ (10.2 mg, 41 µmol, 1 equiv), sodium ascorbate (8.1 mg, 41 µmol, 1 equiv) and DIPEA (57.1 µL, 328 µmol, 8 equiv). The reaction was stirred under N_2 and monitored by LCMS. When no starting material was detected, the reaction mixture was diluted with H_2O and lyophilized prior to purification by preparative HPLC (20-55 % B, 20

min). The absence of azido peak in IR (~ 2100 cm^{-1}) was checked for the purified, dried peptide (7.6 mg, 6 µmol, 19%).

P6C3: Cyclisation *via* a ruthenium-catalysed azido-alkyne click reaction [14]

A solution of azido-alkynyl peptide **P6** (11.8 mg, 9 µmol, 1 equiv) in 1:1 $^{t}BuOH/H_2O$ (14.8 mL, 0.8 mL/mg peptide) was degassed with N_2 for 15 min, followed by the addition of chloro(pentamethylcyclopentadienyl)ruthenium (II) tetramer (3.06 mg, 3 µmol, 30 mol%). The reaction was stirred under N_2 overnight. Solvent was removed under a stream of N_2 and the crude reaction mixture dissolved in DMSO before being purified by preparative HPLC (20-40 % B, 20 min) to yield the pure peptide (0.30 mg, 0.24 µmol, 3%).

P7C8: Cyslisation *via* formation of a Trp-Cys cross bridge[7]

To a solution of **P7** (43.0 mg, 32 µmol, 1 equiv) in H_2O-MeCN (1:1, 64 mL) was added dropwise a solution of I_2 (16.2 mg, 64 µmol, 2 equiv) in MeCN (5 mL) and the resulting solution stirred at room temperature for 6 h. The reaction was then washed with CH_2Cl_2 (2 x 30 mL) and the solvent removed under a stream of N_2. The crude reaction mixture was dissolved in DMSO before being purified by preparative HPLC (20-70 % B, 20 min) to yield the pure peptide (10.6 mg, 7.9 µmol, 25%).

1.2.1.2.1 LCMS and purity of peptides

Table S2 Sequence, mass, purity and retention time of CK2 peptides from LCMS and HPLC analysis.

Peptide	Sequence	Mass	*m/z* found	*m/z* calc.	Species	Purity (%)	Rt* (min)
**P1**	RLYGFK	823.5	824.7	824.5	M+H	95	6.84
**P2**	LYGFKW	853.5	854.5	854.5	M+H	97	8.75
**P3**	RLYGFKW	1009.6	1011.0	1010.6	M+H	93	7.92
**P4**	RLYGFKWH	1146.6	1164.7	1164.6	M+NH$_4$	96	7.08
**P$_{+L}$**	GXRLYGFKWHXGG	1541.2	1544.2	1542.8	M+H	97	8.20
**P$_{+}$**	GX$_{C1}$RLYGFKWHX$_{C1}$GG	1711.8	1711.6	1710.8	M-H	80	8.08
**P5**	CRLYGFKC	1029.5	1030.8	1030.5	M+H	96	7.42
**P6**	JRLYGFKX	1030.6	1032.6	1031.6	M+H	96	7.41
**P7**	CRLYGFKW	1112.5	1114.0	1113.5	M+H	95	7.81
**P8**	(hC)RLYGFKW	1126.6	1128.1	1127.6	M+H	95	8.15
**P6C2**	J$_{C2}$RLYGFKX$_{C2}$	1030.6	1032.0	1031.6	M+H	98	6.82
**P6C3**	J$_{C3}$RLYGFKX$_{C3}$	1030.6	1091.8	1091.6	M+^{i}PrOH+H	96	8.02
**P5C4**	C$_{C4}$RLYGFKC$_{C4}$	1083.5	1147.6	1147.5	M+MeCN+Na	90	7.21
**P5C5**	C$_{C5}$RLYGFKC$_{C5}$	1131.5	1132.9	1132.5	M+H	95	8.29
**P5C6**	C$_{C6}$RLYGFKC$_{C6}$	1041.5	1043.6	1042.5	M+H	92	7.05
**P5C7**	C$_{C7}$RLYGFKC$_{C7}$	1248.6	1250.3	1249.6	M+H	74	7.40
**P7C8**	C$_{C8}$RLYGFKW$_{C8}$	1110.5	1112.6	1111.5	M+H	94	7.98
**P8C9**	(hC)$_{C9}$RLYGFKW$_{C9}$	1124.6	1126.5	1125.6	M+H	97	7.76
**P$_{-}$**	HWKFGYLR	1146.6	1148.8	1147.6	M+H	80	7.36
**FA-P8**	(LA)(PEG)(hC)RLYGFKW	1411.8	1411.1	1410.8	M-H	90	10.93
**FA-P8C9**	(LA)(PEG)(hC)$_{C9}$RLYGFKW$_{C9}$	1409.8	1411.7	1410.8	M+H	99	10.56
**PEG-P8**	(PEG)(hC)RLYGFKW	1271.7	1274.1	1272.7	M+H	86	7.93
**PEG-P8C9**	(PEG)(hC)$_{C3}$RLYGFKW$_{C3}$	1269.6	1271.1	1270.6	M+H	98	7.52
**P8[FA]**	(hC)RLYGFK(PEG-(LA))W	1453.8	1455.6	1454.8	M+H	98	11.73
**P8C9[FA]**	(hC)$_{C9}$RLYGFK(PEG-(LA))W$_{C9}$	1451.8	1453.6	1452.8	M+H	92	11.37
**P8[PEG]**	(hC)RLYGFK(PEG-Ac)W	1313.7	1314.9	1314.7	M+H	97	8.47
**P8C9[PEG]**	(hC)$_{C3}$RLYGFK(PEG-Ac)W$_{C3}$	1311.6	1313.4	1312.7	M+H	>99	7.61
**FA-PEG**[1]	(LA)(PEG)	344.3	345.2	345.3	M+H	N/A	N/A
**TAT-P8**[2]	GRKKRRQRRRPPQ(Ahx)(Ahx)<br>(hC)RLYGFKW	3010.8	753.7	753.7	M+4H	62	5.95
**TAT-P8C9**[2]	GRKKRRQRRRPPQ(Ahx)(Ahx)<br>(hC)$_{C9}$RLYGFKW$_{C9}$	3008.8	753.7	753.2	M+4H	>99	5.55
**TAT-**	GRKKRRQRRRPPQ(Ahx)(Ahx)	1943.2	649.0	648.8	M+3H	98	3.75

**(Ahx)$_2$[2]**							
**R3-P8[2]**	RRR(Ahx)(Ahx)(hC)RLYGFKW	1779.0	890.9	890.5	M+2H	98	6.31
**R3-P8C9[2]**	RRR(Ahx)(Ahx)(hC)$_{C9}$RLYGFK W$_{C9}$	1777.0	890.0	889.5	M+2H	99	5.96
**R3-(Ahx)$_2$[2]**	RRR(Ahx)(Ahx)	711.5	710.5	710.5	M-H	>99	6.70

X = Aza-alanine. J = Propargylglycine. (hC) = Homocysteine. (LA) = Lauric acid. (PEG) = 2-(2-(2-aminoethoxy)ethoxy)acetic acid. (Ahx) = 6-aminohexanoic acid. All peptides feature an amide at the *C*-terminus and an acetyl capping at the *N*-terminus unless otherwise stated. [1]This compound is not visible by HPLC and thus no HPLC traces are reported. [2]Peptides feature a free amine at the *N*-terminus.

1.2.1.2.2 Yields of peptides

Table S3 Sequence and yield of peptides.

Peptide	Sequence	Yield (%)
**P1**	RLYGFK	9.8
**P2**	LYGFKW	25.8
**P3**	RLYGFKW	8.4
**P4**	RLYGFKWH	4.7
**P$_{+L}$**	GXRLYGFKWHXGG	2.5
**P$_{+}$**	GX$_{C1}$RLYGFKWHX$_{C1}$GG	2.3
**P5**	CRLYGFKC	0.8
**P6**	JRLYGFKX	5.0
**P7**	CRLYGFKW	23.9
**P8**	(hC)RLYGFKW	2.3
**P6C2**	J$_{C2}$RLYGFKX$_{C2}$	14.6
**P6C3**	J$_{C3}$RLYGFKX$_{C3}$	1.3
**P5C4**	C$_{C4}$RLYGFKC$_{C4}$	7.8
**P5C5**	C$_{C5}$RLYGFKC$_{C5}$	8.3
**P5C6**	C$_{C6}$RLYGFKC$_{C6}$	20.0
**P5C7**	C$_{C7}$RLYGFKC$_{C7}$	17.2
**P7C8**	C$_{C8}$RLYGFKW$_{C8}$	24.6
**P8C9**	(hC)$_{C9}$RLYGFKW$_{C9}$	28.4
**P$_{-}$**	HWKFGYLR	21.7
**FA-P8**	(LA)(PEG)(hC)RLYGFKW	18.1
**FA-P8C9**	(LA)(PEG)(hC)$_{C9}$RLYGFKW$_{C9}$	23.5
**PEG-P8**	(PEG)(hC)RLYGFKW	13.5
**PEG-P8C9**	(PEG)(hC)$_{C3}$RLYGFKW$_{C3}$	11.8
**P8[FA]**	(hC)RLYGFK(PEG-(LA))W	9.8
**P8C9[FA]**	(hC)$_{C9}$RLYGFK(PEG-(LA))W$_{C9}$	24.4
**P8[PEG]**	(hC)RLYGFK(PEG-Ac)W	10.4
**P8C9[PEG]**	(hC)$_{C3}$RLYGFK(PEG-Ac)W$_{C3}$	34.1

**FA-PEG**	(LA)(PEG)	89.4
**TAT-P8**[2]	GRKKRRQRRRPPQ(Ahx)(Ahx)(hC)RLYGFKW	3.9
**TAT-P8C9**[2]	GRKKRRQRRRPPQ(Ahx)(Ahx)(hC)$_{C9}$RLYGFKW$_{C9}$	37.8
**TAT-(Ahx)$_2$**[2]	GRKKRRQRRRPPQ(Ahx)(Ahx)	18.8
**R3-P8**[2]	RRR(Ahx)(Ahx)(hC)RLYGFKW	1.4
**R3-P8C9**[2]	RRR(Ahx)(Ahx)(hC)$_{C9}$RLYGFKW$_{C9}$	37.8
**R3-(Ahx)$_2$**[2]	RRR(Ahx)(Ahx)	24.4

X = Aza-alanine. J = Propargylglycine. (hC) = Homocysteine. (LA) = Lauric acid. (PEG) = 2-(2-(2-aminoethoxy)ethoxy)acetic acid. (Ahx) = 6-aminohexanoic acid. All peptides feature an amide at the *C*-terminus and an acetyl capping at the *N*-terminus unless otherwise stated. [2]Peptides feature a free amine at the *N*-terminus.

1.2.1.2.3 HPLC traces of peptides

P1

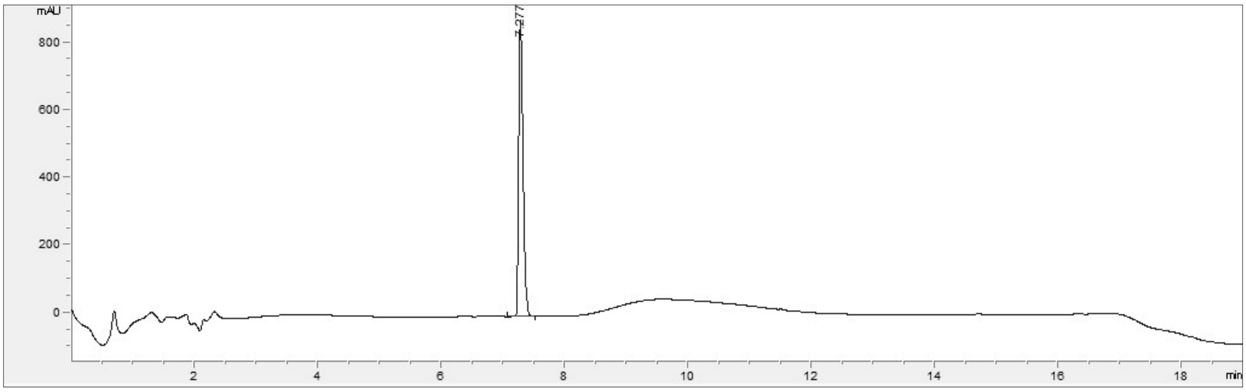

10-60% B, 15 min

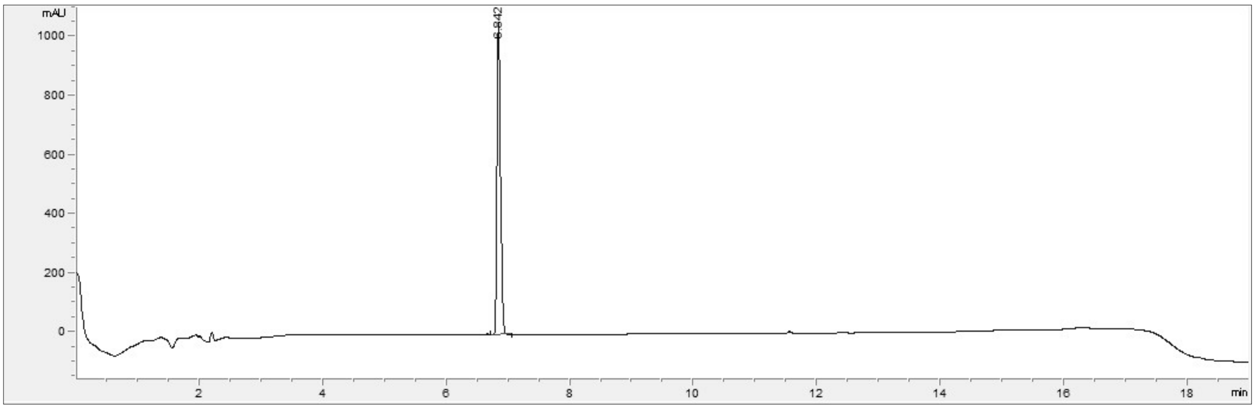

5-95% B, 15 min

P2

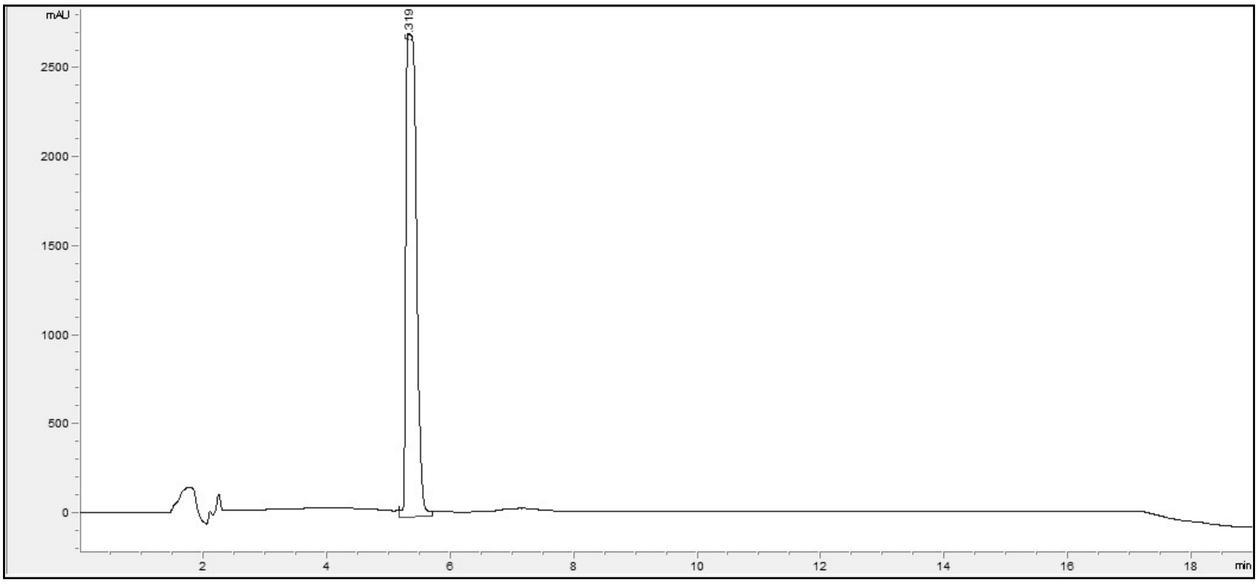

30-60% B, 15 min

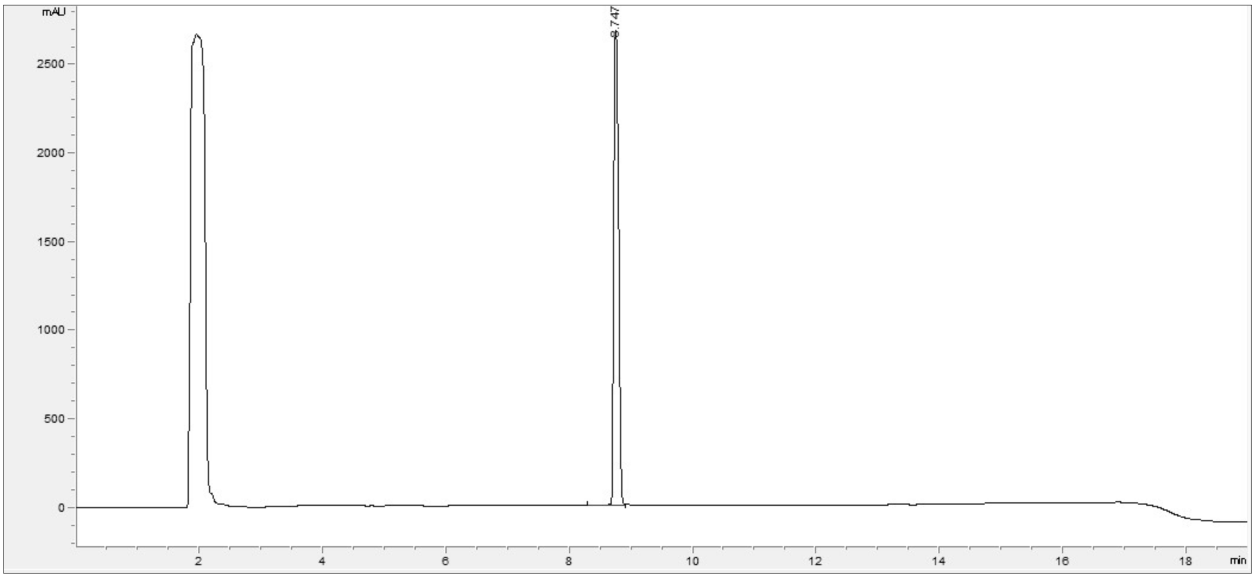

5-95% B, 15 min

P3

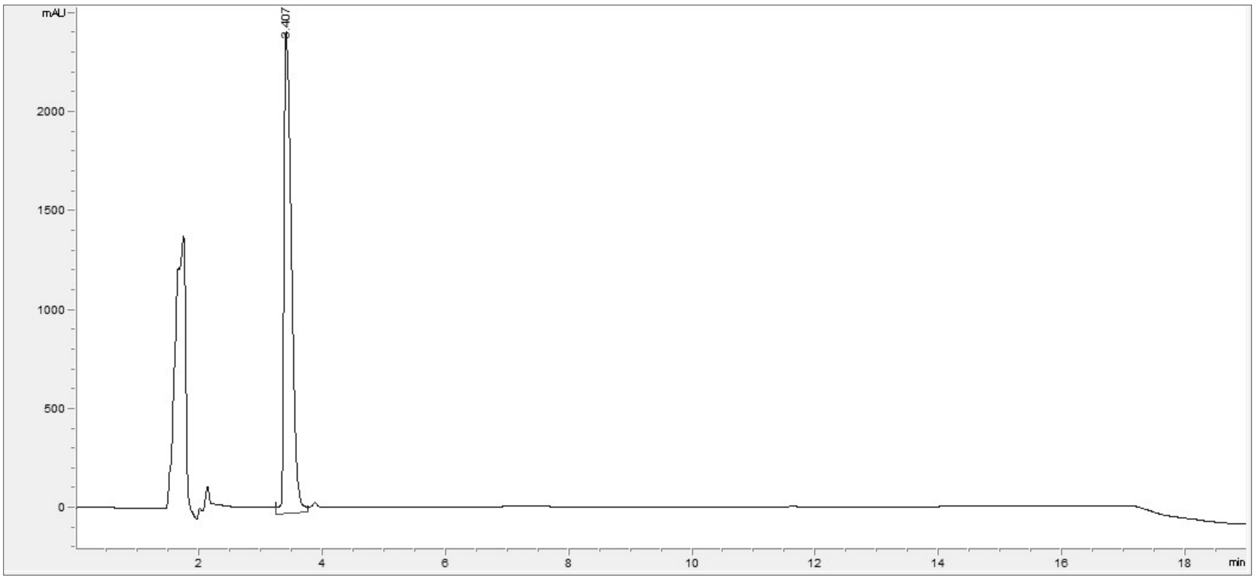

30-60% B, 15 min

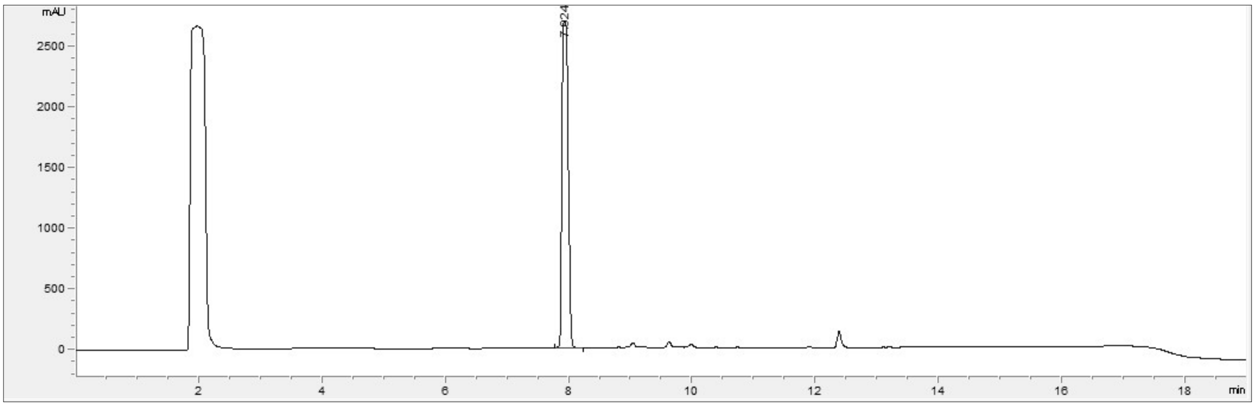

5-95% B, 15 min

P4

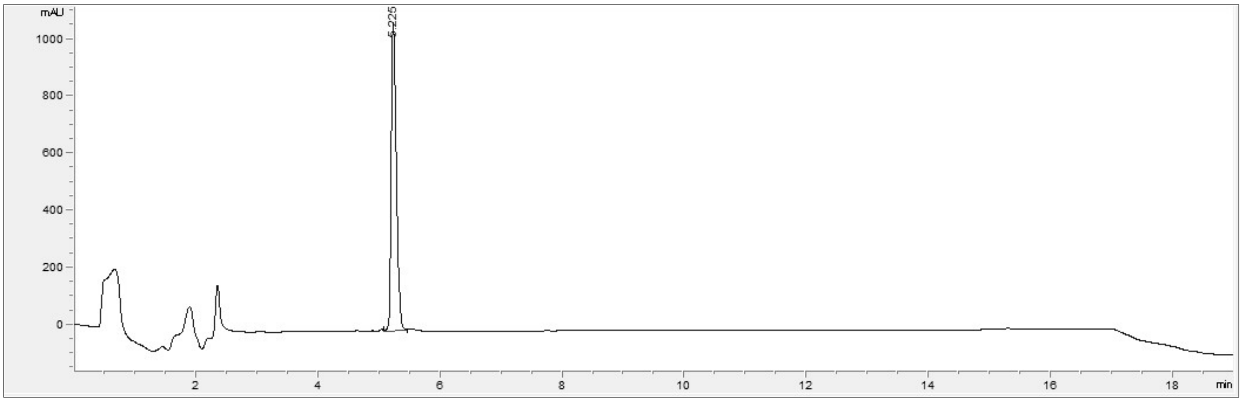

20-60% B, 15 min

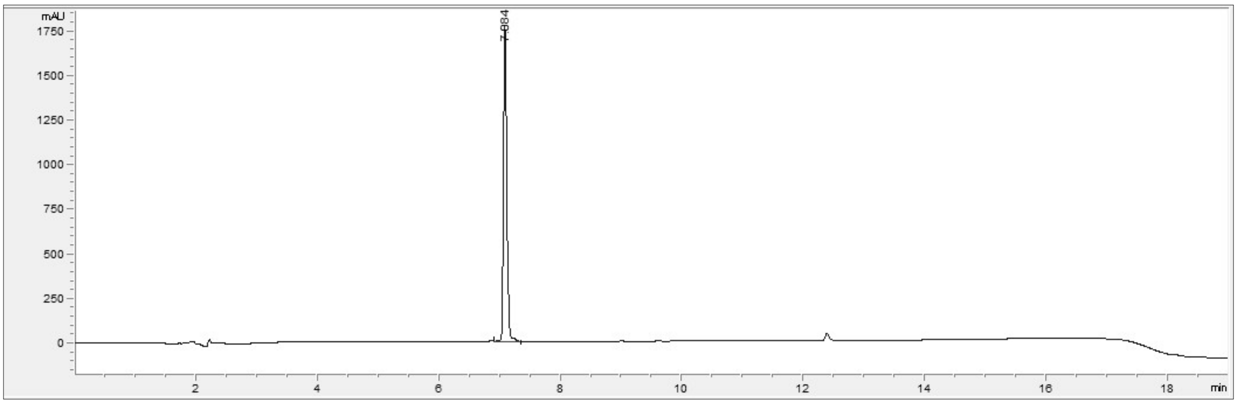

5-95% B, 15 min

P5

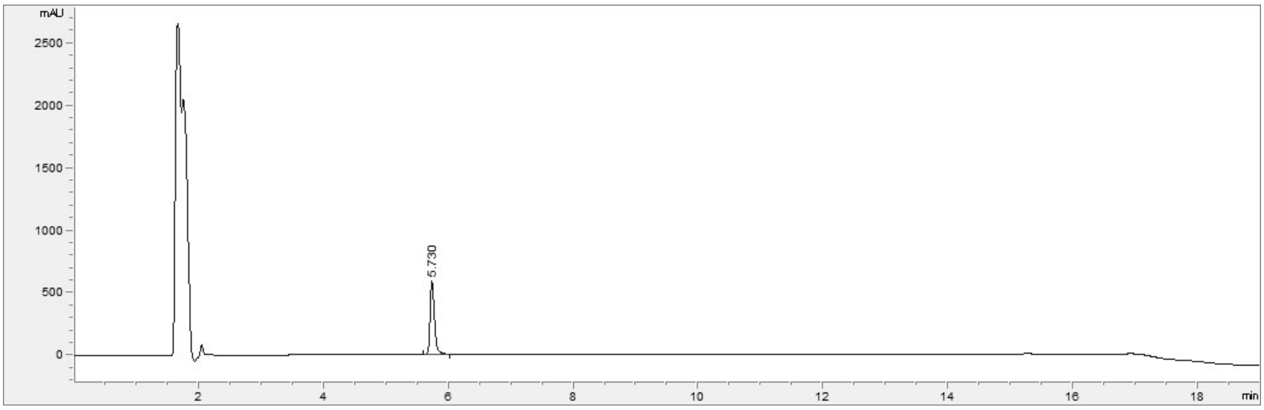

20-60% B, 15 min

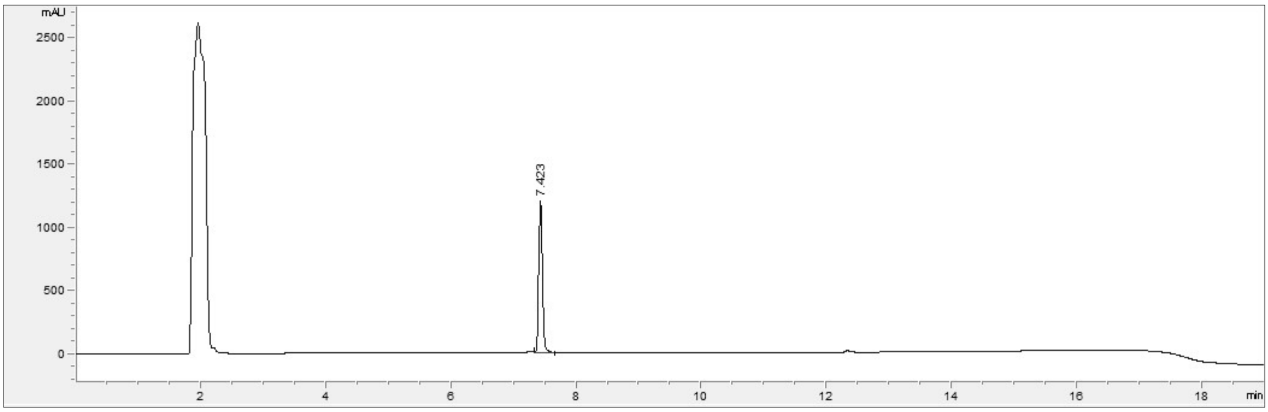

5-95% B, 15 min

P6

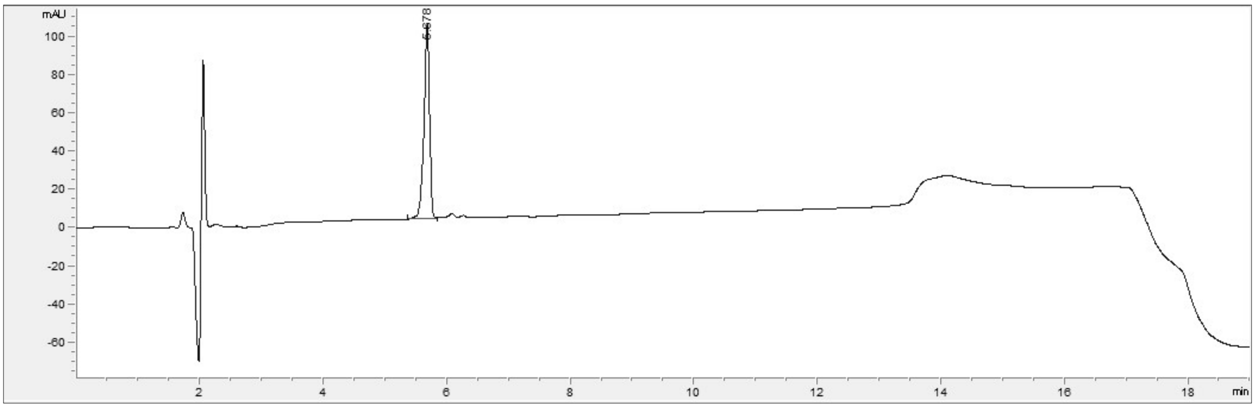

20-60% B, 15 min

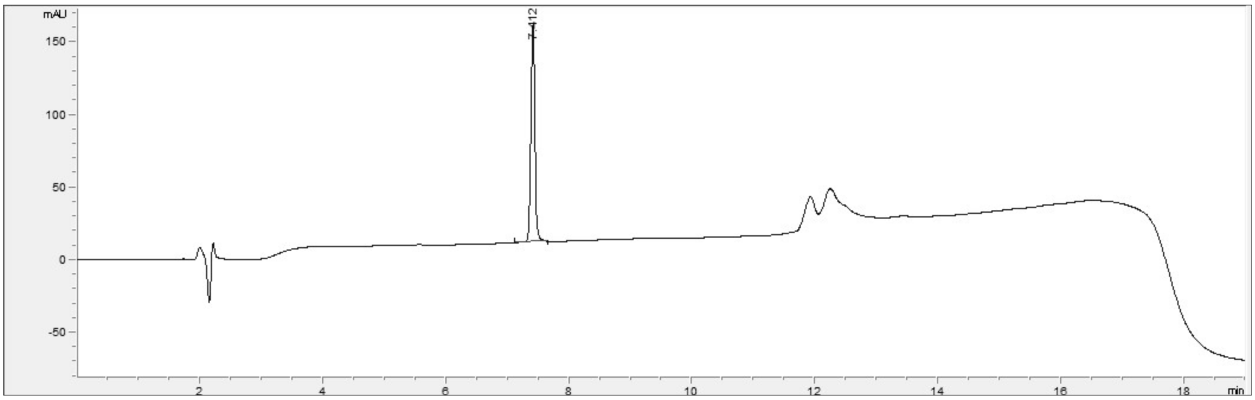

5-95% B, 15 min

P7

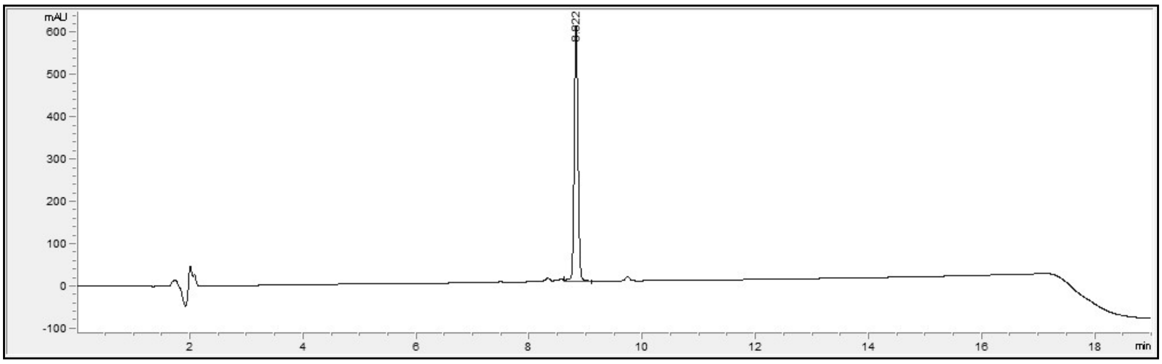

30-80% B, 15 min

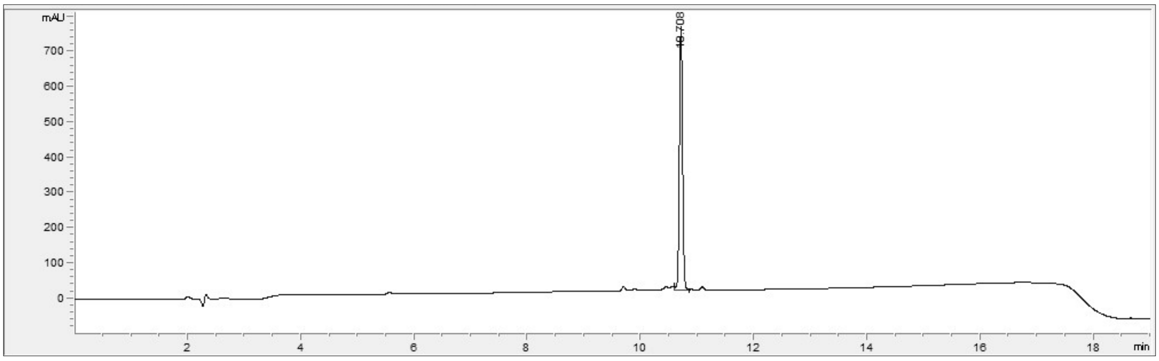

5-95% B, 15 min

P8

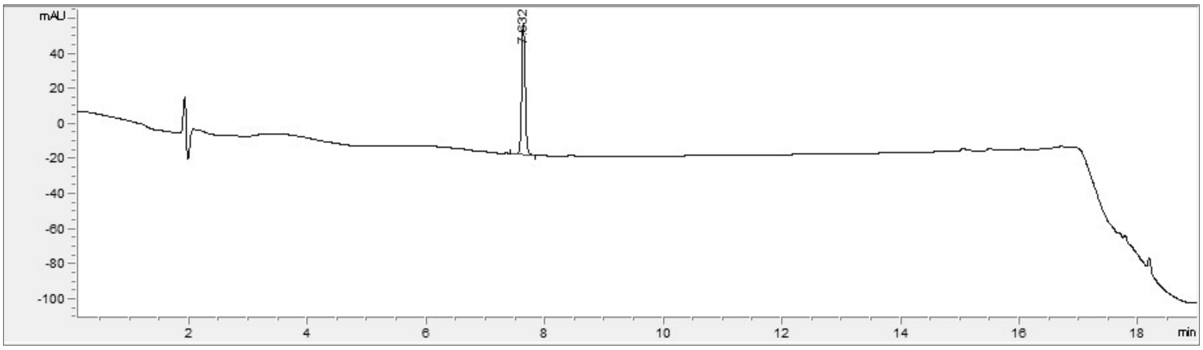

20-60% B, 15 min

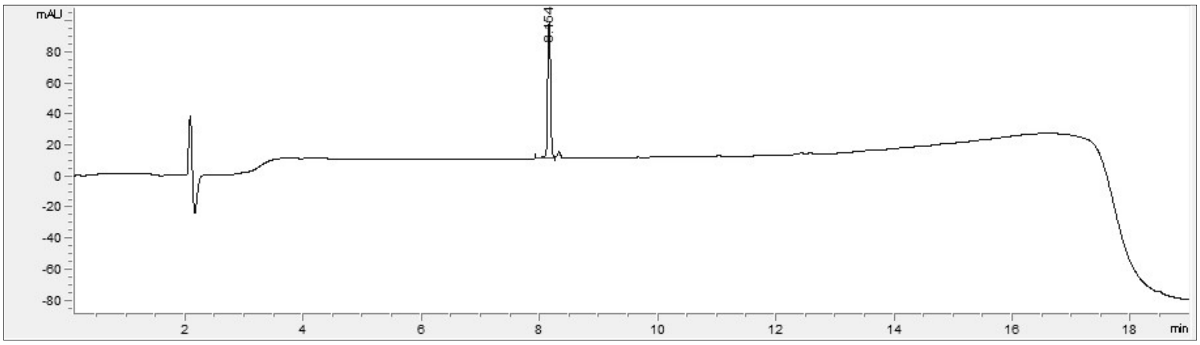

5-95% B, 15 min

P_-

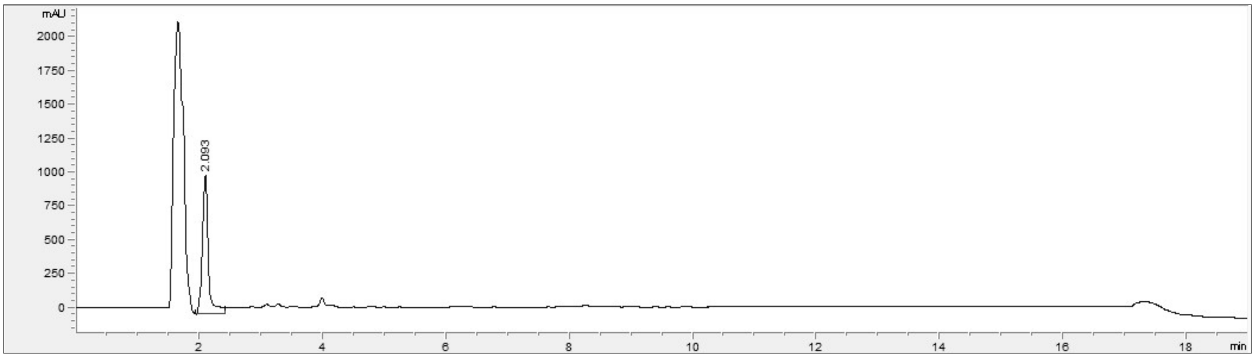

30-60% B, 15 min

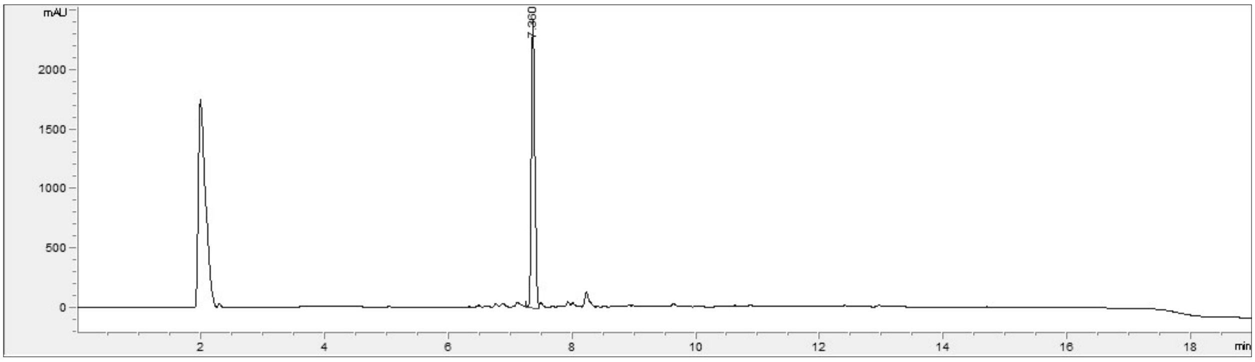

5-95% B, 15 min

P_{+L}

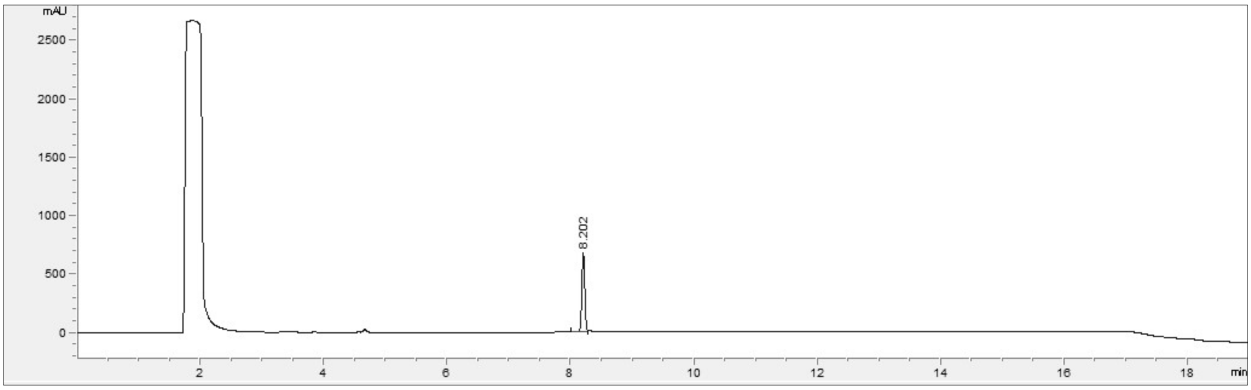

10-60% B, 15 min

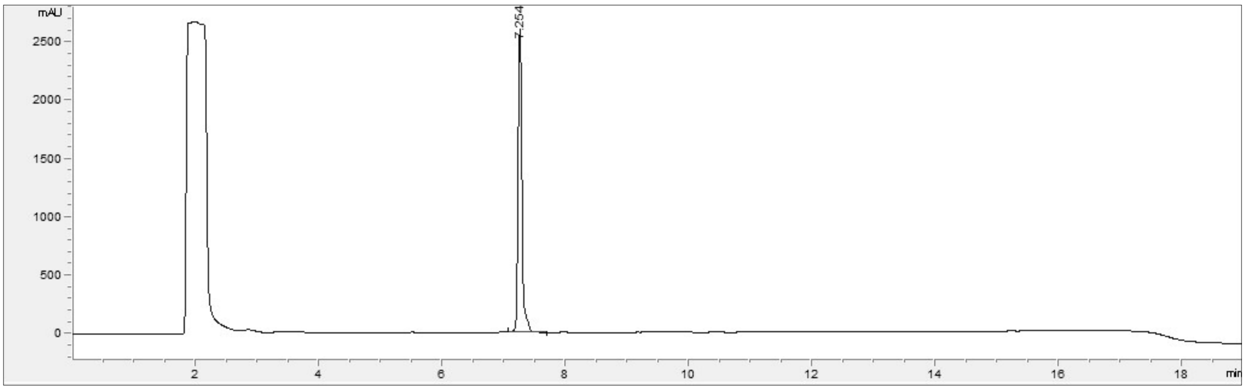

5-95% B, 15 min

P₊

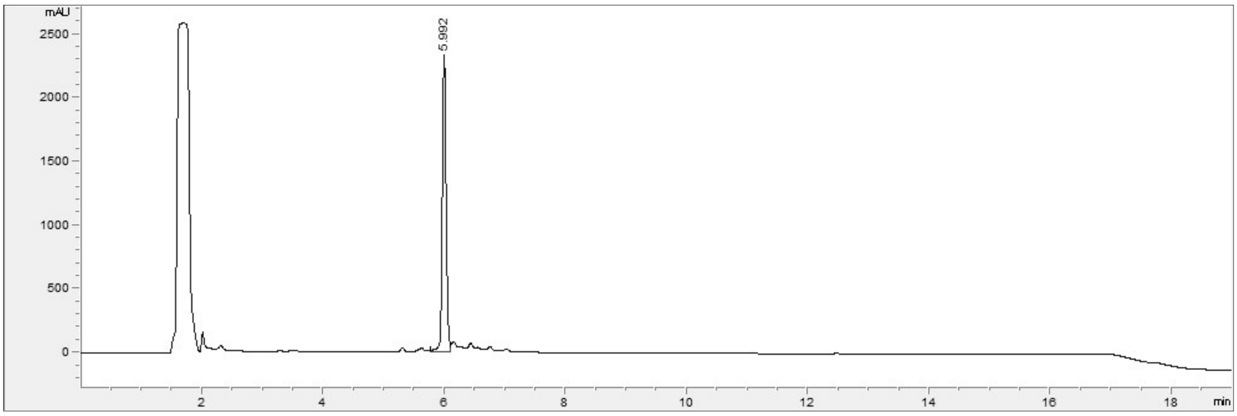

20-60% B, 15 min

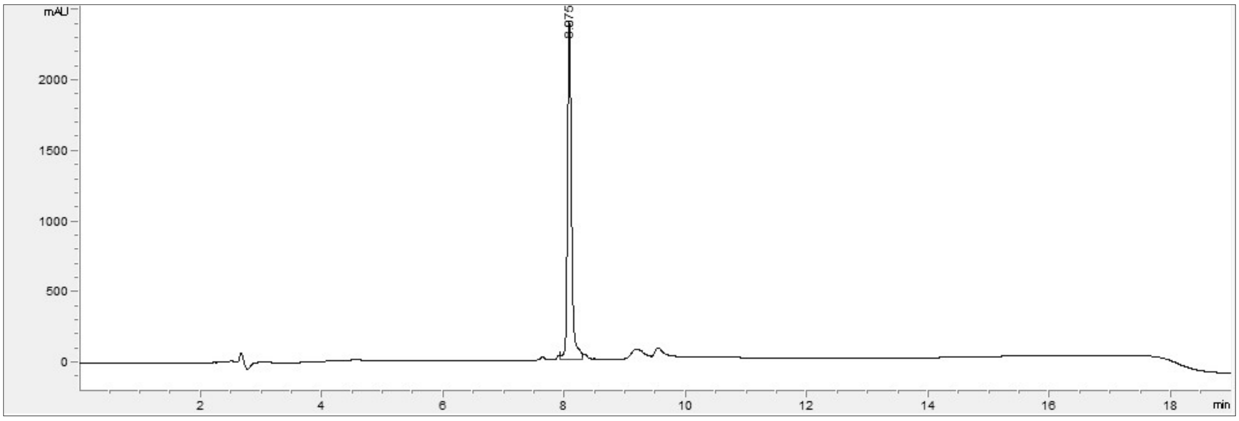

5-95% B, 15 min

P5C4

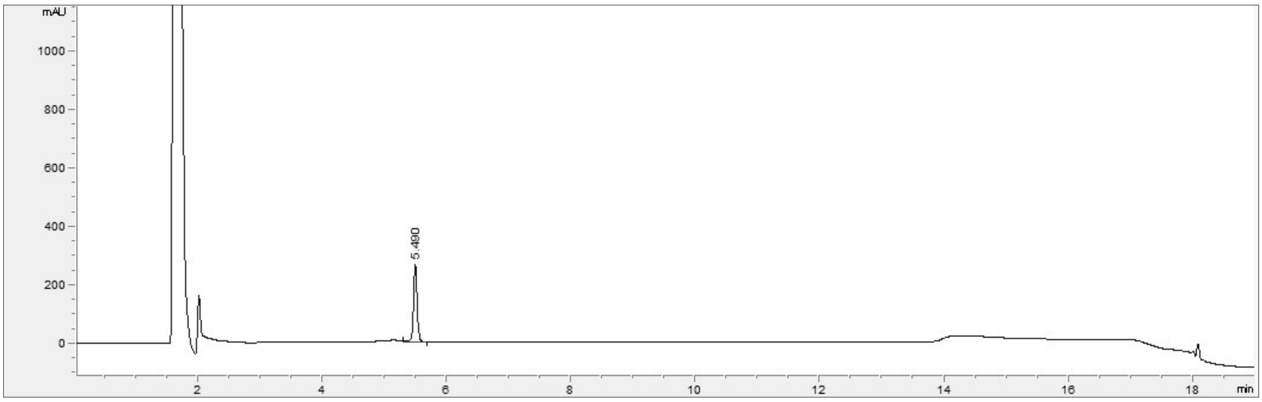

20-60% B, 15 min

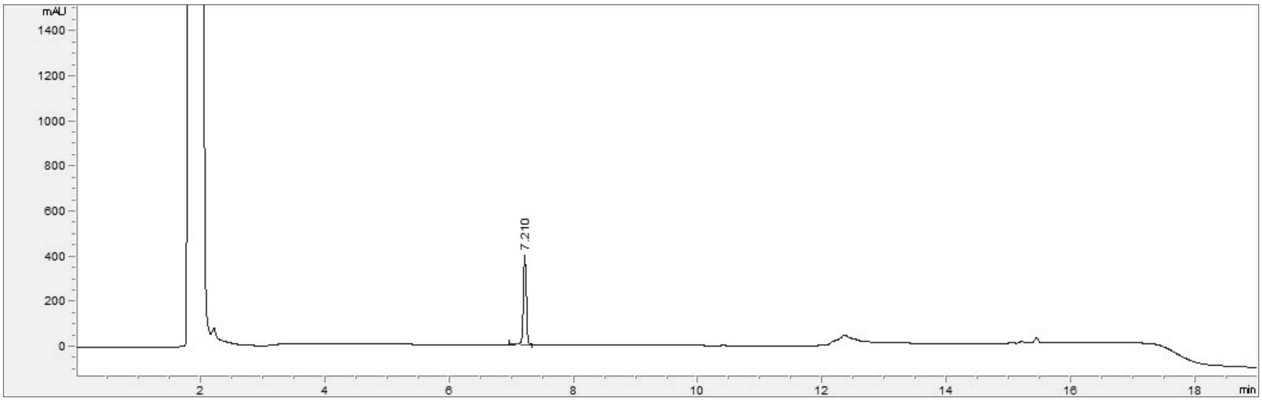

5-95% B, 15 min

P5C5

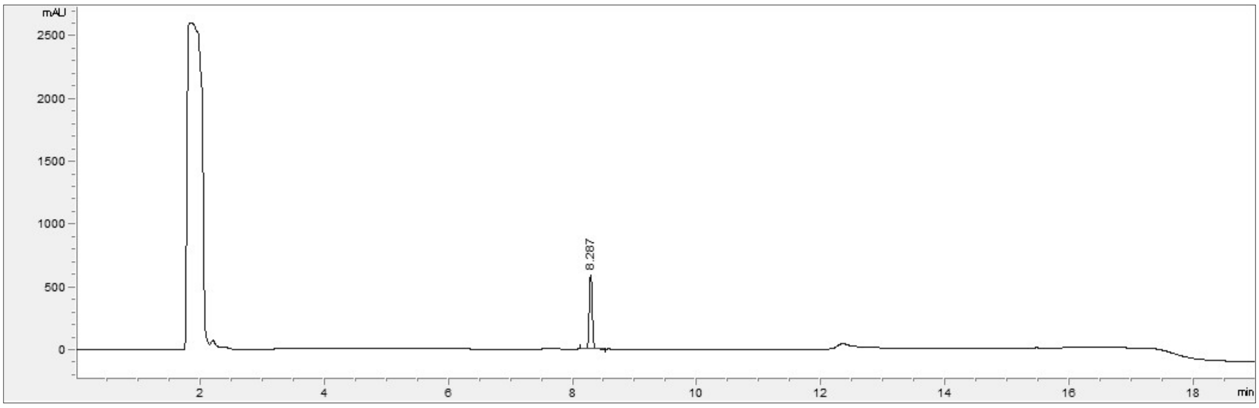

5-95% B, 15 min

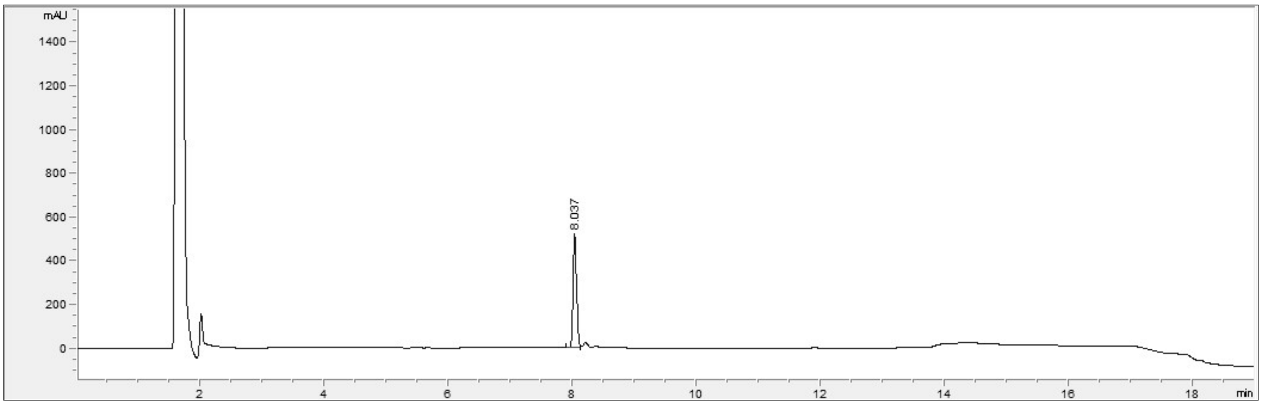

20-60% B, 15 min

P5C6

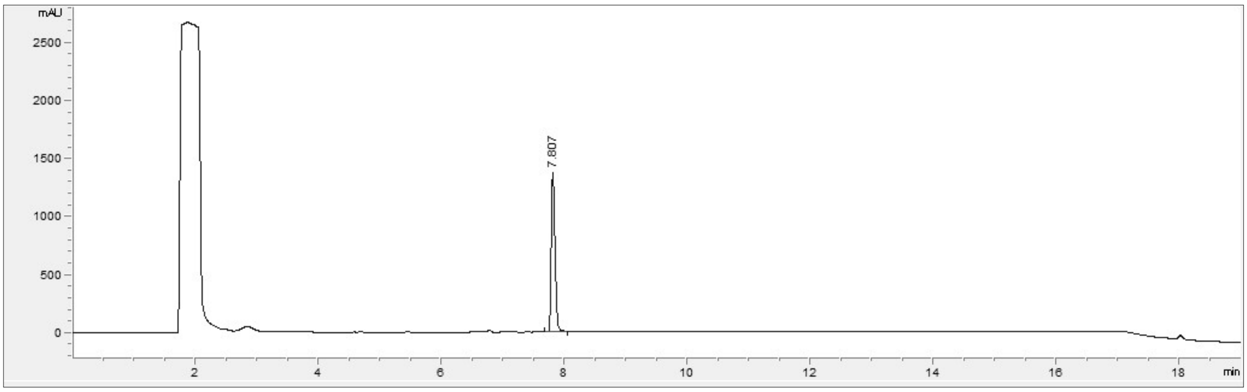

10-60% B, 15 min

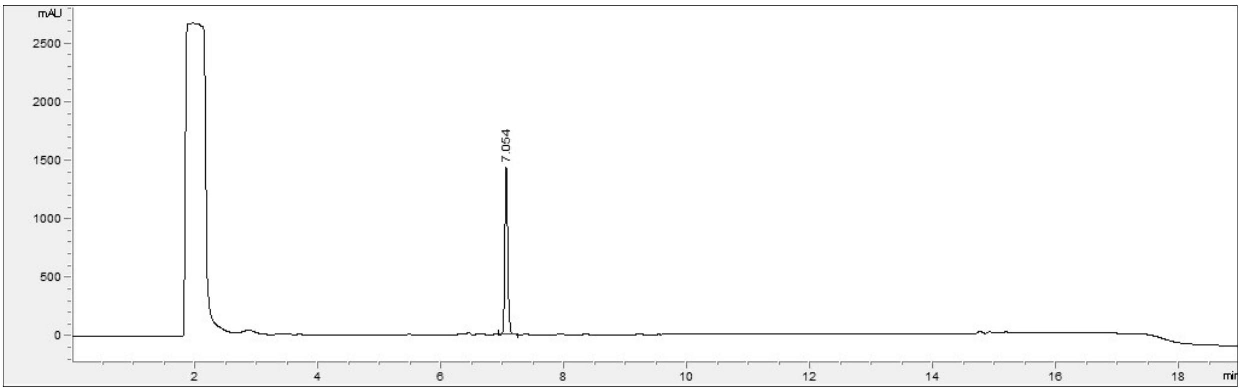

5-95% B, 15 min

P5C7

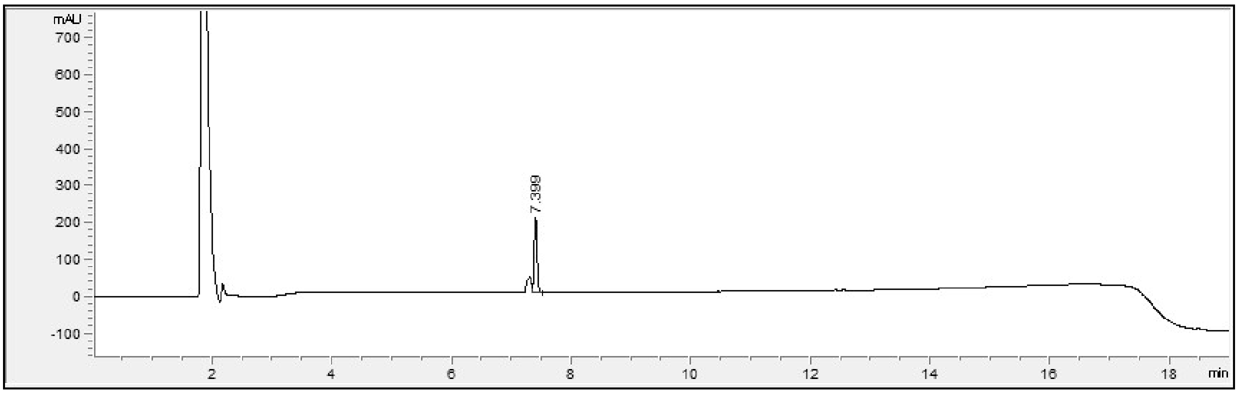

5-95% B, 15 min

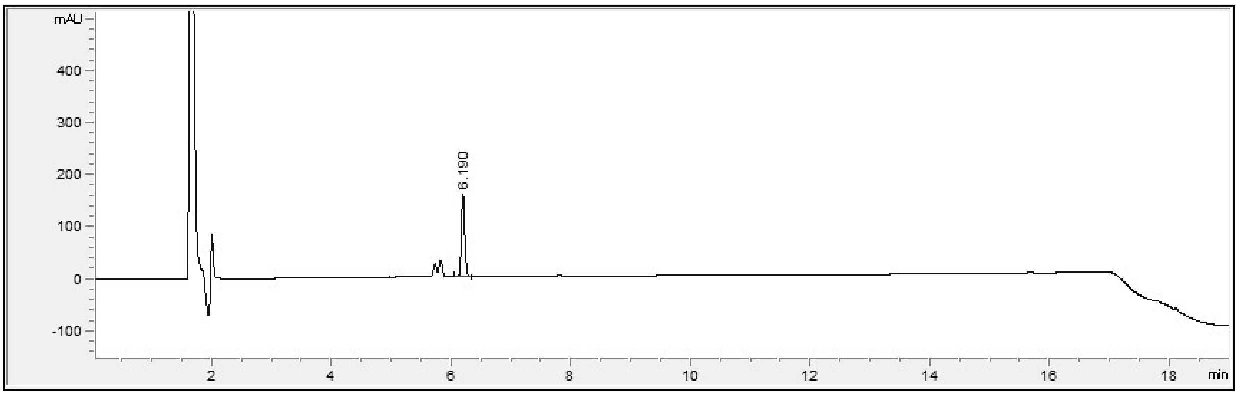

20-60% B, 15 min

P6C2

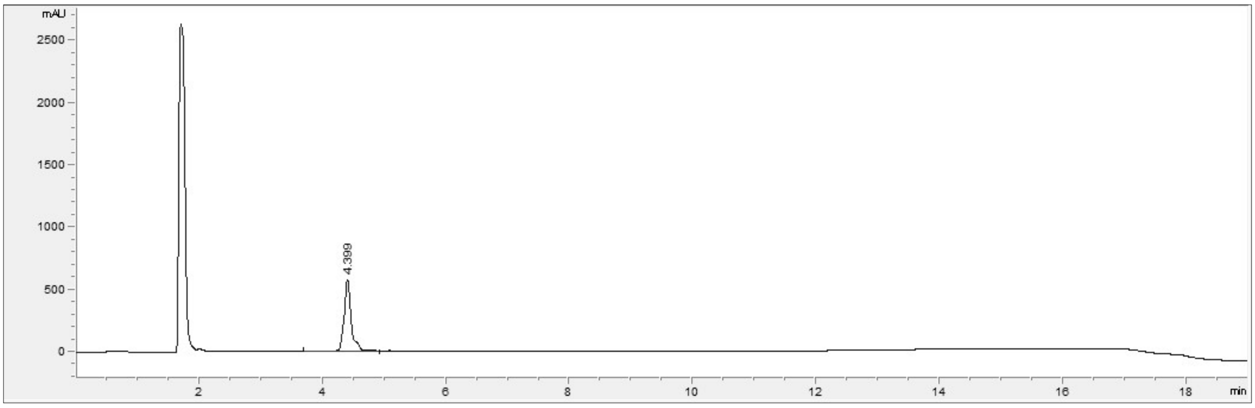

20-60% B, 15 min

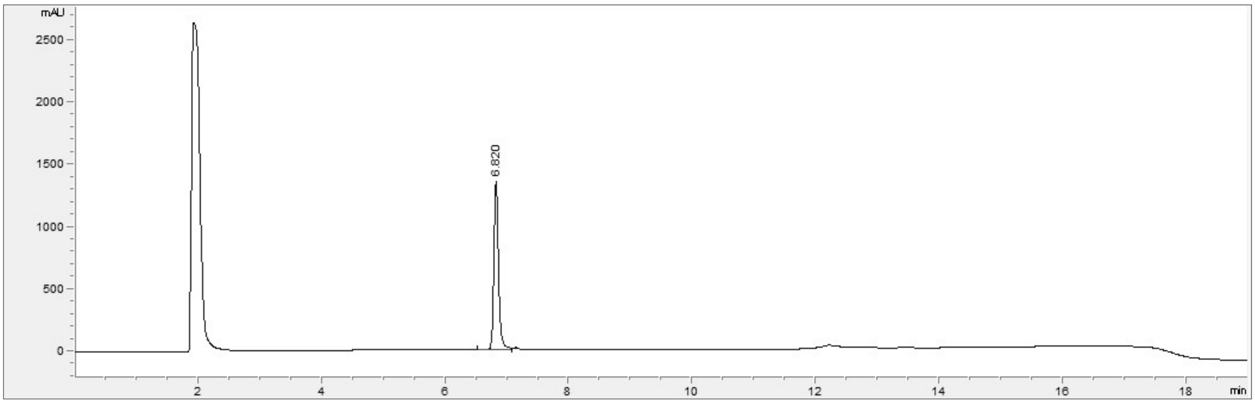

5-95% B, 15 min

P6C3

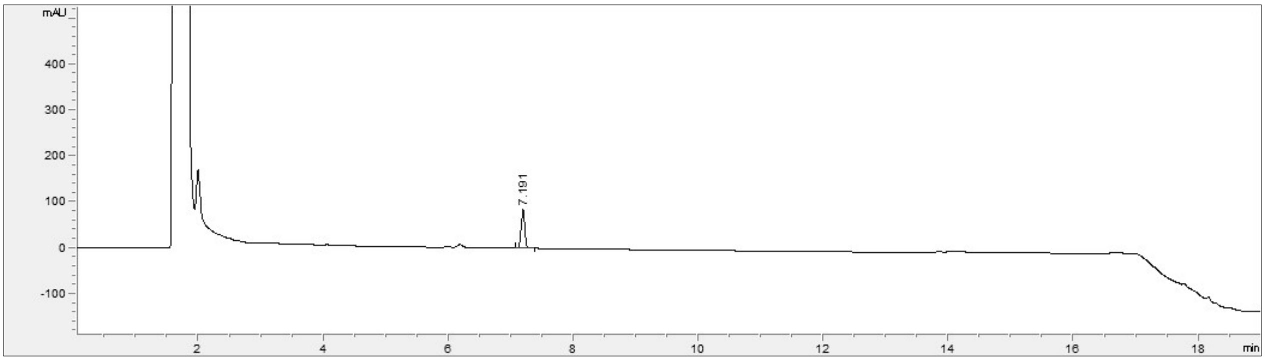

20-60% B, 15 min

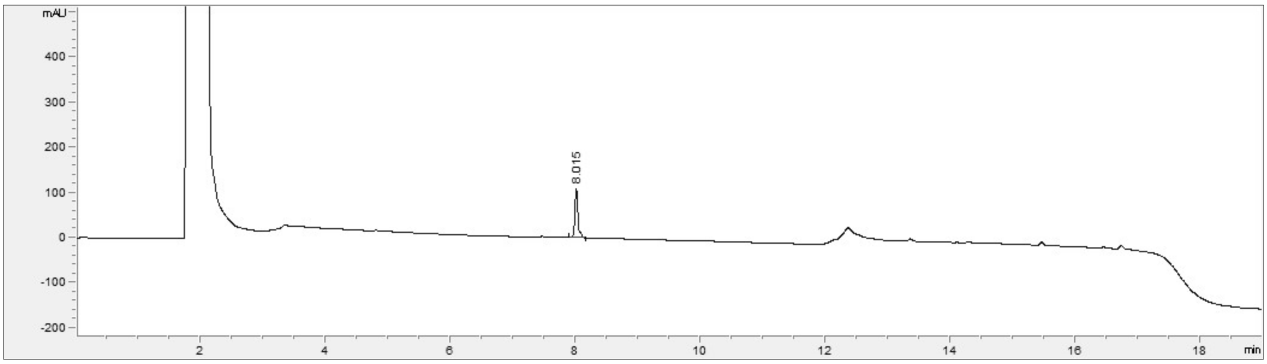

5-95% B, 15 min

P7C8

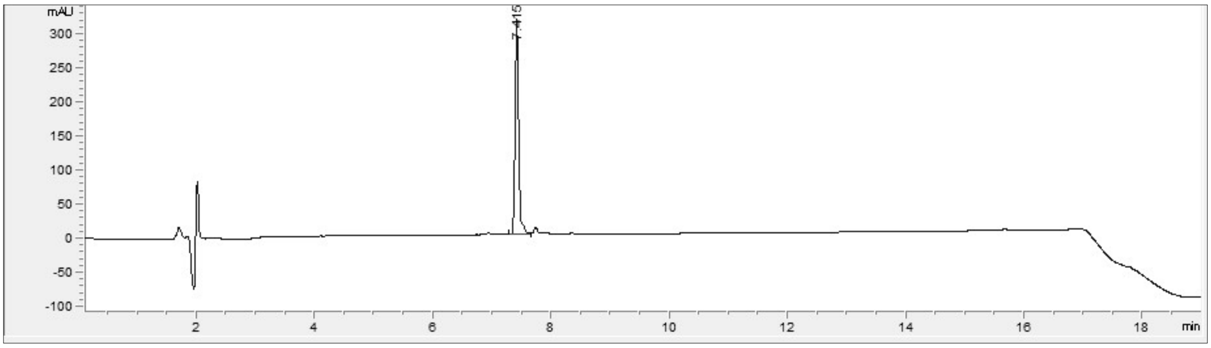

20-60% B, 15 min

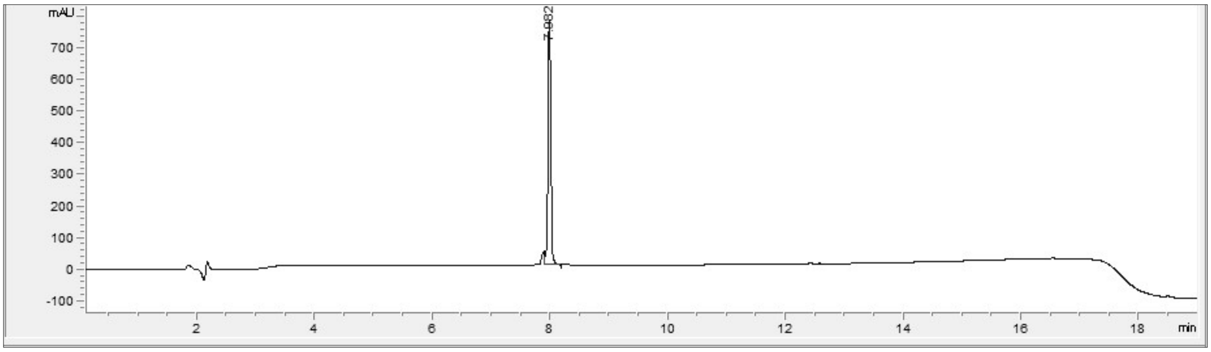

5-95% B, 15 min

P8C9

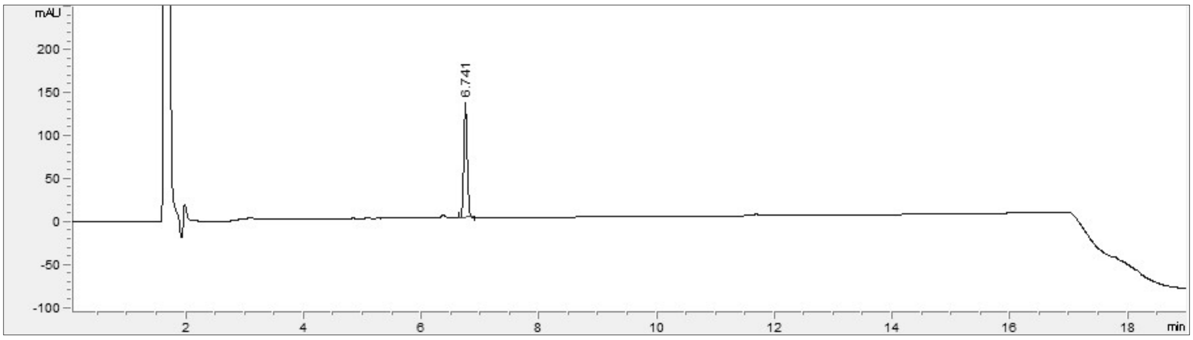

20-60% B, 15 min

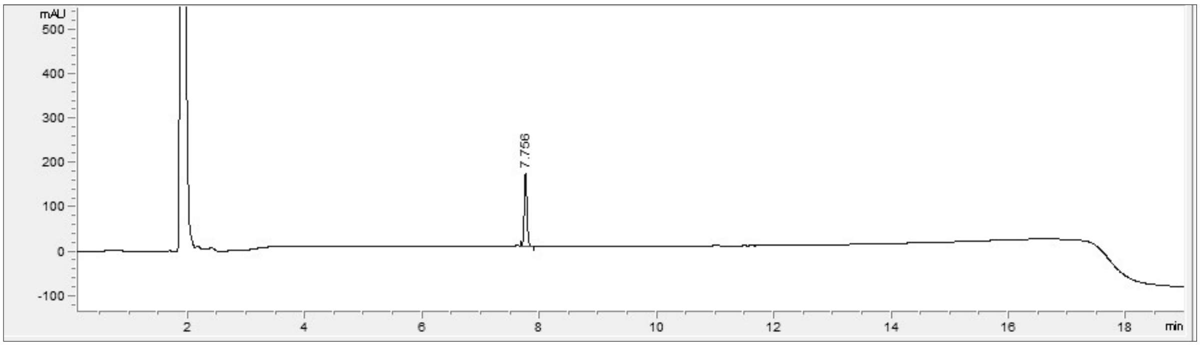

5-95% B, 15 min

FA-P8

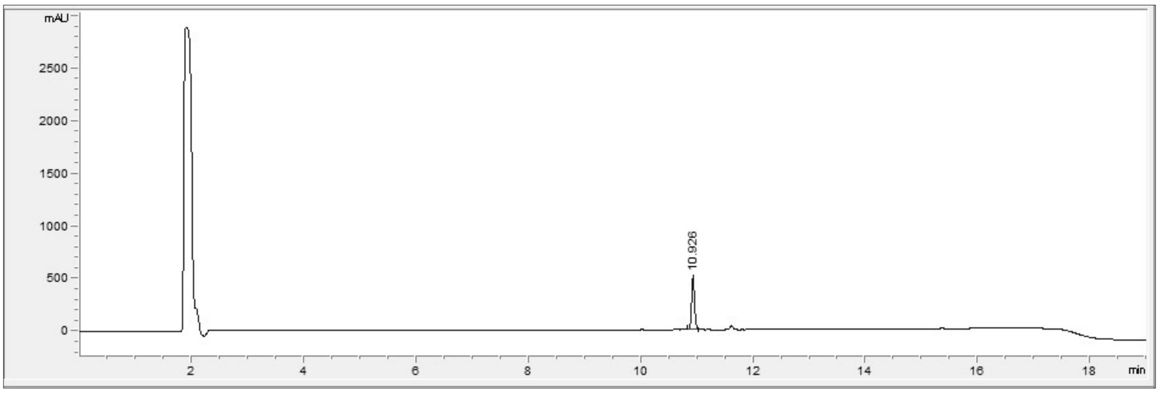

5-95% B, 15 min

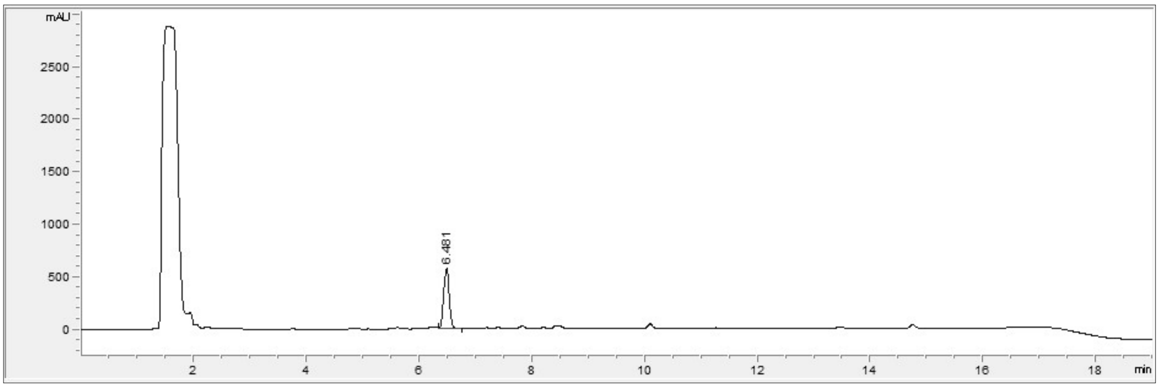

40-80% B, 15 min

FA-P8C9

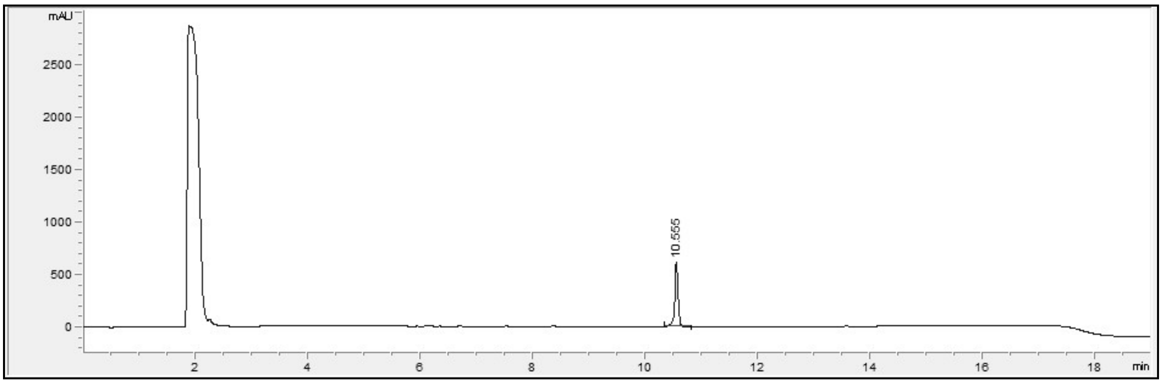

5-95% B, 15 min

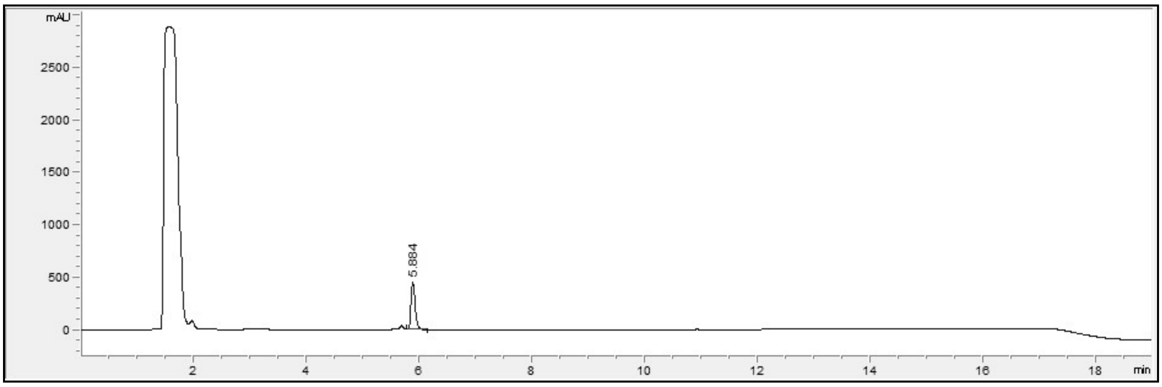

40-80% B, 15 min

PEG-P8

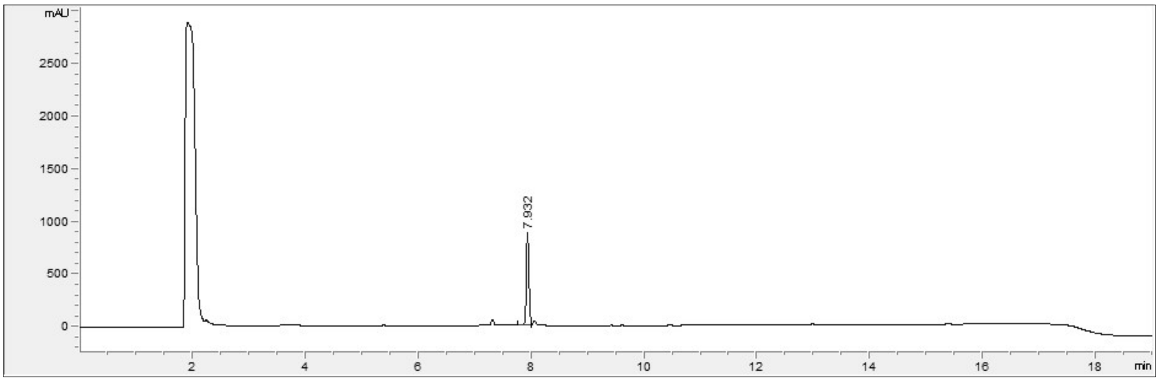

5-95% B, 15 min

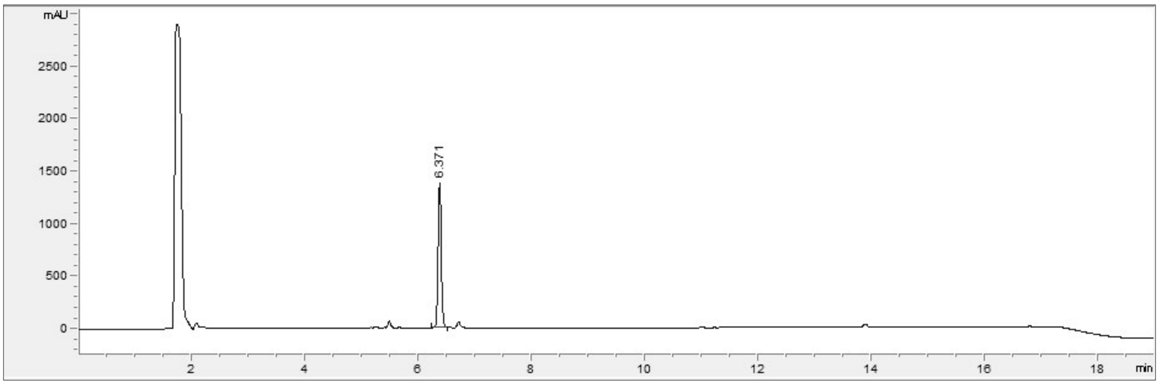

20-80% B, 15 min

PEG-P8C9

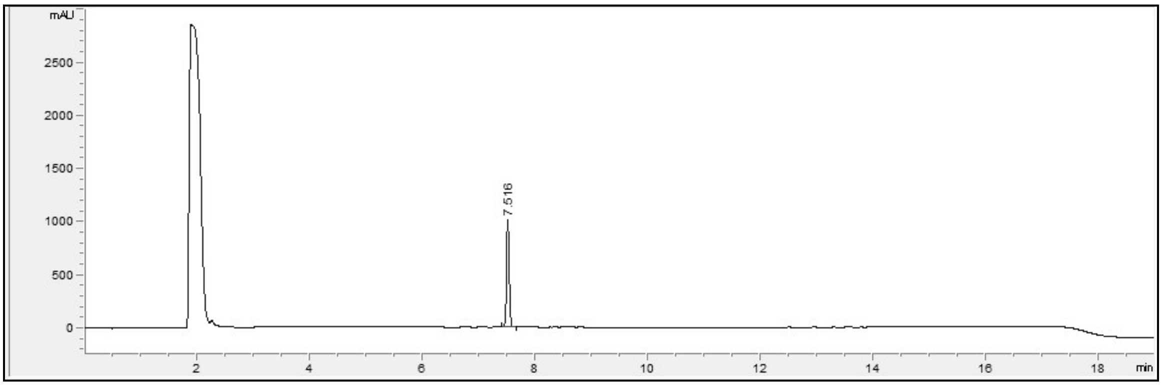

5-95% B, 15 min

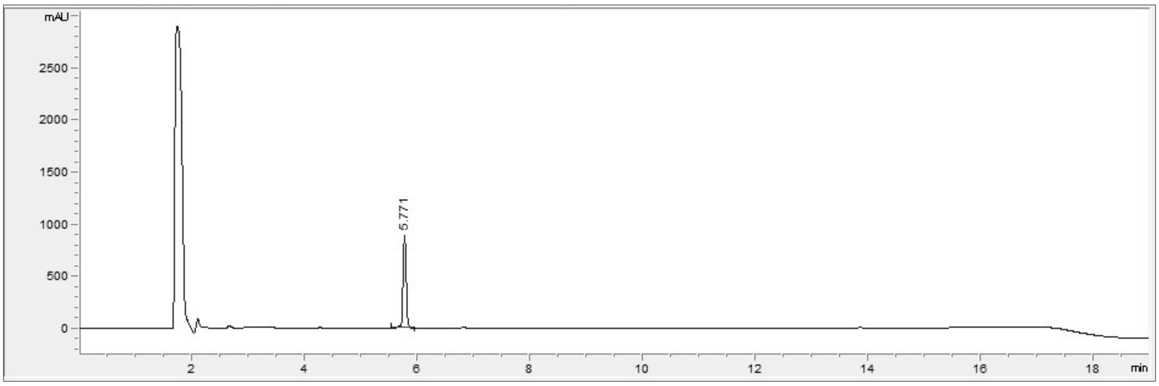

20-80% B, 15 min

P8[FA]

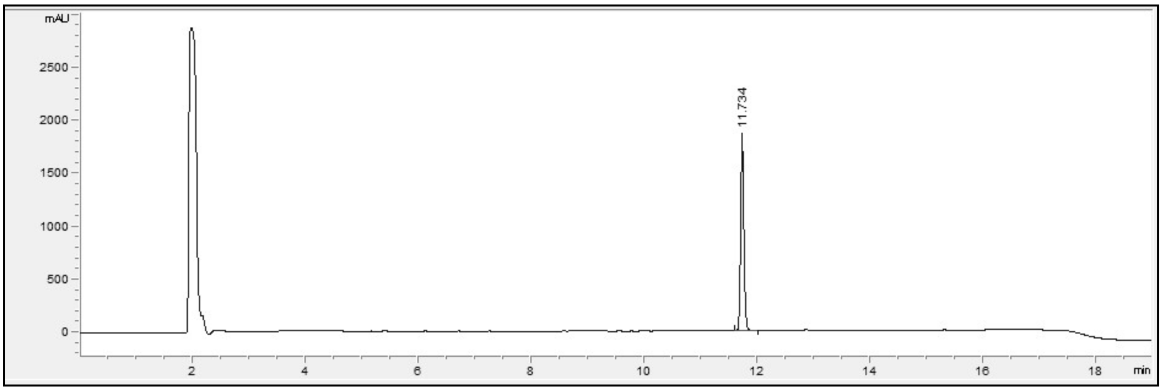

5-95% B, 15 min

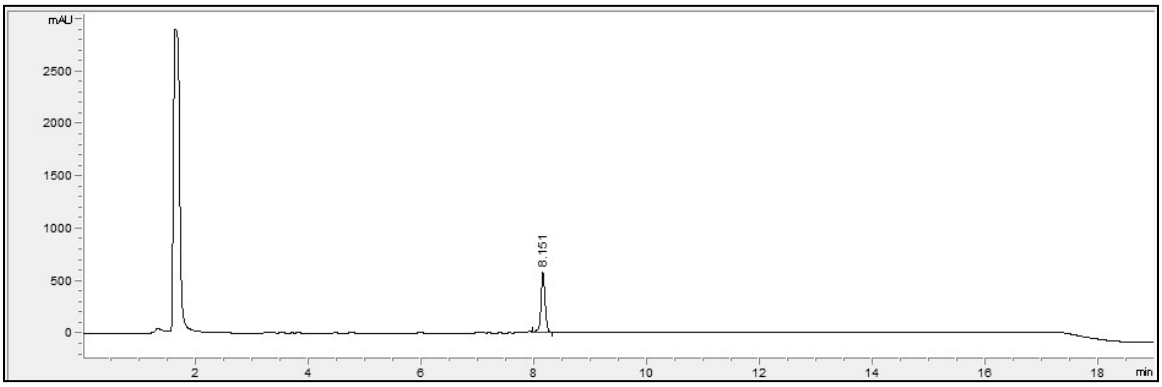

40-80% B, 15 min

P8C9[FA]

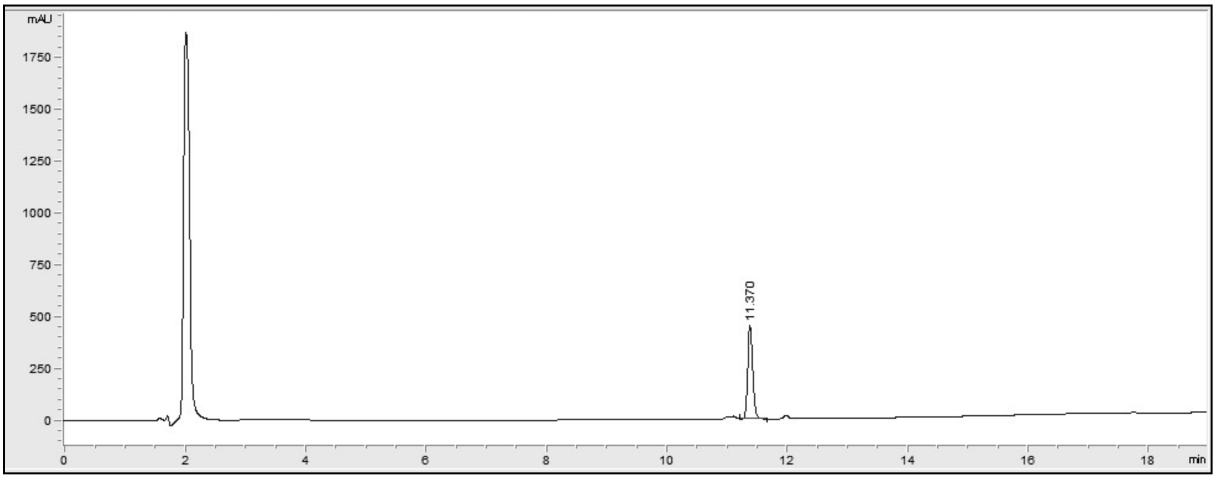

5-95% B, 15 min

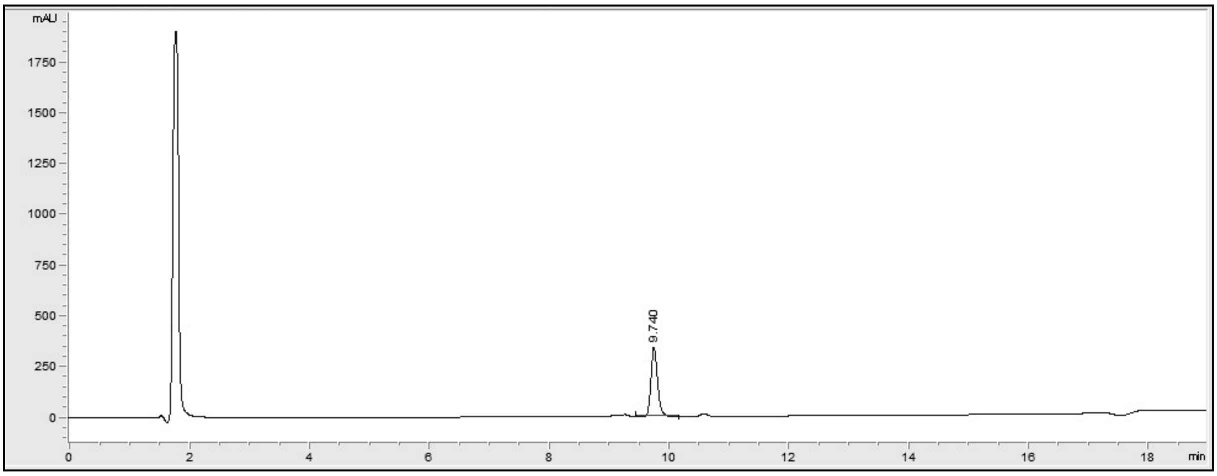

30-80% B, 15 min

P8[PEG]

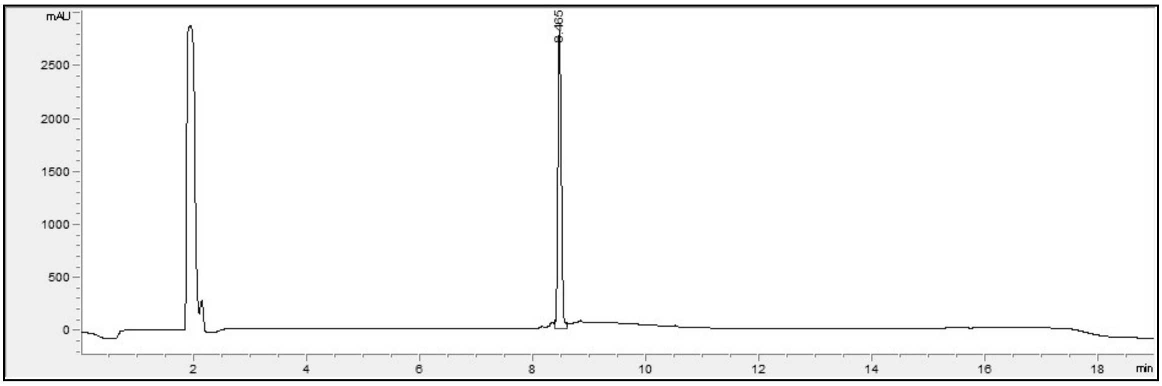

5-95% B, 15 min

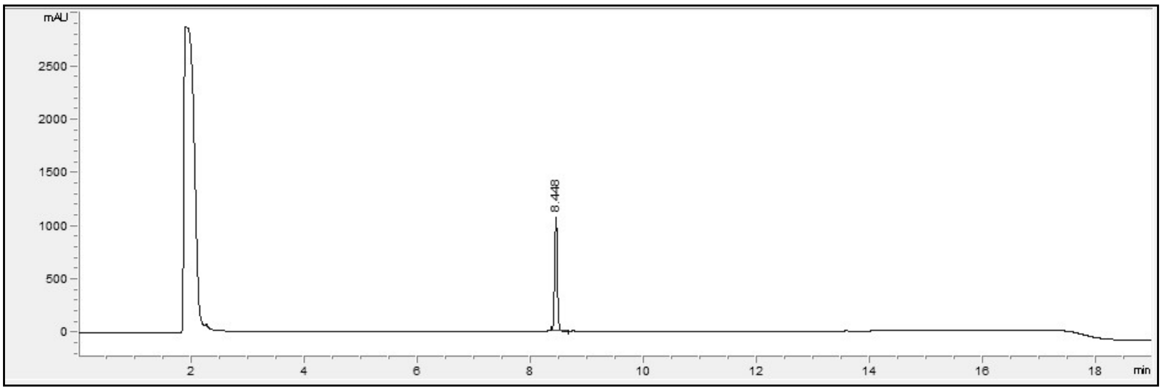

10-60% B, 15 min

P8C9[PEG]

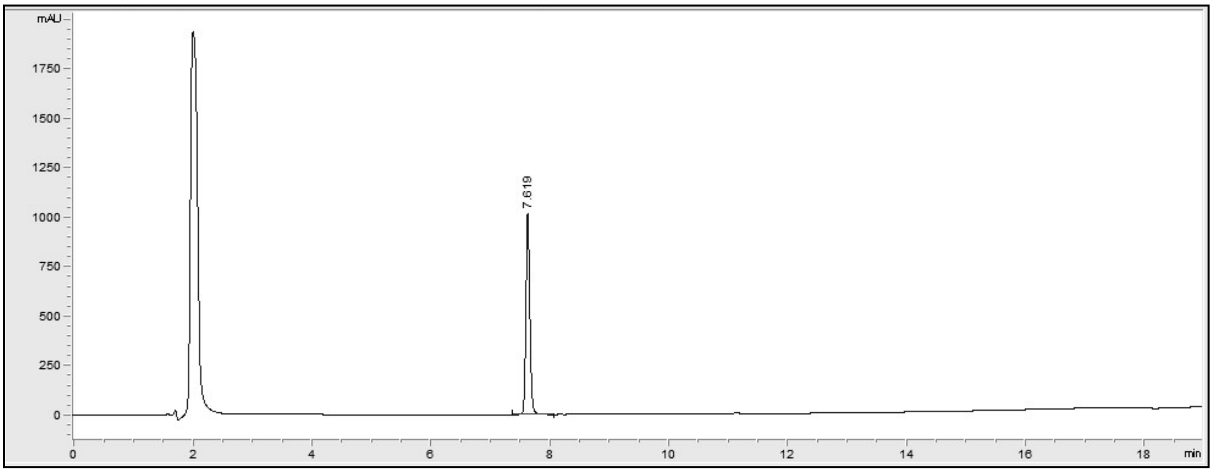

5-95% B, 15 min

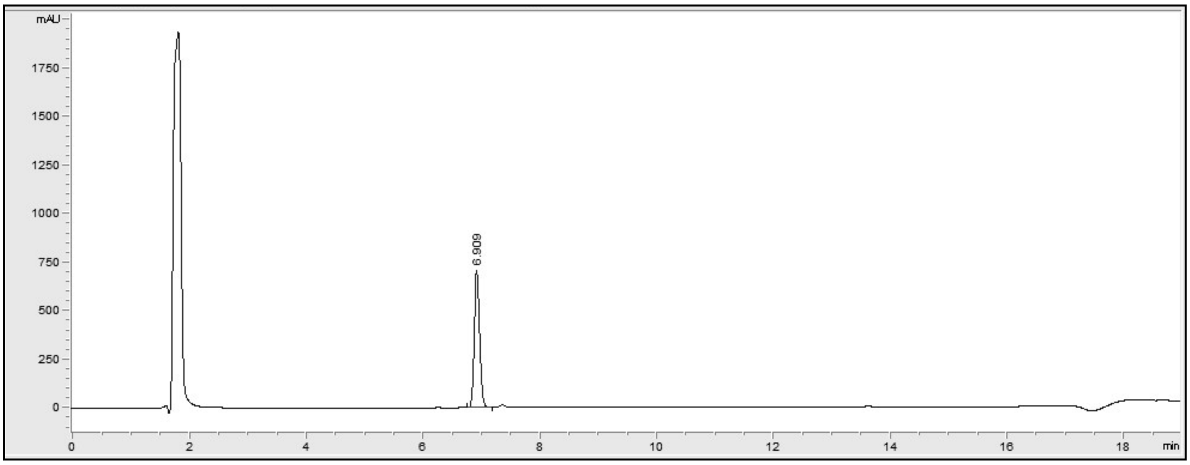

20-60% B, 15 min

TAT-P8

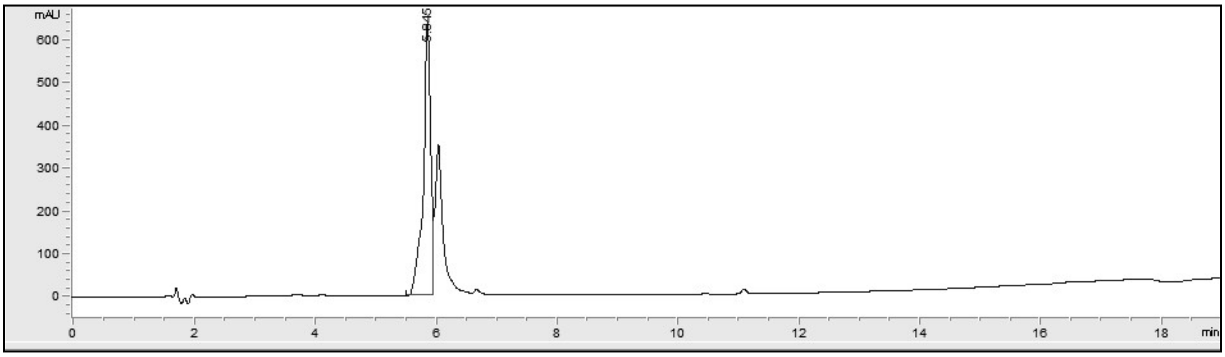

5-95% B, 15 min

TAT-P8C9

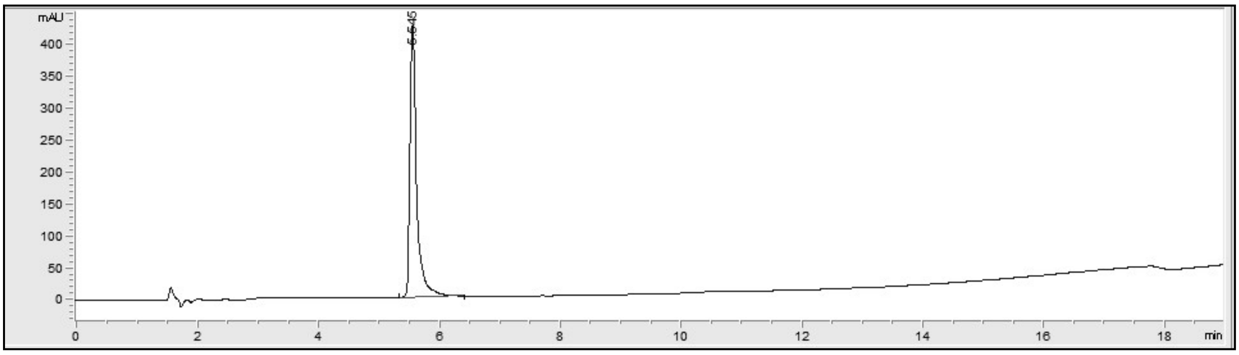

5-95% B, 15 min

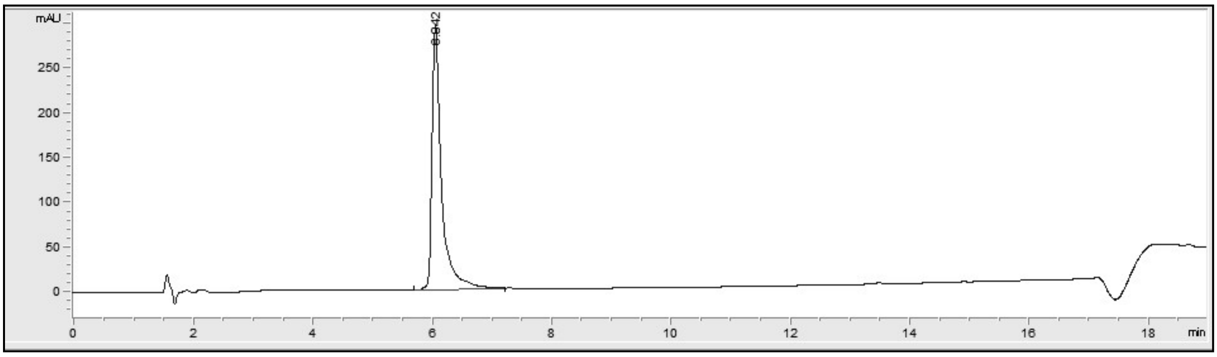

10-60% B, 15 min

TAT-$(Ahx)_2$

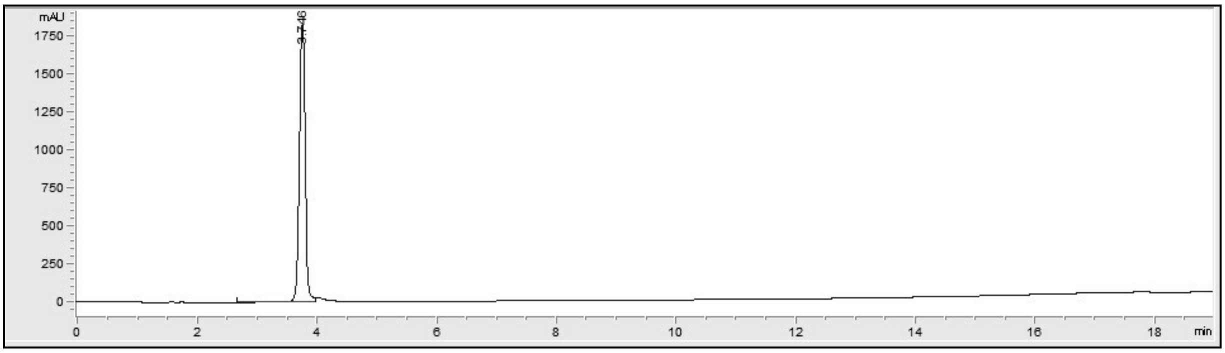

5-95% B, 15 min

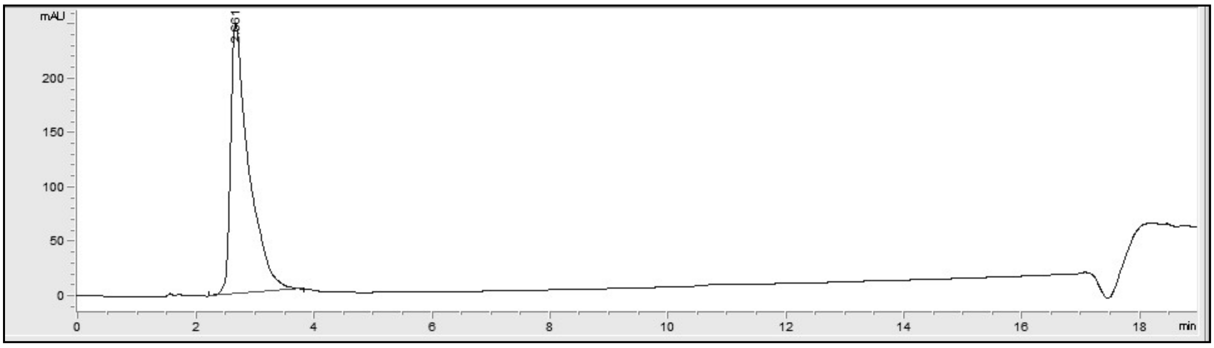

10-60% B, 15 min

R3-P8

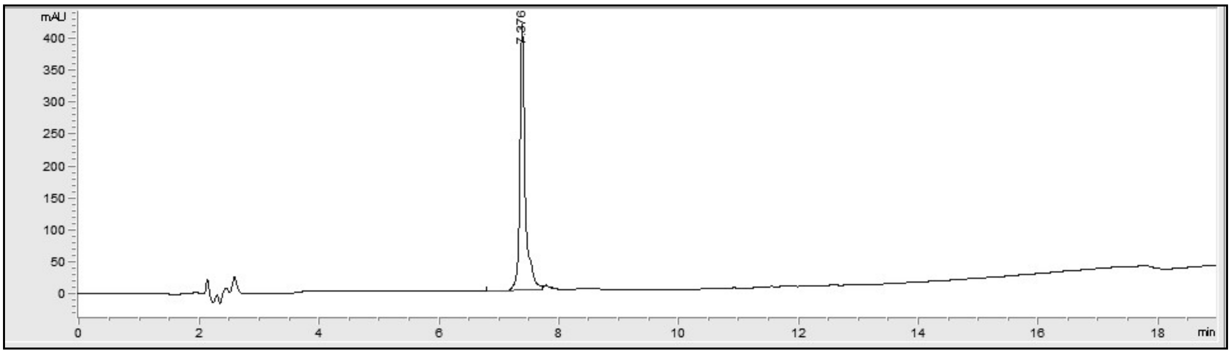

5-95% B, 15 min

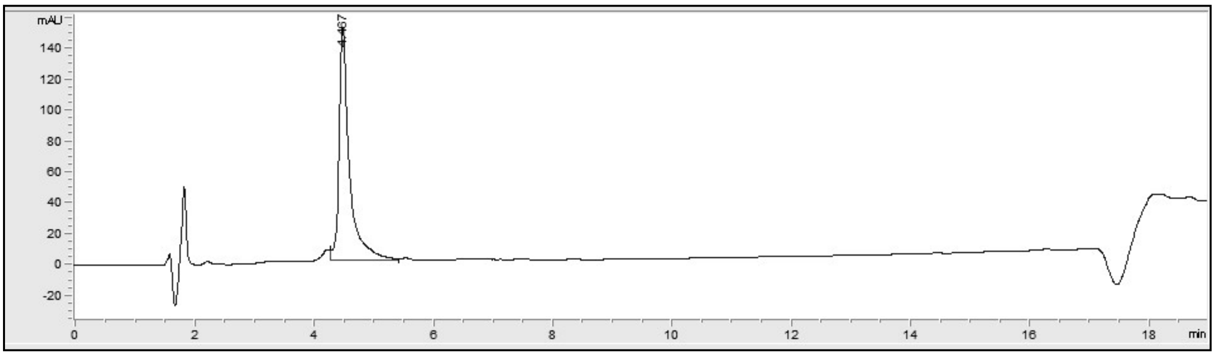

20-60% B, 15 min

R3-P8C9

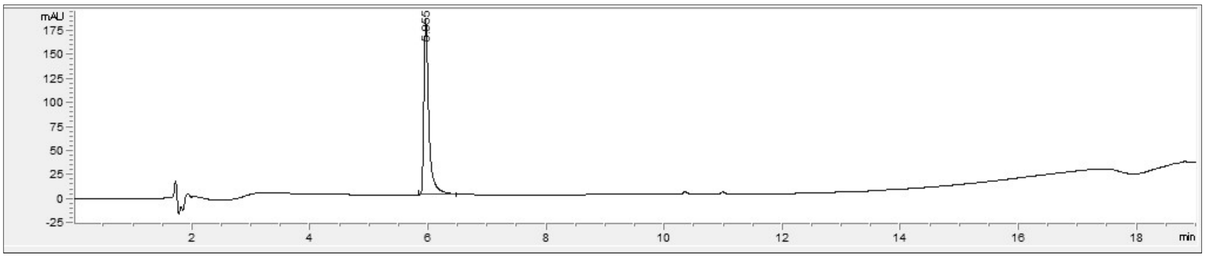

5-95% B, 15 min

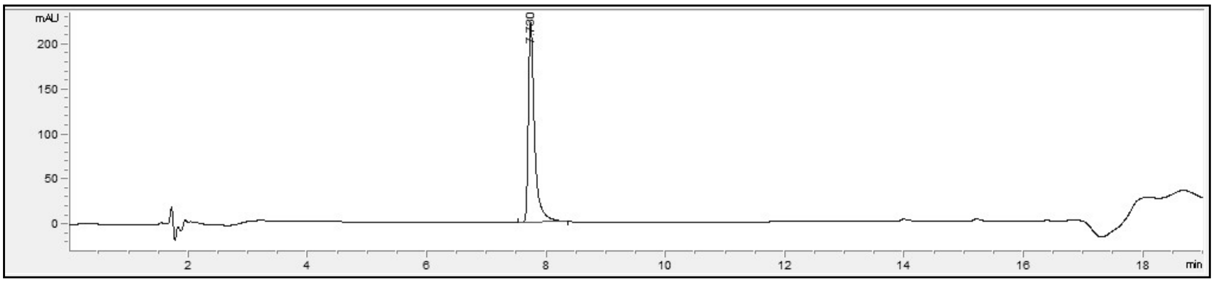

20-60% B, 15 min

R3-(Ahx)$_2$

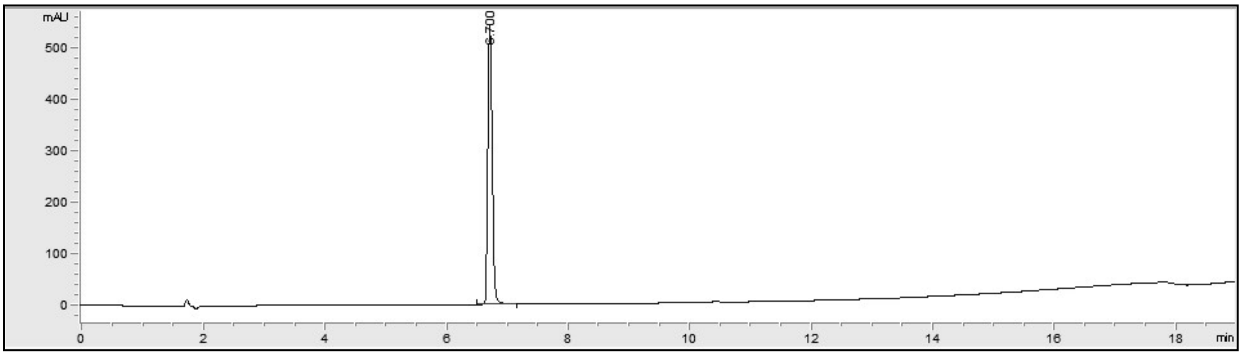

5-95% B, 15 min

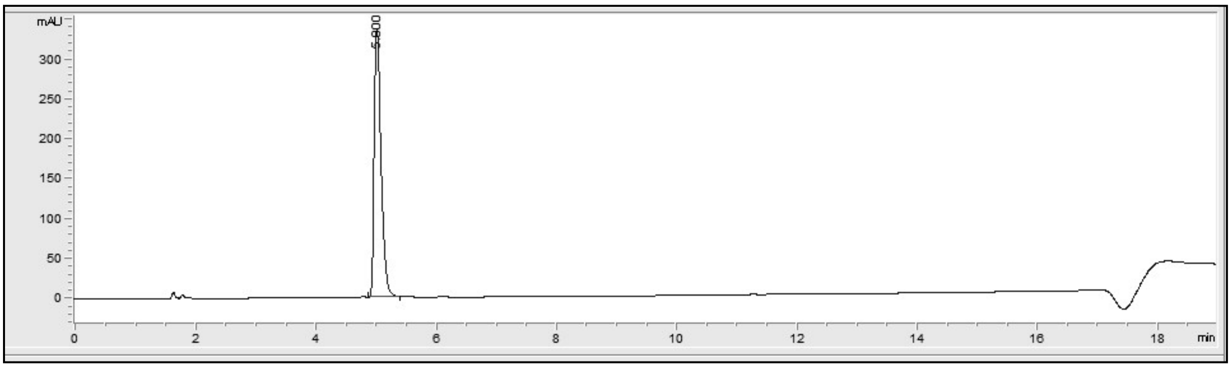

20-60% B, 15 min

1.3 Molecular modelling of cyclic peptides

The structures of the cyclic peptides were modelled on the crystal structure of CAM7117 bound to CK2α (PDB code: 6Q38) using the Maestro suite, produced by Schrödinger (version 12.6.149, Schrödinger, LLC, New York, NY). The constraint of CAM7117 was removed, along with the residues not retained in the shortened sequences, and the new constraint to be tested added using the build fragment function. The structure of the constraint was minimised with the OPLS_2005 force field, using 100 iterations.[15,16] Of those investigated, the constraints that appeared not to disrupt the secondary structure of the peptide were chosen for further investigation.

1.4 Ligand efficiency

Ligand efficiency (LE) is a commonly used statistic to evaluate and compare the effectiveness of drug candidates.[17] Mathematically, it is related to the IC_{50} of the molecule, as well as the number of heavy atoms (non-hydrogen) it contains, as detailed in **Equation 1**.[18]

Equation 1 Mathematical expression for the ligand efficiencies of compounds.[18] $pIC_{50} = -\log_{10}(IC_{50})$

$$LE = \frac{1.4 \times pIC_{50}}{Number\ of\ non-H\ atoms}$$

LE takes into account both the potency and size of molecules, to analyse which compounds exhibit the greatest activity with the fewest heavy atoms.

1.5 Biophysical experiments

1.5.1 Protein expression and purification

Three constructs of CK2α were used in this study. For FP assays, CK2α_WT was used (residues 2-329). For ITC CK2α_KA construct was used whilst CK2α_FP10 was used for crystallisation purposes. CK2α_KA (residues 2-329) contained four mutations designed to aid crystallisation and solubility by reducing the overall charge of the protein; R21S, K74A, K75A and K76A. CK2α_FP 10 contained one mutation (R21S) and an *N*-terminal extension GSMDIEFDDDADDDGSGSGSGSGS aimed at mimicking a substrate peptide for CK2α. These constructs were cloned, expressed, and purified as previously described.[19]

1.5.2 Fluorescence polarisation

IC_{50}'s were determined using a PHERAstar FS plate reader (BMG labtech). The fluorescein probe was measured using 485 nm excitation and 530 nm emission. The fluorescein probe was covalently linked to the *N*-terminus of the linear CK2β-based peptide RLYGFKIHPMAYQLQ. IC_{50}'s were measured using 500, 167, 16.7, 8.33 and 0 µM of the test peptide at a constant concentration of DMSO. **P7C8**, **P8C9**, **P_{+L}** and **P_{+}** were tested at concentrations of 50.0, 16.7, 1.67, 0.833 and 0 µM due to their higher potencies. The experiments were performed in a Corning 384-well plate with final concentrations of 571 nM CK2α_WT (residues 2-329), 7.4 µM fluorescent peptide (fluorescein probe was covalently linked to the *N*-terminus of the linear CK2β-based peptide RLYGFKIHPMAYQLQ), 200 mM NaCl, 40mM TRIS, 20 mM $MgCl_2$ and pH 7.5. The plates were read after a 45 min incubation period.

1.5.3 Isothermal titration calorimetry

All ITC experiments were performed at 25 °C using a MicroCal ITC-200 (GE Healthcare). CK2α_KA (20 mg/mL, 20 mM tris pH 8.0, 500 mM NaCl) was diluted in Tris buffer (200 mM, NaCl 300 mM, 10% DMSO) and concentrated to 20-50 µM. Compounds in 100× stock solutions were diluted into the same

buffer. In a typical experiment CK2α (25 µM) was loaded into the sample cell and 19 injections (2 µL each) with a 2 second duration were performed at 150 second intervals. The syringe was loaded with 200-250 µM peptides and rotated at 750 rpm. Control titrations were performed, and the data fitting was performed with a single site binding model using Origin software.

1.5.4 X-ray crystallography

Co-crystals of CK2α and stapled peptide were generated by screening CK2α_FP10 at 10 mg/mL in 20 mM Tris, pH 8.0, 500 mM NaCl and 500 µM stapled peptide with the BCS, JCSG+ and Wizard I&II crystallisation screens (molecular dimensions). Crystallography was performed as previously described.[4] All coordinates have been deposited to Protein Data Bank and accession numbers, data collection and refinement statistics are shown in Table S4.

Table S4 X-ray crystallography details including accession numbers, data collection and refinement statistics.

	**P7C8**	**P8C9**
pdb code	7QUX	6YZH
Beamline	i04	i03
Wavelength	0.9795	0.9762
Resolution range	55.66 - 1.48 (1.533 - 1.48)	55.19 - 1.19 (1.19 - 1.22)
Space group	P 1 21 1	P 1 21 1
Unit cell	57.461 63.25 58.672 90 108.439 90	56.51 65.038 59 90 111.21 90
Total reflections	1135374 (56487)	3079974 (94416)
Unique reflections	66313 (3314)	128134 (6408)
Multiplicity	17.1 (17.0)	24.0 (14.7)
Completeness (%)	99.7 (99.1)	94.1 (56.0)
Mean I/sigma(I)	18.0 (0.6)	22.8 (1.4)
Wilson B-factor	25.6	14.22
R-merge	0.076 (4.711)	0.063 (1.520)
R-meas	0.079 (5.090)	0.066 (1.630)
R-pim	0.019 (1.220)	0.018 (0.574)

CC1/2	1.0 (0.326)	1.0 (0.603)
Refinement		
Resolution range	55.66-1.48 (1.518-1.480)	55.19 - 1.11 (1.19 - 1.22)
Reflections used in refinement	62522 (4296)	110272(4195)
Reflections used for R-free	3442 (262)	5925(218)
R-work	0.1797 (0.398)	0.172(0.321)
R-free	0.2037 (0.359)	0.190 (0.3310)
Number of non-hydrogen atoms	3231	3235
macromolecules	2893	2938
ligands	49	46
solvent	289	251
Protein residues	338	348
RMS(bonds)	0.015	0.015
RMS(angles)	1.85	1.65
Ramachandran favored (%)	95.77	96.78
Ramachandran allowed (%)	4.23	2.92
Ramachandran outliers (%)	0	0.29
Rotamer outliers (%)	0.64	2.86
Clashscore	3.62	2.55

Average B-factor	35.31	22.29
macromolecules	34.78	21.87
ligands	33.04	16.45
solvent	41	28.22

1.6 Cellular biology

1.6.1 Cell culture

HeLa cells were maintained in 5% CO_2 in DMEM supplemented with 10% FBS, 2 mM L-glutamine, 100 U/mL penicillin and 100 mM streptomycin, in an atmosphere containing 5% CO_2.

Cells were regularly passed and used for experiments within one month after thawing (10-15 passages).

1.6.2 Cell viability assay

Cell viability was assayed by 3-(4,5-dimethylthiazol-2-yl)-2,5-diphenyltetrazolium bromide (MTT) colorimetric assay. HeLa cells were plated on 96-well plates (20,000 cells per well in 100 µL). After 24 h, cells were treated with different concentrations of test compounds or vehicle alone (DMSO). One hour before the end of the incubations, 10 µL of MTT solution (5 mg/mL in PBS) were added to each well. Formazan crystals (proportional to the number of viable cells) were then dissolved by the addition of 20 µL/well of a pH 4.7 solution containing 20% (w:v) SDS, 50% (v:v) *N*,*N*-dimethylformamide, 2% (v:v) acetic acid, and 25 mM HCl, and quantified by recording the absorbance at 570 nm using an Infinite M200 PRO plate reader (TECAN, Life Sciences). The test was performed in quadruplicate.

1.6.3 Serum stability test

500 µL of PBS buffer supplemented with 20% (v/v) of human serum was allocated into an Eppendorf tube and temperature kept at 37 °C for 15 minutes before commencing the experiment. 5 µL of the peptide from 10 mM stock solution in DMSO was added. Caffeine was added as an internal standard (10 µL of a 15 mg/mL solution in MQ water). At specific intervals, 50 µL of the reaction mixture was taken and quenched with 100 µL of a 1:1 mixture of 96% Ethanol:DMSO. The suspension was spun at 13400 g for 10 minutes. 100 µL of the supernatant was analysed using C-18 HPLC with an eluting gradient 5-95% MeCN (0.05% TFA) in water (0.05% TFA) over 15 minutes (90 µL injection volume). Percentage of intact starting peptide was monitored over 24 h (calculated as the ratio of the area of

the peak corresponding to the intact peptide to the area of the peak of caffeine). The experiment was performed in duplicates.

1.7 References

1 M. M. Wiedmann, Y. S. Tan, Y. Wu, S. Aibara, W. Xu, H. F. Sore, C. S. Verma, L. Itzhaki, M. Stewart, J. D. Brenton and D. R. Spring, *Angew. Chemie Int. Ed.*, 2017, **56**, 524–529.

2 Y. Lau and D. Spring, *Synlett*, 2011, **2011**, 1917–1919.

3 G. T. Potter, G. C. Jayson, G. J. Miller and J. M. Gardiner, *J. Org. Chem.*, 2016, **81**, 3443–3446.

4 J. Iegre, P. Brear, D. J. Baker, Y. S. Tan, E. L. Atkinson, H. F. Sore, D. H. O' Donovan, C. S. Verma, M. Hyvönen and D. R. Spring, *Chem. Sci.*, 2019, **10**, 5056–5063.

5 K. Matsuda, M. T. Stone and J. S. Moore, *J. Am. Chem. Soc.*, 2002, **124**, 11836–11837.

6 J. S. Parker, H. F. Sore, D. R. Spring and S. J. Walsh, *WO2020025108A1*, 2020-02–06.

7 C. Puig Duran, F. Albericio Palomera, M. Gongora Benitez, M. Paradis Bas, L. Miret Casals, I. Ramos Tomillero, S. Fiacco, A. Davis, S. Geschwindner, O. Brun Cubero, C. Heras Paniagua and N. Trallero Canela, *WO/2019/030298*, 2019-02–14.

8 Y. H. Lau, Y. Wu, P. de Andrade, W. R. J. D. Galloway and D. R. Spring, *Nat. Protoc.*, 2015, **10**, 585–594.

9 N. Assem, D. J. Ferreira, D. W. Wolan and P. E. Dawson, *Angew. Chemie Int. Ed.*, 2015, **54**, 8665–8668.

10 C. M. B. K. Kourra and N. Cramer, *Chem. Sci.*, 2016, **7**, 7007–7012.

11 N. S. Robertson, S. J. Walsh, E. Fowler, M. Yoshida, S. M. Rowe, Y. Wu, H. F. Sore, J. S. Parker and D. R. Spring, *Chem. Commun.*, 2019, **55**, 9499.

12 S. J. Walsh, J. Iegre, H. Seki, J. D. Bargh, H. F. Sore, J. S. Parker, J. S. Carroll and D. R. Spring, *Org. Biomol. Chem*, 2020, **18**, 4224.

13 S. Ingale and P. E. Dawson, *Org. Lett.*, 2011, **13**, 2822–2825.

14 M. Empting, O. Avrutina, R. Meusinger, S. Fabritz, M. Reinwarth, M. Biesalski, S. Voigt, G. Buntkowsky and H. Kolmar, *Angew. Chemie Int. Ed.*, 2011, **50**, 5207–5211.

15 W. L. Jorgensen, D. S. Maxwell and J. Tirado-Rives, *J. Am. Chem. Soc.*, 1996, **118**, 11225–11236.

16 E. Harder, W. Damm, J. Maple, C. Wu, M. Reboul, J. Y. Xiang, L. Wang, D. Lupyan, M. K.

Dahlgren, J. L. Knight, J. W. Kaus, D. S. Cerutti, G. Krilov, W. L. Jorgensen, R. Abel and R. A. Friesner, *J. Chem. Theory Comput.*, 2016, **12**, 281–296.

17 A. L. Hopkins, G. M. Keserü, P. D. Leeson, D. C. Rees and C. H. Reynolds, *Nat. Rev. Drug Discov.*, 2014, **13**, 105–121.

18 M. D. Shultz, *Bioorg. Med. Chem. Lett.*, 2013, **23**, 5980–5991.

19 P. Brear, C. De Fusco, K. Hadje Georgiou, N. J. Francis-Newton, C. J. Stubbs, H. F. Sore, A. R. Venkitaraman, C. Abell, D. R. Spring and M. Hyvönen, *Chem. Sci.*, 2016, **7**, 6839–6845.
